# Synthesis of dihydroquinazolines from 2-aminobenzylamine: *N*^3^*-*aryl derivatives with electron-withdrawing groups

**DOI:** 10.3762/bjoc.14.227

**Published:** 2018-09-26

**Authors:** Nadia Gruber, Jimena E Díaz, Liliana R Orelli

**Affiliations:** 1 Universidad de Buenos Aires, CONICET, Departamento de Química Orgánica, Facultad de Farmacia y Bioquímica, Junín 956, (1113) Buenos Aires, Argentina

**Keywords:** cyclodehydrations, dihydroquinazolines, microwaves, nitrilium ions, PPSE, S_N_Ar

## Abstract

The sequential *N-*functionalization of 2-aminobenzylamine (2-ABA) followed by cyclodehydration allowed for a straightforward and efficient synthesis of 3,4-dihydroquinazolines with *N*-aryl substituents bearing electron-withdrawing groups. The sequence involves an initial S_N_Ar displacement, *N-*acylation and MW-assisted ring closure. Remarkably, the uncatalyzed *N-*arylation of 2-ABA led to the monosubstitution product using equimolar amounts of both reagents. The individual steps were optimized achieving good to excellent overall yields of the desired heterocycles, avoiding additional protection and deprotection steps. A mechanistic interpretation for the cyclodehydration reaction promoted by trimethylsilyl polyphosphate (PPSE) is also proposed on the basis of literature data and our experimental observations.

## Introduction

**Figure 1:**
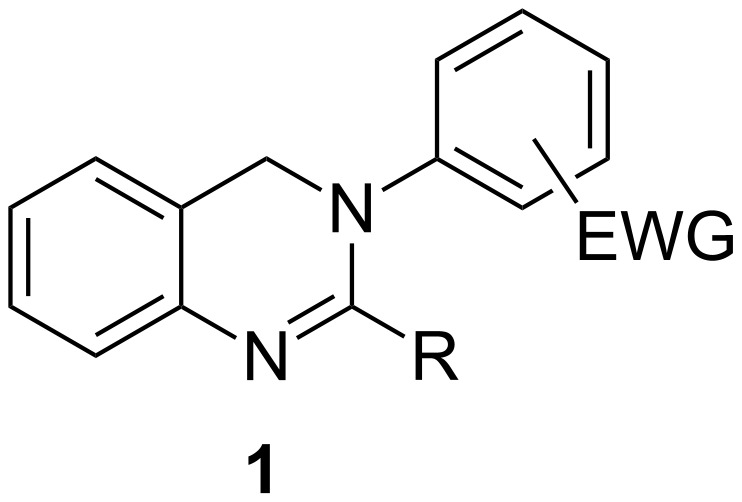
*N*-Aryl-3,4-dihydroquinazolines **1**.

Dihydroquinazolines (DHQs) represent heterocyclic cores of pharmacological interest. For example, vasicine is a 3,4-dihydroquinazoline alkaloid isolated from natural sources with antitumor activity [1]. Vasicine and deoxyvasicine are also potent butyrylcholinesterase (BChE) inhibitors [2]. Some synthetic derivatives containing the dihydroquinazoline scaffold have shown antimicrobial [3] and antifungal properties [4]. In addition, antiparasitic activity has been studied for some members of this family as inhibitors of trypanothione reductase [5], an essential enzyme of the kinetoplastid *Trypanosoma brucei.* Their activity as selective T-type calcium channel blockers [6–11], as tumor suppressors [12] and as neuroprotective agents has also been reported [13]. Some related 2-amino-DHQs were studied as blood platelet aggregation inhibitors [14], antihypertensive agents [15] or inhibitors of β*-*secretase, an important target for Alzheimer’s disease [16]. Additionally, some ruthenium complexes bearing a 3,4-dihydroquinazoline ligand have been studied as hydrogenation-transfer catalysts, showing good to excellent activity for the reduction of ketones [17]. In the context of our research on heterocyclic amidine *N-*oxides [18–22], we recently prepared some suitably 2-substituted *N-*aryl-3,4-DHQs with electron-withdrawing groups (EWGs) in their aryl moiety (Figure 1) as synthetic intermediates for novel tetracyclic nitrones, to be studied as spin traps and antimicrobials.

The literature describes several methods for the synthesis of 3,4-DHQs. Some of them involve oxidation or reduction of related heterocycles (quinazolinones, quinazolines or tetrahydroquinazolines) [23–28]. Dihydroquinazolines can also be synthesized by heterocyclization of 2-aminobenzylamines (2-ABAs) with different C2 donors [3,16–17,29–36]. An alternative method is the ring closure of *N-*acyl-2-ABAs, but this strategy generally requires high temperatures and/or long reaction times [5,37–40].

Recently, our group described efficient strategies to prepare 3,4- and 1,4-DHQs with different substitution patterns from 2-ABA [41]. However, *N*-aryl-3,4-DHQs were not accessible through these routes. There are very few reported strategies for the synthesis of C4-unsubstituted *N-*aryl-3,4-DHQs, and all of them show limitations. The classical method involves the reaction between *p*-substituted anilines and paraformaldehyde in mineral [42–44] or formic [45] acid to yield symmetrically substituted 3,4-DHQs, usually in low yields [45] and accompanied by several by-products [46–47]. In addition, this strategy is limited to the preparation of 3,6-disubstituted compounds, because a *p*-substituted aniline is required to block this condensation position [48–50]. An exception to this is the recently reported synthesis of 3-(*m*-nitrophenyl)-5-nitro-3,4-dihydroquinazoline from *m*-nitroaniline and 1,3-dioxolane in the presence of strong protic acids [51]. In a related methodology, the ring closure of *N*-aryl-2-ABA is promoted by formic acid or other sources of C2 like ethyl orthoformate [52], diarylformamidines [53] or 1,1-dimethoxy-*N*,*N*-dimethylmethanamine [17]. Using an alternative approach, the corresponding 2-ABA was treated with acetic anhydride in concentrated sulfuric acid affording 2-methyl-6-nitro-3-(*p*-nitrophenyl)-3,4-DHQ [54]. *o*-Nitrobenzylanilines were also employed as starting materials, although this methodology involves a reduction step to form the functionalized 2-ABA and suffers from byproduct formation [55–57]. A different strategy involves the oxidation of *N-*aryl-1,2,3,4-tetrahydroquinazolines [45,58]. A more recent method starts from an aromatic amine and formaldehyde in the presence of ionic liquids and a controlled amount of 1-methyl-3-(2-(sulfoxy)ethyl)-1*H*-imidazol-3-ium chloride as the catalyst. The reaction affords the corresponding 1,4-dihydroquinzolin-3-ium tetrafluoroborates, which upon treatment with NaOH/EtOH evolve to the symmetrically substituted 3,4-DHQs [59]. Other authors report the synthesis of *N*-phenyl-3,4-DHQ by alkylation of sodium formanilide with 2-nitrobenzyl chloride followed by cyclization under reductive conditions [60], or from 2-ABA and CO_2_ as starting materials in catalytic reductive conditions [61].

In conclusion, the already described methods for the synthesis of 4-unsustituted *N*-aryl-3,4-DHQs are either limited to symmetrical substitution patterns, lead to byproducts and afford low yields, require the use of complex catalysts and ionic liquids or involve reductive conditions that are incompatible with nitro or cyano groups. In this context, and as part of our ongoing research on heterocyclic amidines (vide infra) and amidine *N-*oxides we report herein the first method for the synthesis of 2-alkyl/aryl-*N-*aryl-3,4-DHQs **1** bearing electron-withdrawing groups in the aryl moiety.

Our approach involves two selective functionalizations of 2-ABA followed by a ring-closure step. Some selective *N-*acylations and *N-*alkylations of this precursor had been described in the literature, involving in many cases additional protection and deprotection steps or affording modest yields [40,62–67]. *N-*Arylations of this substrate have been attempted, although with poor results [68]. Alternative strategies leading to *N-*functionalized 2-ABAs start from isatoic anhydride, 2-nitrobenzonitrile, 2-nitrobenzaldehyde, 2-aminobenzophenone or 2-nitrobenzyl halides, among others, and involve a reduction step which would be incompatible with the presence of nitro or cyano groups [5,13–14,39,68–71].

The synthetic sequence towards compounds **1** requires the chemoselective arylation of the benzylic amino group of the precursor with active haloaryl derivatives. S_N_Ar is a widely employed reaction in organic synthesis [72]. However, the possibility of achieving selective *N-*substitution of diamines in equimolar conditions with high yields still represents a challenge. Selective S_N_Ar reactions were reported for polyhaloaryl substrates [73–74], while alkanediamines require in most cases either a large excess of the nucleophile [75–77] or Boc protection [78–79] in order to obtain the monoarylated product in acceptable yields. There are some reports on selective S_N_Ar reactions of piperazine [80] but, to the best of our knowledge, uncatalyzed selective *N-*arylations of unprotected primary diamines have not been systematically investigated yet.

Regarding the last step of the sequence leading to compounds **1**, our group has worked extensively on ring-closure reactions leading to nitrogen-containing heterocycles such as 5–8-membered cyclic amidines [81–83], *N-*aryl-2-iminoazacycloalkanes [84] and 2-oxazolines or their higher homologues [85], using polyphosphoric acid esters PPE (ethyl polyphosphate) [86] and PPSE (trimethylsilyl polyphosphate) [87] under microwave irradiation. PPE and PPSE are aprotic irreversible dehydrating agents of the Lewis acid-type, which are able at the same time, to activate nitrogen and oxygen functionalities towards nucleophilic attack. This dual behavior makes them valuable reagents, capable of promoting certain cyclodehydrations that cannot be achieved by using classical Lewis acids [84–85]. Additional advantages of PPA esters are their low cost, operational simplicity and minimum environmental impact. Besides, their use together with microwave irradiation brings about shorter reaction times, cleaner crude products and consequently higher yields. On the basis of our previous work on DHQs [41], we explore herein the use of PPA esters for the optimization of the heterocyclization reaction leading to compounds **1**.

## Results and Discussion

**Scheme 1:**
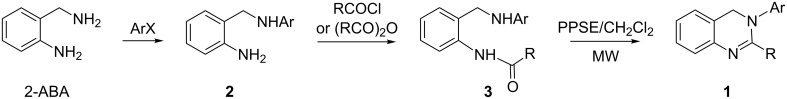
Synthetic pathway leading to *N*-aryl-3,4-dihydroquinazolines **1**.

The synthetic strategy leading to DHQs **1** is depicted in Scheme 1.

**Scheme 2:**
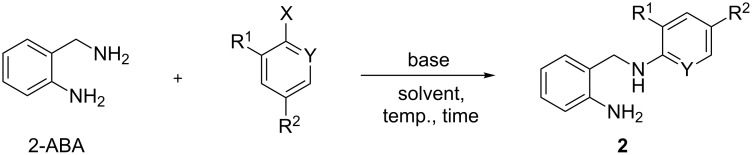
Synthesis of compounds **2**.

The key step of the sequence involves an *N*-arylation of 2-ABA with active haloaromatics (Scheme 2). As previously mentioned, S_N_Ar displacements in 1,*n*-diamines usually require a large excess of the nucleophile in order to achieve acceptable results. As 2-ABA has two nucleophilic nitrogen centers of different reactivity, the challenge consisted in achieving complete selectivity together with high yields using equimolar amounts of both reactants.

The reaction between 2-ABA and 2-nitrohalobenzenes was chosen for the initial optimization of the experimental conditions, whose results are collected in Table 1. No reaction was observed with 2-nitrochlorobenzene after 8 h either in solvent-free conditions at 125 °C or using THF at reflux, even with a two-fold excess of the amine (Table 1, entries 1–3). Better results were achieved with DME as the solvent and adding K_2_CO_3_ as proton acceptor (Table 1, entry 4). The replacement of 2-nitrochlorobenzene by the more reactive fluoro derivative resulted in a significant increase of the reaction yield (Table 1, entry 5). The best results were achieved using water as the solvent (Table 1, entry 6). Under such conditions, the conversion using equimolar amounts of the reagents was almost quantitative, with complete selectivity towards the *N*-monoarylation product **2a**.

**Table 1:** Reaction conditions screening for the synthesis of compounds **2**.

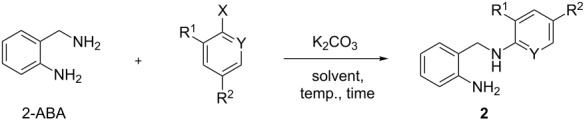									

Entry	**2**	R^1^	R^2^	Y	X	Solvent; mL/mmol ArX	Temperature (°C)	Time (h)	Yield (%)^a^

1^b^	**a**	NO_2_	H	CH	Cl	–	125	8	NR
2^b^	**a**	NO_2_	H	CH	Cl	THF; 0.5	reflux	8	NR
3^b,c^	**a**	NO_2_	H	CH	Cl	THF; 0.5	reflux	8	NR
4	**a**	NO_2_	H	CH	Cl	DME; 0.5	reflux	24	42
5	**a**	NO_2_	H	CH	F	DME; 0.5	reflux	4	90
6	**a**	NO_2_	H	CH	F	H_2_O; 1	80	4	96
7	**b**	H	NO_2_	CH	F	H_2_O; 1	80	4	52
8	**b**	H	NO_2_	CH	F	H_2_O; 1	80	16	58
9	**b**	H	NO_2_	CH	F	H_2_O; 1	reflux	4	71
10	**b**	H	NO_2_	CH	F	DMSO; 0.25	125	4	96
11	**c**	NO_2_	H	N	Cl	H_2_O; 1	80	8	20
12	**c**	NO_2_	H	N	Cl	H_2_O; 1	50	8	55
13	**c**	NO_2_	H	N	Cl	DMSO; 0.5	rt	8	quant.
14	**d**	CN	H	CH	F	DMSO; 0.25	125	8	66
15	**d**	CN	H	CH	F	DMSO; 0.25	135	8	68
16	**d**	CN	H	CH	F	DMSO; 0.25	150	8	53

^a^Yields correspond to pure compounds. ^b^The reaction was carried out without K_2_CO_3_. ^c^The reaction was carried out with a two-fold excess of 2-ABA.

In the same experimental conditions, the reaction of 2-ABA with 4-nitrofluorobenzene afforded acceptable yields (Table 1, entry 7), although complete conversion was not achieved even working at a higher temperature or with longer reaction times (Table 1, entries 8 and 9). DMF was then tested as the solvent in order to improve the yield by raising the temperature, but the reaction performed at 125 °C afforded *N,N-*dimethyl-4-nitroaniline as the main product together with a low percentage (<10%) of the desired compound **2b**. DMSO gave the best results due to its higher boiling point, stability and a better solubility of the reactants, leading to nearly quantitative yields of **2b** with complete chemoselectivity (Table 1, entry 10).

**Figure 2:**
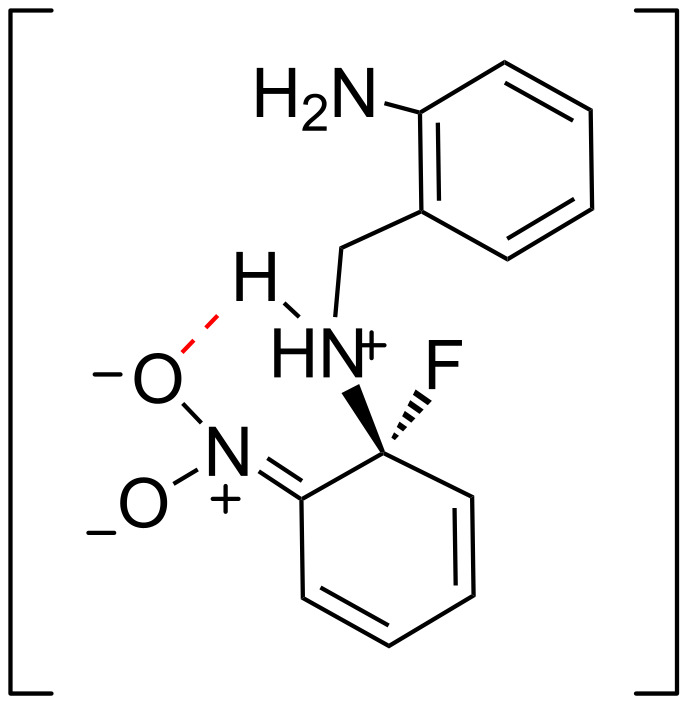
Reaction intermediate in the synthesis of compound **2a**.

The different reactivity of *o-* and *p-*nitrofluorobenzenes in S_N_Ar displacements had previously been reported, and is attributed to a differential stabilization of the reaction intermediate in the former. Such effect would result from an intramolecular hydrogen bond between the ammonium and nitro groups in the reaction intermediate, also known as “built-in solvation” [71] (Figure 2).

When 2-ABA was reacted with 2-chloro-3-nitropyridine (CNP) in analogous experimental conditions as **2a**, compound **2c** was isolated in low yield (Table 1, entry 11). A second product was obtained, which was identified as the corresponding *N*,*N*’*-*diaryl derivative. This behavior was related to the enhanced reactivity of CNP due to the presence of two strong EWGs in the aromatic ring. Lowering the reaction temperature had the expected effect on chemoselectivity, and the desired product was isolated in comparatively better yields (Table 1, entry 12). In order to improve the solubility of the reagents in the reaction mixture, water was replaced by small amounts of DMSO, resulting in a quantitative conversion with complete selectivity toward diamine **2c** (Table 1, entry 13).

Analogous conditions as for **2b**, although doubling the reaction time, were then tested for the arylation of 2-ABA with the less reactive 2-fluorobenzonitrile (Table 1, entry 14). The best results were attained working at a slightly higher temperature, affording **2d** in good yield (Table 1, entry 15). A further increase in the reaction temperature, however, was unfavorable due to decomposition of the substrate (Table 1, entry 16).

Next, *N*-aryl-2-aminobenzylamines **2** were selectively acylated to yield compounds **3**, using acid chlorides or anhydrides. No *N*,*N*’-diacylation products were observed due to the low nucleophilicity of the secondary amino group, bearing a deactivating aromatic ring and all desired compounds were obtained with excellent yields (Table 2).

**Table 2:** Selective *N*-acylation of *N-*aryl-2-ABAs **2**.

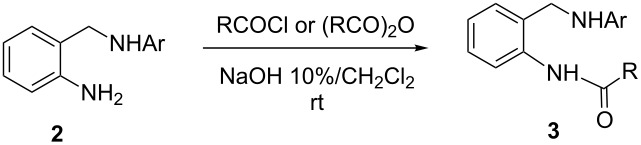				

Entry	**3**	Ar	R	Yield (%)^a^

1	**a**	2-NO_2_C_6_H_4_	CH_2_CH_3_	93
2	**b**	2-NO_2_C_6_H_4_	CH(CH_3_)_2_	92
3	**c**	2-NO_2_C_6_H_4_	C_6_H_5_	89
4	**d**	2-NO_2_C_6_H_4_	4-ClC_6_H_4_	quant.
5	**e**	2-NO_2_C_6_H_4_	CH_2_C_6_H_5_	quant.
6	**f**	2-NO_2_C_6_H_4_	4-ClC_6_H_4_CH_2_	quant.
7	**g**	4-NO_2_C_6_H_4_	CH_3_	92
8	**h**	4-NO_2_C_6_H_4_	CH_2_CH_3_	quant.
9	**i**	4-NO_2_C_6_H_4_	CH(CH_3_)_2_	95
10	**j**	4-NO_2_C_6_H_4_	C(CH_3_)_3_	86
11	**k**	4-NO_2_C_6_H_4_	C_6_H_5_	quant.
12	**l**	2-(3-NO_2_)C_5_H_3_N	CH_2_CH_3_	97
13	**m**	2-(3-NO_2_)C_5_H_3_N	CH(CH_3_)_2_	87
14	**n**	2-CNC_6_H_4_	CH_3_	93
15	**o**	2-CNC_6_H_4_	CH(CH_3_)_2_	81

^a^Yields correspond to pure compounds.

Then, the microwave-assisted cyclization reaction leading to 3,4-dihydroquinazolines **1** was initially tested with compound **3b** using PPE/Cl_3_CH as the dehydrating agent, on the basis of our previous results [41]. After disappearance of the starting material, the resulting yield of **1b** estimated from the crude product was disappointingly low (<20%). This behavior was unexpected and can be attributed to the low basicity of the dihydroquinazoline, which is not extracted in the acidic aqueous phase using the classical work-up procedure for PPE [41].

It had previously been reported that PPSE is a more efficient cyclization agent than PPE, allowing to perform some conversions that cannot be achieved by the latter [85,88]. Additionally, the classical work-up procedure of PPSE-promoted reactions involves basic conditions [85]. In fact, treatment of **3b** with PPSE/Cl_2_CH_2_ under microwave irradiation afforded the desired product **1b** with 90% yield (Table 3). In a control experiment, **3b** was reacted with PPE/Cl_3_CH and the product was isolated under basic conditions, affording compound **1b** in 81% yield after a cumbersome purification. Using this protocol, cyclization of precursor **3a** afforded 85% of the corresponding dihydroquinazoline **1a** vs 92% for the reaction with PPSE, which was thus chosen as the dehydrating agent. Employing the optimized reaction conditions, a series of novel *N-*aryl-3,4-dihydroquinazolines **1** was synthesized in high to excellent yields (Table 3). The reactions were performed, with the exception of **1j**, at 120 °C. Lower temperatures hindered the complete dissolution of the substrates thus increasing the reaction times. The irradiation times were individually adjusted according to the reactivity of the substrates.

**Table 3:** Synthesis of *N*-aryl-3,4-dihydroquinazolines **1**.

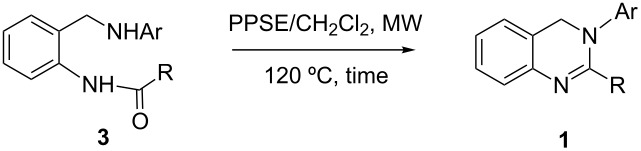						

Entry	**1**	Ar	R	Time (min)	Yield (%)^a^	Yield 2-ABA → **1** (%)

1	**a**	2-NO_2_C_6_H_4_	CH_2_CH_3_	15	92	82
2	**b**	2-NO_2_C_6_H_4_	CH(CH_3_)_2_	25	90	79
3	**c**	2-NO_2_C_6_H_4_	C_6_H_5_	90	87	74
4	**d**	2-NO_2_C_6_H_4_	4-ClC_6_H_4_	85	83	80
5	**e**	2-NO_2_C_6_H_4_	CH_2_C_6_H_5_	40	87	84
6	**f**	2-NO_2_C_6_H_4_	4-ClC_6_H_4_CH_2_	45	88	84
7	**g**	4-NO_2_C_6_H_4_	CH_3_	5	quant.	88
8	**h**	4-NO_2_C_6_H_4_	CH_2_CH_3_	12	93	89
9	**i**	4-NO_2_C_6_H_4_	CH(CH_3_)_2_	15	89	81
10^b^	**j**	4-NO_2_C_6_H_4_	C(CH_3_)_3_	90	87	72
11	**k**	4-NO_2_C_6_H_4_	C_6_H_5_	45	92	88
12	**l**	2-(3-NO_2_)C_5_H_3_N	CH_2_CH_3_	15	83	81
13	**m**	2-(3-NO_2_)C_5_H_3_N	CH(CH_3_)_2_	25	80	70
14	**n**	2-CNC_6_H_4_	CH_3_	5	91	58
15	**o**	2-CNC_6_H_4_	CH(CH_3_)_2_	30	77	42

^a^Yields correspond to pure compounds. ^b^The reaction was carried out at 150 °C.

Interestingly, the ^1^H NMR spectra of some *ortho-*substituted derivatives show nonequivalent hydrogens within the benzylic methylenes, which appear as AB spin systems (compounds **1a**,**b**,**e**,**f**) or as broadened signals (compounds **1c**,**d**,**n**,**o**). These spectral features would arise from restricted rotation around the *N*^3^*-*aryl bond, which entails the existence of conformational enantiomers [89].

Except for 2-cyano derivatives **1n**,**o**, the whole sequence afforded compounds **1** in remarkably high (>70%) to excellent (>80%) overall yields from 2-ABA in three steps (Table 3, last column).

Regarding the ring-closure step, Table 3 shows a clear dependence of the reaction times with steric hindrance of the amide group (R) in the substrate, displaying the order R = CH_3_ < CH_2_CH_3_ < CH(CH_3_)_2_ << C(CH_3_)_3_. In fact, the conversion of the *tert*-butyl derivative **3j** at 120 °C was slow, affording the desired product **1** in low yield (<10%) after 60 min. In this case, harsher reaction conditions were required for complete consumption of the starting material. In turn, the less reactive benzamides **3c**,**d**,**k** required relatively longer reaction times than alkaneamides to achieve complete conversion at 120 °C.

**Scheme 3:**
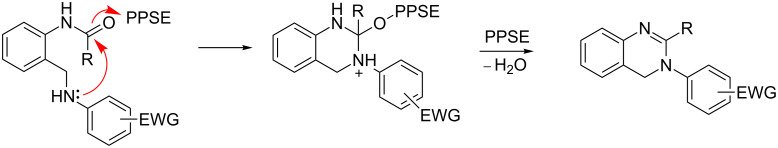
Addition–elimination mechanism for the heterocyclization.

On the other hand, the reaction times were almost insensitive to the electronic features of the *N-*aryl group. This was quite unexpected in the context of the accepted mechanism for this heterocyclization, which involves a nucleophilic attack of the arylamino group on the amide carbonyl followed by H_2_O elimination (Scheme 3) [90]. In such processes, PPSE would play a dual role, activating the amide towards the nucleophilic attack and acting as water scavenger. However, deactivated arylamino groups such as 2/4-nitrophenylanilines within compounds **3** are poor nucleophiles, and the *N-*2-(3-nitropyridyl) group is even less reactive. This is reflected by the fact that, as previously mentioned, compounds **3** do not react further with acylating agents to give the *N*,*N*’*-*diacylation products.

**Scheme 4:**
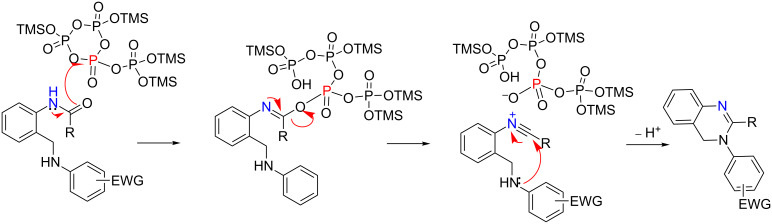
Proposed mechanism involving an intermediate nitrilium ion.

On the basis of some literature data, an alternative mechanism involving nitrilium ions as intermediates can be proposed (Scheme 4). Nitrilium ions [91] are known to mediate different organic reactions such as the Beckmann rearrangement and the Bischler–Napieralski, von Braun and Ritter reactions, among others [92–93]. Stable nitrilium salts can be generated by Lewis acid-promoted halide abstraction from imidoyl chlorides or by alkylation of nitriles. A nucleophilic attack on such species followed by cyclization provides access to a variety of heterocycles. Intramolecular reactions of in situ generated nitrilium ions leading to heterocyclic compounds have also been reported [91]. These transient intermediates have been characterized spectroscopically and, in some cases, were isolated from the reaction medium [94–97]. In relation to our research, nitrilium ions are also known to mediate the synthesis of acyclic amidines by reaction with primary and secondary amines [98]. On the other hand, it is known that *N-*unsubstituted amides react with PPSE affording nitriles [99]. In this context, the cyclodehydration of *N-*monosubstituted amides **3** could in principle involve an initial PPSE-mediated elimination generating a reactive nitrilium ion in situ (Scheme 4). This transient species is a powerful internal electrophile which would readily undergo intramolecular attack even by poor nucleophiles like the deactivated arylamino groups present in compounds **3**.

## Conclusion

Our synthetic approach represents the first method for the preparation of 2-substituted-3-aryl-3,4-dihydroquinazolines with EWGs in the aryl moiety. The sequence involves a minimum number of steps, is operationally simple and requires easily available and inexpensive reagents. A careful optimization of the individual steps allowed for the selective functionalization of 2-ABA, avoiding the use of protecting groups, and affording the desired heterocycles in good to excellent overall yields.

A suitable balance between reactivity and selectivity was achieved in the S_N_Ar reaction which, to our knowledge, is the first report on a successful uncatalyzed *N-*monoarylation of the unprotected diamine performed under equimolar conditions. These results are of particular relevance due to the wide use of the S_N_Ar reaction in synthetic organic chemistry.

The cyclization of functionalized 2-ABAs was performed efficiently under MW irradiation using PPSE as the dehydrating agent. This approach avoids the use of strong protic acids, which may be incompatible with sensitive substrates. Two alternative mechanisms can be proposed for the reaction. An initial elimination yielding a transient nitrilium ion intermediate followed by a nucleophilic attack seems more likely on the basis of literature data and our experimental observations.

## Supporting Information

File 1 Experimental procedures and characterization of new compounds.
