# Supplementary material for: Synthesis of dihydroquinazolines from 2-aminobenzylamine: N3-aryl derivatives with electron-withdrawing groups
Source: Beilstein J Org Chem. 2018 Sep 26;14:2510–9. doi: 10.3762/bjoc.14.227 (PMC6178284; doi:10.3762/bjoc.14.227)

Supporting Information

for

Synthesis of dihydroquinazolines from 2-aminobenzylamine: N^3-aryl derivatives with electron-withdrawing groups

Nadia Gruber, Jimena E. Díaz and Liliana R. Orelli*

Address: Universidad de Buenos Aires, CONICET, Departamento de Química Orgánica, Facultad de Farmacia y Bioquímica, Junín 956, (1113) Buenos Aires, Argentina

Email: Liliana R. Orelli - lorelli@ffyb.uba.ar

*Corresponding author

Experimental procedures and characterization of new compounds

1. General information S2
2. Representative procedures for syntheses S2
3. Characterization data for compounds **1**–**3** S3
4. Copies of ^{1}H and ^{13}C NMR spectra of compounds **1**–**3** S17

1. General information

Melting points were determined with a Büchi capillary apparatus and are uncorrected. ^{1}H and ^{13}C NMR spectra were recorded on a Bruker Bio Spin Avance III 600 MHz spectrometer, a Bruker Avance II 500 MHz spectrometer or a Bruker MSL 300 MHz spectrometer. Chemical shifts are reported in ppm (δ) relative to TMS as an internal standard. D_2O was employed to confirm exchangeable protons (ex). Splitting multiplicities are reported as singlet (s), broad signal (bs), doublet (d), double doublet (dd), doublet of doublets of doublets (ddd), triplet (t), triplet of doublets (td), quartet (q), heptet (h) and multiplet (m). HRMS (ESI) were performed with a Bruker MicroTOF-Q II spectrometer. Reagents, solvents and starting materials were purchased from standard sources and purified according to literature procedures.

2. Representative procedures for syntheses

General procedure for the synthesis of substituted *N*-aryl-3,4-dihydroquinazolines 1:

In a similar manner as described in [1], a mixture of the corresponding compound **3** (0.5 mmol) and a dichloromethane solution of PPSE (3 mL) was reacted in a microwave reactor (Monowave 300, Anton Paar) with stirring (600 rpm) at the indicated temperature and time (Table 3). After reaching rt, the resulting solution was treated with dichloromethane (15 mL) and an aqueous solution of NaOH (10%, 15 mL). The aqueous phase was extracted with dichloromethane (3 × 15 mL). The organic layers were pooled, washed with water (5 mL), dried over sodium sulfate and filtered. The solvent was removed in vacuo. The crude products were purified by column chromatography (silica gel 60, DCM/ethyl acetate).

General procedure for the synthesis of *N*-aryl-*N*'-acyl-2-aminobenzylamines 3:

In a similar manner as described in [1], the corresponding acyl chloride (1 mmol) or anhydride (3 mmol) was dissolved in dichloromethane (5 mL) and added to a flask containing a dichloromethane solution of the suitable compound **2** (1 mmol in 30 mL) and an aqueous solution of NaOH (10%, 10 mL). The mixture was vigorously shaken until disappearance of compound **2** was evidenced by TLC, after which the organic layer was separated. The aqueous solution was extracted with dichloromethane (2 × 10 mL). The organic layers were pooled, washed with water (5 mL), dried over anhydrous sodium sulfate and filtered. The solvent was removed in vacuo, and the residue purified by column chromatography (silica gel 60, DCM/ethyl acetate).

General procedure for the synthesis of *N*-aryl-2-aminobenzylamines 2:

2-ABA (1 mmol) was reacted with the corresponding aryl halide under the conditions indicated in Table 1. The mixture was treated with water (5 mL) and extracted with dichloromethane (3 × 10 mL). The organic layers were pooled, washed with water (5 mL), dried over sodium sulfate and filtered. The solvent was removed in vacuo. The crude products were purified by column chromatography (silica gel 60, DCM/ethyl acetate).

[1] Díaz J. E.; Ranieri, S.; Gruber, N.; Orelli, L. R. *Beilstein J. Org. Chem.* **2017**, *13*, 1470-1477.

3. Characterization data for compounds **1–3**

2-Substituted *N*-aryl-3,4-dihydroquinazolines 1

2-Ethyl-3-(2-nitrophenyl)-3,4-dihydroquinazoline (1a)

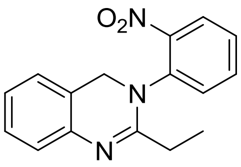

Orange solid (92% yield), mp: 100-103°C. ^{1}H NMR (500 MHz, $CDCl_3$): δ= 1.10 (t, *J*= 7.5 Hz, 3H), 2.03-2.21 (m, 2H), 4.63 (d, *J*= 13.3 Hz, 1H), 4.83 (d, *J*= 13.3 Hz, 1H), 6.83-6.86 (m, 1H), 7.01-7.05 (m, 1H), 7.16-7.23 (m, 2H), 7.42 (dd, *J*= 7.9, 1.4 Hz, 1H), 7.50-7.54 (m, 1H), 7.69 (td, *J*= 7.7, 1.5 Hz, 1H), 8.01 (dd, *J*= 8.2, 1.5 Hz, 1H). ^{13}C NMR (126 MHz, $CDCl_3$) δ= 11.5, 28.1, 52.2, 121.1, 124.3, 124.8, 125.1, 126.0, 128.6, 128.9, 131.1, 134.6, 137.8, 141.5, 146.9, 158.9. HRMS (ESI) m/z calcd for $C_{16}H_{16}N_3O_2$: 282.1237. Found: 282.1238.

2-Isopropyl-3-(2-nitrophenyl)-3,4-dihydroquinazoline (1b)

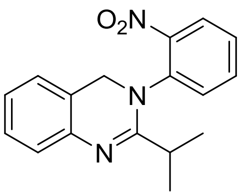

Orange solid (90% yield), mp: 140-142°C. ^{1}H NMR (300 MHz, $CDCl_3$): δ= 1.14 (d, *J*= 6.7 Hz, 3H), 1.21 (d, *J*= 6.7 Hz, 3H), 2.29 (h, *J*= 6.7 Hz, 1H), 4.57 (d, *J*= 13.3 Hz, 1H), 4.81 (d, *J*= 13.3 Hz, 1H), 6.81-6.88 (m, 1H), 7.00-7.08 (m, 1H), 7.19-7.25 (m, 2H), 7.36 (d, *J*= 7.9 Hz, 1H), 7.45-7.53 (m, 1H), 7.67 (td, *J*= 7.7, 1.6 Hz, 1H), 8.02 (dd, *J*= 8.1, 1.6 Hz, 1H). ^{13}C NMR (75 MHz, $CDCl_3$) δ= 20.9, 21.2, 32.2, 52.4, 121.4, 124.5, 124.8, 125.1, 126.0, 128.2, 128.5,

130.5, 134.4, 138.5, 141.9, 146.6, 162.8. HRMS (ESI) m/z calcd for $C_{17}H_{18}N_3O_2$: 296.1394. Found: 296.1392.

2-Phenyl-3-(2-nitrophenyl)-3,4-dihydroquinazoline (1c)

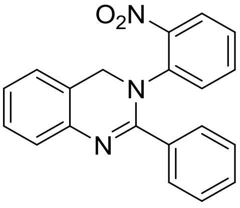

Orange solid (87% yield), mp: 157-159°C. ^{1}H NMR (600 MHz, $CDCl_3$): δ= 4.50 (bs, 1H), 5.02 (bs, 1H), 6.95-6.98 (m, 2H), 7.14-7.18 (m, 2H), 7.24 (t, *J*= 7.5 Hz, 2H), 7.28-7.33 (m, 3H), 7.43 (d, *J*= 7.7 Hz, 1H), 7.68-7.70 (m, 2H), 7.89 (dd, *J*= 8.3, 1.4 Hz, 1H). ^{13}C NMR (151 MHz, $CDCl_3$) δ= 51.9, 122.8, 125.0, 125.1, 125.6, 126.0, 126.2, 128.5, 128.7, 129.7, 130.4, 133.4, 135.4, 140.2, 142.4, 143.7, 155.3. HRMS (ESI) m/z calcd for $C_{20}H_{16}N_3O_2$: 330.1237. Found: 330.1246.

2-(4-Chlorophenyl)-3-(2-nitrophenyl)-3,4-dihydroquinazoline (1d)

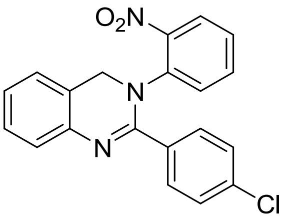

Orange solid (83% yield), mp: 201-203°C. ^{1}H NMR (300 MHz, $CDCl_3$): δ= 4.46 (bs, 1H), 4.50 (bs, 1H), 6.91 (dd, *J*= 8.1, 1.3 Hz, 1H), 6.96 (d, *J*= 7.4 Hz, 1H), 7.16 (td, *J*= 7.4, 1.3 Hz, 1H), 7.18-7.21 (m, 1H), 7.22 (d, *J*= 8.5 Hz, 2H), 7.30-7.34 (m, 2H), 7.40 (d, *J*= 7.4 Hz, 1H), 7.65 (d, *J*= 8.5 Hz, 1H), 7.91 (dd, *J*= 8.2, 1.6 Hz, 1H). ^{13}C NMR (151 MHz, $CDCl_3$) δ= 51.9, 122.7, 125.0, 125.0, 125.8, 126.1, 126.5, 128.77, 128.80, 129.6, 131.0, 133.6, 133.9, 136.5, 139.9, 142.2, 143.7, 154.1. HRMS (ESI) m/z calcd for $C_{20}H_{15}ClN_3O_2$: 364.0847. Found: 364.0850.

2-Benzyl-3-(2-nitrophenyl)-3,4-dihydroquinazoline (1e)

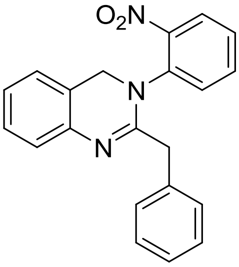

Orange oil (87% yield). ^{1}H NMR (600 MHz, $CDCl_3$): δ= 3.42 (d, *J*= 15.2 Hz, 1H), 3.69 (d, *J*= 15.2 Hz, 1H), 4.67 (d, *J*= 13.3 Hz, 1H), 4.84 (d, *J*= 13.3 Hz, 1H), 6.85-6.90 (m, 3H), 6.94-6.97 (m, 1H), 7.05-7.16 (m, 5H), 7.23-7.28 (m, 1H), 7.41-7.46 (m, 2H), 7.93-7.96 (m, 1H). ^{13}C NMR (151 MHz, $CDCl_3$) δ= 42.4, 52.6, 121.3, 124.6, 124.9, 125.5, 125.7, 126.7, 128.37, 128.41, 128.6, 128.9, 131.8, 134.1, 135.9, 137.2, 141.5, 146.3, 156.1. HRMS (ESI) m/z calcd for $C_{21}H_{18}N_3O_2$: 344.1394. Found: 344.1385.

2-(4-Chlorobenzyl)-3-(2-nitrophenyl)-3,4-dihydroquinazoline (1f)

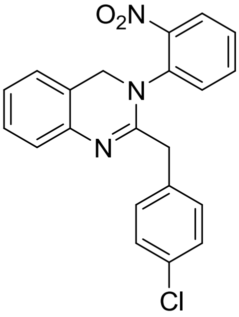

Orange solid (88% yield). ^{1}H NMR (300 MHz, $CDCl_3$): δ= 3.39 (d, *J*= 15.3 Hz, 1H), 3.65 (d, *J*= 15.3 Hz, 1H), 4.69 (d, *J*= 13.3 Hz, 1H), 4.86 (d, *J*= 13.3 Hz, 1H), 6.81-6.93 (m, 3H), 6.99-7.05 (m, 1H), 7.06-7.15 (m, 3H), 7.23-7.31 (m, 2H), 7.46-7.55 (m, 2H), 7.95-8.02 (m, 1H). ^{13}C NMR (75 MHz, $CDCl_3$) δ= 41.5, 52.5, 121.1, 124.6, 124.8, 125.5, 125.7, 128.4, 128.6, 129.0, 129.7, 131.6, 132.7, 134.2, 134.4, 137.0, 141.3, 146.7, 155.4. HRMS (ESI) m/z calcd for $C_{21}H_{17}ClN_3O_2$: 378.1004. Found: 378.1008.

2-Methyl-3-(4-nitrophenyl)-3,4-dihydroquinazoline (1g)

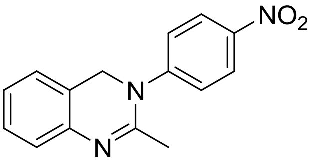

Yellow solid (100% yield), mp: 155-157°C. ^{1}H NMR (300 MHz, CDCl$_3$): δ= 2.17 (s, 3H), 4.85 (s, 2H), 6.92-7.02 (m, 1H), 7.08-7.16 (m, 1H), 7.18-7.23 (m, 1H), 7.25-7.30 (m, 1H), 7.32 (d, *J*= 8.9 Hz, 2H), 8.29 (d, *J*= 8.9 Hz, 2H). ^{13}C NMR (75 MHz, CDCl$_3$) δ= 23.2, 51.1, 121.7, 123.8, 124.7, 124.97, 125.04, 125.6, 128.9, 141.5, 144.9, 149.8, 154.5. HRMS (ESI) m/z calcd for $C_{15}H_{14}N_3O_2$: 268.1081. Found: 268.1072.

2-Ethyl-3-(4-nitrophenyl)-3,4-dihydroquinazoline (1h)

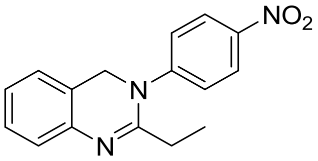

Yellow solid (93% yield), mp: 160-162°C. ^{1}H NMR (500 MHz, CDCl$_3$): δ= 1.18 (t, *J*= 7.5 Hz, 3H), 2.49 (c, *J*= 7.5 Hz, 2H), 4.84 (s, 2H), 6.99 (d, *J*= 7.4 Hz, 1H), 7.11-7.16 (m, 1H), 7.25-7.28 (m, 1H), 7.28-7.33 (m, 3H), 8.30 (d, *J*= 8.9 Hz, 2H). ^{13}C NMR (126 MHz, CDCl$_3$) δ= 12.1, 28.5, 51.5, 122.0, 124.1, 124.4, 124.9, 125.1, 125.7, 128.9, 141.7, 144.7, 150.0, 159.2. HRMS (ESI) m/z calcd for $C_{16}H_{16}N_3O_2$: 282.1237. Found: 282.1241.

2-Isopropyl-3-(4-nitrophenyl)-3,4-dihydroquinazoline (1i)

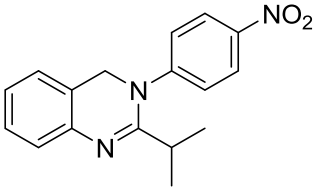

Yellow solid (89% yield), mp: 112-114°C. ^{1}H NMR (600 MHz, CDCl$_3$): δ= 1.26 (d, *J*= 6.7 Hz, 6H), 2.82 (h, *J*= 6.7 Hz, 2H), 4.82 (s, 2H), 6.98 (d, *J*= 7.4 Hz, 1H), 7.10-7.15 (m, 1H), 7.22 (d, *J*= 8.9 Hz, 1H), 7.28-7.31 (m, 2H), 8.27 (d, *J*= 8.9 Hz, 2H). ^{13}C NMR (126 MHz, CDCl$_3$) δ= 21.2, 31.6, 51.6, 122.2, 123.6, 124.1, 124.8, 125.0, 125.6, 128.6, 141.6, 144.1, 150.2, 162.8. HRMS (ESI) m/z calcd for $C_{17}H_{18}N_3O_2$: 296.1394. Found: 296.1390.

2-*Tert*-Butyl-3-(4-nitrophenyl)-3,4-dihydroquinazoline (1j)

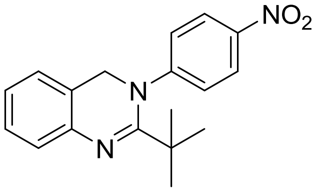

Yellow solid (87% yield), mp: 94-96°C. ^{1}H NMR (500 MHz, $CDCl_3$): δ= 1.27 (s, 9H), 4.60 (s, 2H), 6.94 (d, *J*= 7.5 Hz, 1H), 7.01 (d, bs, 2H), 7.12 (td, *J*= 7.5, 1.3 Hz, 1H), 7.29 (td, *J*= 7.5, 1.4 Hz, 1H), 7.32-7.39 (m, bs, 1H), 8.12 (d, *J*= 8.9 Hz, 2H). ^{13}C NMR (126 MHz, $CDCl_3$) δ= 30.7, 40.9, 54.5, 123.1, 124.0, 124.9, 125.1, 126.49, 126.54, 128.7, 132.2, 143.6, 153.1, 168.8. HRMS (ESI) m/z calcd for $C_{18}H_{20}N_3O_2$: 310.1550. Found: 310.1548.

2-Phenyl-3-(4-nitrophenyl)-3,4-dihydroquinazoline (1k)

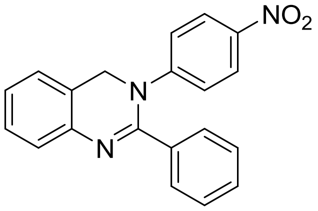

Yellow solid (92% yield), mp: 68-70°C. ^{1}H NMR (600 MHz, $CDCl_3$): δ= 5.06 (s, 2H), 6.98 (d, *J*= 9.1 Hz, 2H), 7.07 (d, *J*= 7.4 Hz, 1H), 7.20 (t, *J*= 7.4 Hz, 1H), 7.27-7.31 (m, 2H), 7.32-7.37 (m, 2H), 7.44-7.48 (m, 1H), 7.57-7.60 (m, 2H), 8.03 (d, *J*= 9.1 Hz, 2H). ^{13}C NMR (151 MHz, $CDCl_3$) δ= 51.1, 122.3, 123.0, 124.6, 124.8, 125.0, 126.5, 128.6, 128.9, 129.7, 130.5, 135.1, 142.1, 142.8, 150.8, 154.8. HRMS (ESI) m/z calcd for $C_{20}H_{16}N_3O_2$: 330.1237. Found: 330.1246.

2-Ethyl-3-(3-nitropyridin-2-yl)-3,4-dihydroquinazoline (1l)

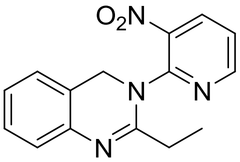

Orange oil (83% yield). ^{1}H NMR (300 MHz, $CDCl_3$): δ= 1.15 (t, *J*= 7.5 Hz, 3H), 2.43 (c, *J*= 7.5 Hz, 1H), 4.69 (s, 2H), 6.98 (d, *J*= 7.4 Hz, 1H), 7.07-7.15 (m, 1H), 7.25-7.33 (m, 3H), 8.38 (dd, *J*= 8.1, 1.6 Hz, 1H), 8.64 (dd, *J*= 4.6, 1.8 Hz, 1H). ^{13}C NMR (75 MHz, $CDCl_3$) δ= 11.8, 28.3, 49.9, 100.1, 120.1, 123.5, 124.6, 124.9, 125.9, 128.7, 135.0, 141.7, 149.2, 152.6, 158.7. HRMS (ESI) m/z calcd for $C_{15}H_{15}N_4O_2$: 283.1190. Found: 283.1185.

2-Isopropyl-3-(3-nitropyridin-2-yl)-3,4-dihydroquinazoline (1m)

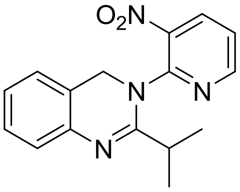

Orange oil (80% yield). ^{1}H NMR (600 MHz, $CDCl_3$): δ= 1.25 (d, *J*= 6.9 Hz, 6H), 2.62 (h, *J*= 6.9 Hz, 1H), 4.69 (s, 2H), 7.00 (d, *J*= 7.7 Hz, 1H), 7.12 (td, *J*= 7.3, 1.2 Hz, 1H), 7.24 (dd, *J*= 8.1, 4.4 Hz, 1H), 7.28-7.31 (m, 1H), 7.36 (d, *J*= 7.3 Hz, 1H), 8.37 (dd, *J*= 8.1, 1.6 Hz, 1H), 8.64 (dd, *J*= 4.4, 1.6 Hz, 1H). ^{13}C NMR (151 MHz, $CDCl_3$) δ= 20.7, 32.5, 50.1, 119.4, 123.9, 124.7, 124.8, 125.8, 128.6, 134.9, 138.4, 141.7, 149.4, 152.6, 162.5. HRMS (ESI) m/z calcd for $C_{16}H_{17}N_4O_2$: 297.1346. Found: 297.1353.

2-(2-Methylquinazolin-3(4*H*)-yl)benzonitrile (1n)

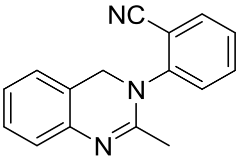

White solid (91% yield), mp: 159-161°C. ^{1}H NMR (600 MHz, $CDCl_3$): δ= 2.00 (s, 3H), 4.90 (bs, 2H), 6.90 (d, J= 7.2 Hz, 1H), 7.08 (td, *J*= 7.2, 1.7 Hz, 1H), 7.19-7.25 (m, 2H), 7.45 (d, *J*= 7.8 Hz, 1H), 7.49 (td, *J*= 7.8, 1.1 Hz, 1H), 7.71 (td, *J*= 7.8, 1.6 Hz, 1H), 7.78 (dd, *J*= 7.8, 1.6 Hz, 1H). ^{13}C NMR (151 MHz, $CDCl_3$) δ= 22.5, 52.2, 112.4, 114.1, 116.1, 120.9, 124.0, 125.0, 125.6, 127.9, 128.5, 128.9, 134.4, 134.7, 146.9, 155.0 HRMS (ESI) m/z calcd for $C_{16}H_{14}N_3$: 248.1182. Found: 248.1174.

2-(2-Isopropylquinazolin-3(4*H*)-yl)benzonitrile (1o)

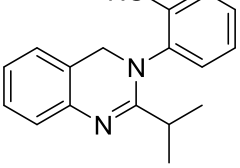

White solid (77% yield), mp: 122-124°C. ^{1}H NMR (600 MHz, $CDCl_3$): δ= 1.11-1.35 (m, 6H), 2.41 (h, *J*= 6.9 Hz, 1H), 4.54-5.21 (bs, 2H), 6.91 (d, *J*= 7.3 Hz, 1H), 7.06-7.10 (m, 1H), 7.24-7.27 (m, 2H), 7.30 (d, *J*= 8.1 Hz, 1H), 7.42 (t, *J*= 7.7 Hz, 1H), 7.66 (td, *J*= 7.7, 1.2 Hz, 1H), 7.75 (dd, *J*= 7.7, 1.2 Hz, 1H). ^{13}C NMR (151 MHz, $CDCl_3$) δ= 21.0, 32.0, 52.5, 111.6, 116.6,

121.8, 124.5, 124.8, 125.2, 127.3, 128.2, 128.5, 134.18, 134.21, 142.0, 148.0, 162.7. HRMS (ESI) m/z calcd for $C_{18}H_{18}N_3$: 276.1495. Found: 276.1490.

N-aryl-2-aminobenzylamines 2

***N*-(2-Aminobenzyl)-2-nitroaniline (2a)**

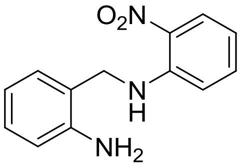

Orange solid (96% yield), mp: 89-91°C. ^{1}H NMR (500 MHz, $CDCl_3$): δ= 3.82 (bs, ex, 2H), 4.40 (d, *J*= 4.9 Hz, 2H), 6.70-6.81 (m, 3H), 6.94 (dd, *J*= 8.6, 0.9 Hz, 1H), 7.15-7.19 (m, 2H), 7.46 (ddd, *J*= 8.6, 7.0, 1.6 Hz, 1H), 8.02 (bs, ex, 1H), 8.20 (dd, *J*= 8.6, 1.6 Hz, 1H). ^{13}C NMR (126 MHz, $CDCl_3$) δ= 45.0, 114.4, 116.2, 116.6, 119.1, 121.2, 127.0, 129.4, 129.5, 132.7, 136.4, 144.9, 145.3. HRMS (ESI) m/z calcd for $C_{13}H_{14}N_3O_2$: 244.1081. Found: 244.1077.

***N*-(2-Aminobenzyl)-4-nitroaniline (2b)**

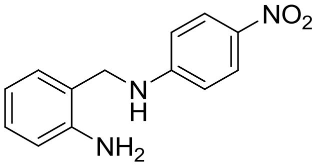

Yellow solid (96% yield), mp: 160-161°C. ^{1}H NMR (500 MHz, $CDCl_3$): δ= 4.31 (s, 2H), 4.53 (bs, ex, 2H), 6.64 (d, *J*= 9.0 Hz, 2H), 6.77-6.84 (m, 2H), 7.16-7.22 (m, 2H), 8.10 (d, *J*= 9.0 Hz, 2H). ^{13}C NMR (126 MHz, $CDCl_3$) δ= 45.8, 111.8, 116.6, 119.2, 121.2, 126.5, 129.7, 130.1, 138.9, 145.0, 153.4. HRMS (ESI) m/z calcd for $C_{13}H_{14}N_3O_2$: 244.1081.Found: 244.1090.

***N*-(2-Aminobenzyl)- 2-amine-3-nitropyridine (2c)**

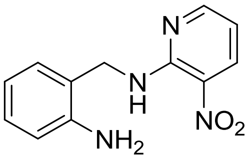

Orange solid (100% yield), mp: 104-106°C. ^{1}H NMR (500 MHz, $CDCl_3$): δ= 4.24 (bs, ex, 2H), 4.78 (d, *J*= 5.8 Hz, 2H), 6.66-6.72 (m, 2H), 6.78 (t, *J*= 7.5 Hz, 1H), 7.21 (d, *J*= 7.5 Hz, 1H), 8.39-8.46 (m, 3H). ^{13}C NMR (126 MHz, $CDCl_3$) δ= 42.2, 112.1, 116.2, 118.6, 122.3, 128.4,

129.3, 130.7, 135.8, 145.5, 152.4, 155.6. HRMS (ESI) m/z calcd for $C_{12}H_{13}N_4O_2$: 245.1033. Found: 245.1040.

2-(2-Aminobenzylamino)benzonitrile (2d)

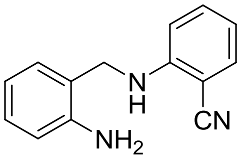

White solid (68% yield), mp: 118-120°C. ^{1}H NMR (500 MHz, $CDCl_3$): δ= 3.74 (bs, ex, 2H), 4.29 (s, 2H), 6.74-6.82 (m, 4H), 7.15-7.20 (m, 2H), 7.40-7.45 (m, 2H). ^{13}C NMR (126 MHz, $CDCl_3$) δ= 45.9, 96.8, 111.6, 116.6, 117.6, 117.8, 119.1, 121.5, 129.4, 129.9, 133.0, 134.5, 145.2, 150.4. HRMS (ESI) m/z calcd for $C_{14}H_{14}N_3$: 224.1182. Found: 224.1186.

***N*-aryl-*N*'-acyl-2-aminobenzylamines 3**

***N*-(2-((2-Nitrophenylamino)methyl)phenyl)propionamide (3a)**

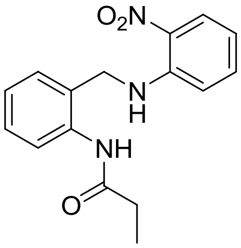

Orange solid (93% yield), mp: 141-143°C. ^{1}H NMR (500 MHz, $CDCl_3$): δ= 1.20 (t, *J*= 7.6 Hz, 3H), 2.38 (c, *J*= 7.6 Hz, 2H), 4.49 (s, 2H), 6.72 (ddd, *J*= 8.5, 7.0, 1.2 Hz, 1H), 6.88 (d, *J*= 8.5 Hz, 1H), 7.17-7.22 (m, 1H), 7.29-7.36 (m, 2H), 7.42 (ddd, *J*= 8.5, 7.0, 1.6 Hz, 1H), 7.53 (bs, ex, 1H), 7.60 (d, *J*= 7.8 Hz, 1H), 8.13 (bs, ex, 1H), 8.19 (dd, *J*= 8.5, 1.6 Hz, 1H). ^{13}C NMR (126 MHz, $CDCl_3$) δ= 9.9, 30.5, 45.0, 114.5, 116.6, 125.4, 126.4, 127.0, 128.79, 128.84, 130.4, 132.8, 135.6, 136.6, 145.1, 172.6. HRMS (ESI) m/z calcd for $C_{16}H_{18}N_3O_3$: 300.1343. Found: 300.1347.

***N*-(2-((2-Nitrophenylamino)methyl)phenyl)isobutyramide (3b)**

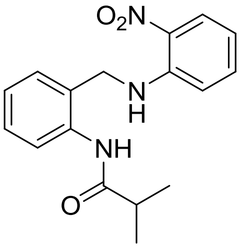

Orange solid (92% yield), mp: 170-172°C. ^{1}H NMR (300 MHz, $CDCl_3$): δ= 1.21 (d, *J*= 6.9 Hz, 6H), 2.52 (h, *J*= 6.9 Hz, 1H), 4.49 (d, *J*= 5.0 Hz, 2H), 6.74 (ddd, *J*= 8.6, 7.0, 1.2 Hz, 1H), 6.91 (d, *J*= 8.6 Hz, 1H), 7.15-7.24 (m, 1H), 7.30-7.38 (m, 2H), 7.45 (ddd, *J*= 8.6, 7.0, 1.6 Hz, 1H), 7.51 (bs, ex, 1H), 7.66 (d, *J*= 7.8 Hz, 1H), 8.10 (bs, ex, 1H), 8.21 (dd, *J*= 8.6, 1.6 Hz, 1H). ^{13}C NMR (75 MHz, $CDCl_3$) δ= 19.7, 36.5, 45.1, 114.5, 116.7, 125.2, 126.3, 127.0, 128.9, 129.0, 130.1, 133.0, 135.8, 136.6, 145.1, 175.7. HRMS (ESI) m/z calcd for $C_{17}H_{20}N_3O_3$: 314.1499. Found: 314.1489.

***N*-(2-((2-Nitrophenylamino)methyl)phenyl)benzamide (3c)**

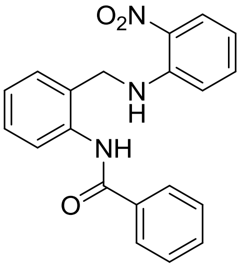

Orange solid (89% yield), mp: 134-136°C. ^{1}H NMR (600 MHz, $CDCl_3$): δ= 4.55 (d, *J*= 5.7 Hz, 2H), 6.71-6.74 (m, 1H), 6.91 (d, *J*= 8.5 Hz, 1H), 7.22-7.25 (m, 1H), 7.35-7.41 (m, 5H), 7.49-7.52 (m, 1H), 7.76 (d, *J*= 8.1 Hz, 1H), 7.80 (d, *J*= 7.7 Hz, 2H), 8.13 (t, bs, 1H), 8.19 (dd, *J*= 8.5, 1.6 Hz, 1H), 8.48 (bs, ex, 1H). ^{13}C NMR (151 MHz, $CDCl_3$) δ= 45.2, 114.7, 116.8, 125.1, 126.4, 127.0, 127.2, 128.86, 128.91, 128.95, 130.3, 132.2, 133.0, 134.2, 136.0, 136.6, 145.1, 166.1. HRMS (ESI) m/z calcd for $C_{20}H_{18}N_3O_3$: 348.1343. Found: 348.1339.

4-Chloro-*N*-(2-((2-nitrophenylamino)methyl)phenyl)benzamide (3d)

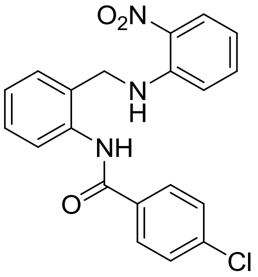

Orange solid (100% yield). ^{1}H NMR (500 MHz, $CDCl_3$): δ= 4.56 (d, *J*= 4.6 Hz, 2H), 6.75-6.80 (m, 1H), 6.95 (d, *J*= 8.6 Hz, 1H), 7.23-7.27 (m, 1H), 7.35-7.42 (m, 4H), 7.42-7.46 (m, 1H), 7.72 (d, *J*= 8.5 Hz, 2H), 7.83 (d, *J*= 8.3 Hz, 1H), 8.08 (bs, ex, 1H), 8.21 (d, J= 8.6 Hz, 1H), 8.39 (bs, ex, 1H). ^{13}C NMR (126 MHz, $CDCl_3$) δ= 29.9, 114.7, 117.2, 124.9, 126.4, 127.1, 128.6, 129.20, 129.23, 129.3, 129.7, 132.7, 133.3, 136.0, 136.7, 138.6, 144.9, 164.9. HRMS (ESI) m/z calcd for $C_{20}H_{17}ClN_3O_3$: 382.0953. Found: 382.0948.

***N*-(2-((2-Nitrophenylamino)methyl)phenyl)-2-phenylacetamide (3e)**

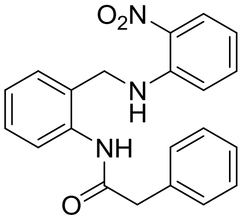

Orange solid (100% yield), mp: 172-175°C. ^{1}H NMR (500 MHz, $CDCl_3$): δ= 3.73 (s, 2H), 4.12 (d, *J*= 4.9 Hz, 2H), 6.72 (d, *J*= 8.5 Hz, 1H), 6.80 (ddd, *J*= 8.5, 6.8, 1.2 Hz, 1H), 6.93-6.97 (m, 1H), 7.02-7.07 (m, 2H), 7.12-7.19 (m, 3H), 7.23-7.27 (m, 1H), 7.36 (td, *J*=7.8, 1.6 Hz, 1H), 7.41 (bs, ex, 1H), 7.47 (ddd, *J*= 8.5, 6.8, 1.6 Hz, 1H), 7.50 (bs, ex, 1H), 7.89 (d, *J*=7.8 Hz, 1H), 8.21 (dd, *J*= 8.5, 1.6 Hz, 1H). ^{13}C NMR (126 MHz, $CDCl_3$) δ= 44.9, 45.2, 114.5, 116.7, 124.1, 125.8, 126.9, 127.6, 128.0, 128.7, 129.1, 129.3, 129.5, 129.6, 134.0, 136.1, 136.4, 144.7, 168.8. HRMS (ESI) m/z calcd for $C_{21}H_{20}N_3O_3$: 362.1499. Found: 362.1495.

2-(4-Chlorophenyl)-*N*-(2-((2-nitrophenylamino)methyl)phenyl)acetamide (3f)

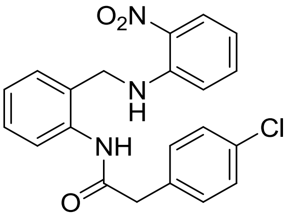

Orange solid (100% yield), mp: 187-188°C. ^{1}H NMR (600 MHz, $CDCl_3$): δ= 3.68 (s, 2H), 4.17 (s, 2H), 6.73 (d, *J*= 8.5 Hz, 1H), 6.79-6.83 (m, 1H), 7.04 (d, *J*= 8.0 Hz, 2H), 7.12 (d, *J*= 8.0 Hz, 2H), 7.08-7.15 (m, 1H), 7.26-7.29 (m, 1H), 7.34-7.37 (m, 1H), 7.49-7.52 (m, 1H), 7.59 (bs, ex, 1H), 7.83 (d, *J*= 8.2 Hz, 1H), 8.23 (d, J= 8.6 Hz, 1H). ^{13}C NMR (151 MHz, $CDCl_3$) δ= 44.1, 45.4, 114.3, 117.0, 124.3, 126.1, 127.2, 128.4, 128.9, 129.3, 129.4, 129.7, 130.8, 130.9, 132.6, 135.9, 136.6, 144.6, 169.2. HRMS (ESI) m/z calcd for $C_{21}H_{19}ClN_3O_3$: 396.1109. Found: 396.1112.

_N_-(2-((4-Nitrophenylamino)methyl)phenyl)acetamide (3g)

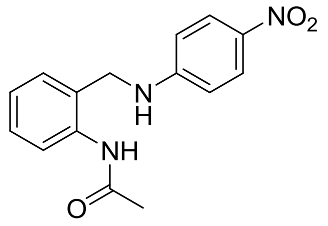

Yellow solid (92% yield), mp: 188-190°C. ^{1}H NMR (600 MHz, DMSO-*d*6): δ= 2.07 (s, 3H), 4.37 (d, *J*= 6.0 Hz, 2H), 6.60 (d, *J*= 9.3 Hz, 2H), 7.13-7.17 (m, 1H), 7.21-7.26 (m, 2H), 7.35 (d, *J*= 7.8 Hz, 1H), 7.74 (t, *J*= 6.0 Hz, 1H), 7.98 (d, *J*= 9.3 Hz, 2H), 9.53 (bs, 1H). ^{13}C NMR (151 MHz, DMSO-*d*6) δ= 23.2, 42.4, 111.1, 125.6, 125.9, 126.2, 126.9, 127.2, 132.5, 135.8, 136.0, 154.5, 168.6. HRMS (ESI) m/z calcd for $C_{15}H_{16}N_3O_3$: 286.1186. Found: 286.1190.

_N_-(2-((4-Nitrophenylamino)methyl)phenyl)propionamide (3h)

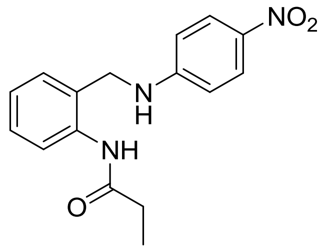

Yellow solid (100% yield), mp: 202-204°C. ^{1}H NMR (500 MHz, DMSO-*d*6): δ= 1.09 (t, *J*= 7.6 Hz, 3H), 2.35 (c, *J*= 7.6 Hz, 2H), 4.36 (d, *J*= 6.0 Hz, 2H), 6.60 (d, *J*= 9.3 Hz, 2H), 7.13-7.17 (m, 1H), 7.21-7.27 (m, 2H), 7.35 (d, *J*= 7.7 Hz, 1H), 7.74 (t, *J*= 6.0 Hz, 1H), 7.98 (d, *J*= 9.3 Hz, 2H), 9.46 (bs, 1H). ^{13}C NMR (126 MHz, DMSO-*d*6) δ= 9.9, 28.9, 42.4, 111.2, 125.5, 125.9, 126.2, 127.0, 127.2, 132.5, 135.8, 136.0, 154.5, 172.3. HRMS (ESI) m/z calcd for $C_{16}H_{18}N_3O_3$: 300.1343. Found: 300.1334.

N-(2-((4-Nitrophenylamino)methyl)phenyl)isobutyramide (3i)

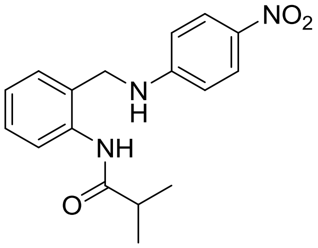

Yellow solid (95% yield), mp: 212-213°C. ^{1}H NMR (500 MHz, DMSO-*d*6): δ= 1.11 (d, *J*= 6.8 Hz, 6H), 2.64 (h, *J*= 6.8 Hz, 1H), 4.34 (d, *J*= 6.0 Hz, 2H), 6.60 (d, *J*= 9.3 Hz, 2H), 7.14-7.18 (m, 1H), 7.21-7.28 (m, 2H), 7.32 (d, *J*= 7.8 Hz, 1H), 7.73 (t, *J*= 6.0 Hz, 1H), 7.98 (d, *J*= 9.3 Hz, 2H), 9.46 (bs, 1H). ^{13}C NMR (126 MHz, DMSO-*d*6) δ= 19.6, 34.5, 42.4, 111.1, 125.7, 126.18, 126.24, 127.0, 127.3, 132.8, 135.8, 136.1, 154.5, 175.5. HRMS (ESI) m/z calcd for $C_{17}H_{20}N_3O_3$: 314.1499. Found: 314.1503.

N-(2-((4-Nitrophenylamino)methyl)phenyl)pivalamide (3j)

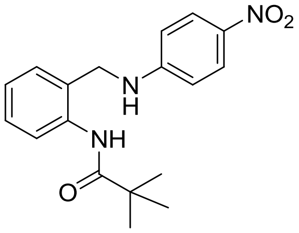

Yellow solid (86% yield), mp: 166-168°C. ^{1}H NMR (300 MHz, DMSO-*d*6): δ= 1.22 (s, 9H), 4.31 (d, *J*= 5.9 Hz, 2H), 6.60 (d, *J*= 8.9 Hz, 2H), 7.12-7.32 (m, 4H), 7.73 (t, *J*= 5.9 Hz, 1H), 7.98 (d, *J*= 8.9 Hz, 2H), 9.10 (bs, 1H). ^{13}C NMR (75 MHz, DMSO-*d*6) δ= 27.4, 38.8, 42.5, 111.2, 126.0, 126.2, 126.95, 127.02, 127.3, 133.7, 136.2, 136.2, 154.5, 176.9. HRMS (ESI) m/z calcd for $C_{18}H_{22}N_3O_3$: 328.1656. Found: 328.1651.

N-(2-((4-Nitrophenylamino)methyl)phenyl)benzamide (3k)

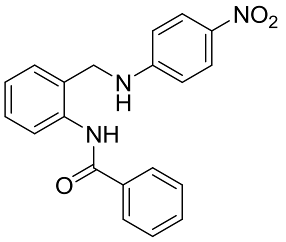

Yellow solid (100% yield), mp: 167-170°C. ^{1}H NMR (500 MHz, DMSO-*d*6): δ= 4.45 (d, *J*= 6.0 Hz, 2H), 6.63 (d, *J*= 8.8 Hz, 2H), 7.22-7.27 (m, 1H), 7.28-7.34 (m, 2H), 7.42 (d, *J*= 7.5 Hz,

1H), 7.49-7.55 (m, 2H), 7.56-7.61 (m, 1H), 7.79 (t, *J*= 6.0 Hz, 1H), 7.94-8.01 (m, 4H), 10.10 (bs, 1H). ^{13}C NMR (126 MHz, DMSO-*d*6) δ= 42.6, 111.2, 126.2, 126.3, 126.9, 127.0, 1274.4, 127.7, 128.5, 131.7, 133.9, 134.3, 135.8, 136.1, 154.5, 165.8. HRMS (ESI) m/z calcd for $C_{20}H_{18}N_3O_3$: 348.1343. Found: 348.1351.

***N*-(2-((3-Nitropyridin-2-ylamino)methyl)phenyl)propionamide (3l)**

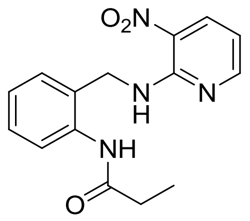

Orange solid (97% yield), mp: 169-171°C. ^{1}H NMR (500 MHz, $CDCl_3$): δ= 1.30 (t, *J*= 7.6 Hz, 3H), 2.48 (c, *J*= 7.6 Hz, 2H), 4.76 (d, *J*= 6.6 Hz, 2H), 6.75 (dd, *J*= 8.3, 4.7 Hz, 1H), 7.11-7.15 (m, 1H), 7.30-7.34 (m, 1H), 7.37 (d, *J*= 7.6 Hz, 1H), 8.00 (d, *J*= 8.1 Hz, 1H), 8.45 (dd, *J*= 4.7, 1.8 Hz, 1H), 8.49 (dd, *J*= 8.3, 1.8 Hz, 1H), 8.72 (bs, ex, 1H), 9.74 (bs, ex, 1H). ^{13}C NMR (126 MHz, $CDCl_3$) δ= 10.0, 31.0, 41.6, 112.5, 123.7, 124.9, 128.4, 129.0, 129.0, 130.8, 136.5, 136.6, 152.1, 154.7, 172.4. HRMS (ESI) m/z calcd for $C_{15}H_{17}N_4O_3$: 301.1295. Found: 301.1285.

***N*-(2-((3-Nitropyridin-2-ylamino)methyl)phenyl)isobutyramide (3m)**

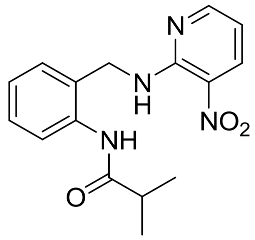

Orange solid (87% yield), mp: 168-170°C. ^{1}H NMR (600 MHz, $CDCl_3$): δ= 1.31 (d, *J*= 6.9 Hz, 6H), 2.62 (h, *J*= 6.9 Hz, 2H), 4.76 (d, *J*= 6.9 Hz, 2H), 6.76 (dd, *J*= 8.3, 4.6 Hz, 1H), 7.12-7.15 (m, 1H), 7.30-7.33 (m, 1H), 7.38 (d, *J*= 7.7 Hz, 1H), 7.98 (d, *J*= 8.1 Hz, 1H), 8.44 (dd, *J*= 4.6, 1.6 Hz, 1H), 8.49 (dd, *J*= 8.3, 1.6 Hz, 1H), 8.73 (t, bs, 1H), 9.63 (bs, ex, 1H). ^{13}C NMR (151 MHz, $CDCl_3$) δ= 19.9, 36.9, 41.6, 112.5, 124.0, 125.0, 128.6, 129.0, 129.1, 130.8, 136.5, 145.1, 152.2, 154.8, 175.6. HRMS (ESI) m/z calcd for $C_{16}H_{19}N_4O_3$: 315.1452. Found: 315.1455.

***N*-(2-((2-Cyanophenylamino)methyl)phenyl)acetamide (3n)**

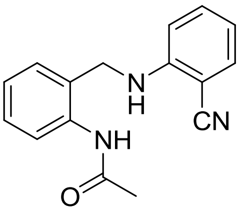

White solid (93% yield), mp: 170-172°C. ^{1}H NMR (500 MHz, $CDCl_3$): δ= 2.15 (s, 3H), 4.39 (s, 2H), 6.77-6.82 (m, 2H), 7.15-7.20 (m, 1H), 7.30-7.36 (m, 2H), 7.38-7.43 (m, 1H), 7.45 (d, *J*= 7.6 Hz, 1H), 7.74 (d, *J*= 8.0 Hz, 1H), 7.84 (bs, ex, 1H). ^{13}C NMR (126 MHz, $CDCl_3$) δ= 24.4, 46.1, 97.4, 112.2, 117.7, 118.4, 124.7, 125.9, 129.0, 129.2, 129.3, 133.1, 134.6, 136.3, 150.0, 168.9. HRMS (ESI) m/z calcd for $C_{16}H_{16}N_3O$: 266.1288. Found: 266.1292.

***N*-(2-((2-Cyanophenylamino)methyl)phenyl)isobutyramide (3o)**

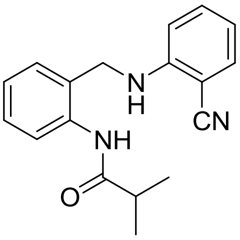

White solid (80% yield), mp: 193-195°C. ^{1}H NMR (600 MHz, $CDCl_3$): δ= 1.21 (d, *J*= 6.9 Hz, 6H), 2.51 (h, *J*= 6.9 Hz, 1H), 4.37 (d, *J*= 5.3 Hz, 2H), 4.70 (t, bs, 1H), 6.81-6.83 (m, 2H), 7.16 (t, *J*= 7.5 Hz, 1H), 7.32 (d, *J*= 7.3 Hz, 1H), 7.34-7.37 (m, 1H), 7.43-7.45 (m, 1H), 7.47 (dd, *J*= 7.8, 1.6 Hz, 1H), 7.87 (d, *J*= 8.1 Hz, 1H), 7.94 (bs, ex, 1H). ^{13}C NMR (151 MHz, $CDCl_3$) δ= 19.7, 36.7, 46.4, 97.7, 112.2, 117.6, 118.6, 124.2, 125.5, 128.4, 129.2, 129.4, 133.2, 134.7, 136.7, 149.9, 175.6. HRMS (ESI) m/z calcd for $C_{18}H_{20}N_3O$: 294,1601. Found: 294.1608.

4. Copies of ^{1}H and ^{13}C NMR Spectra of compounds **1-3**

^{1}H NMR (500 MHz, $CDCl_3$) spectrum of compound **1a**

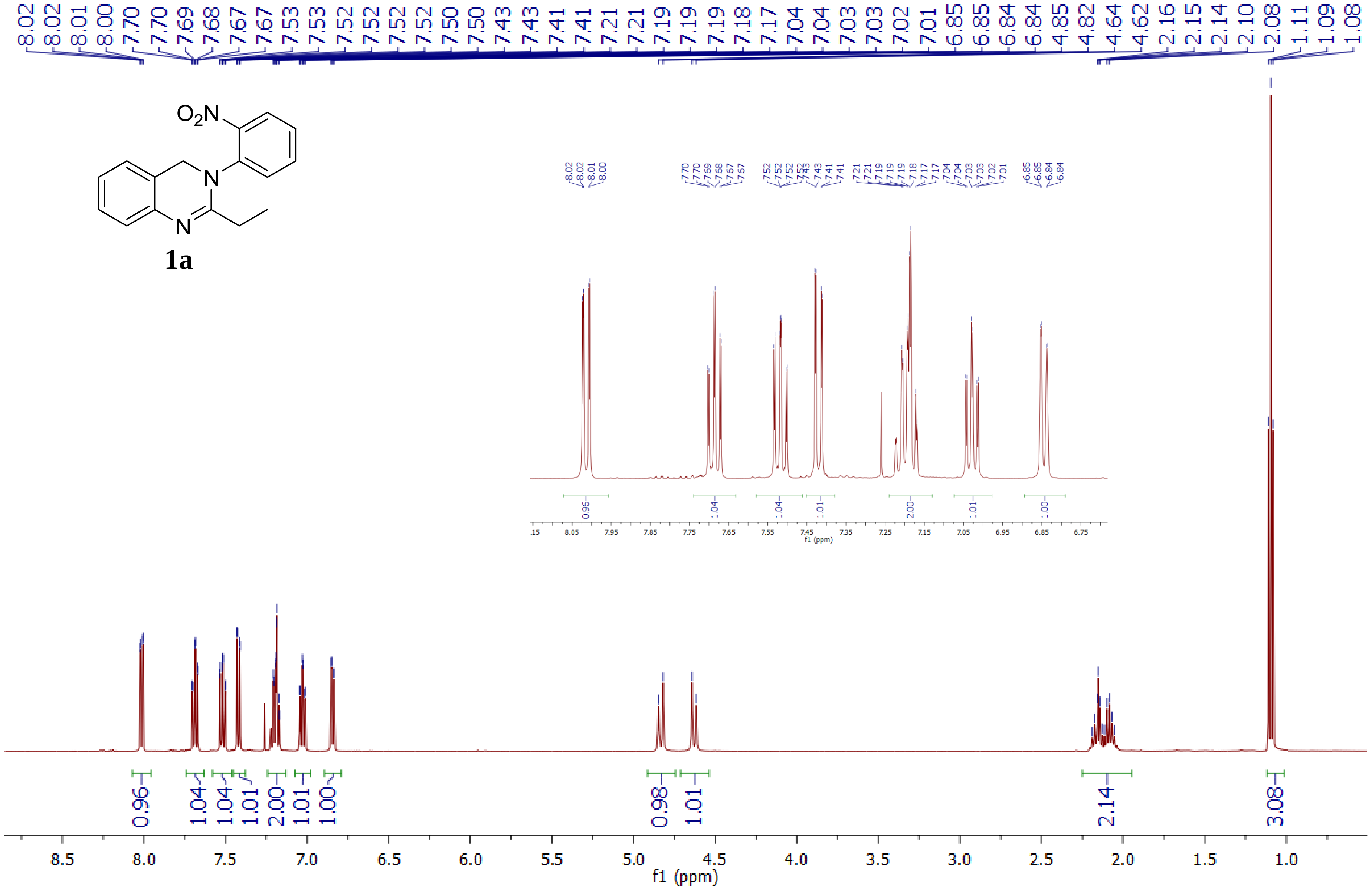

^{13}C NMR (126 MHz, $CDCl_3$) spectrum of compound **1a**

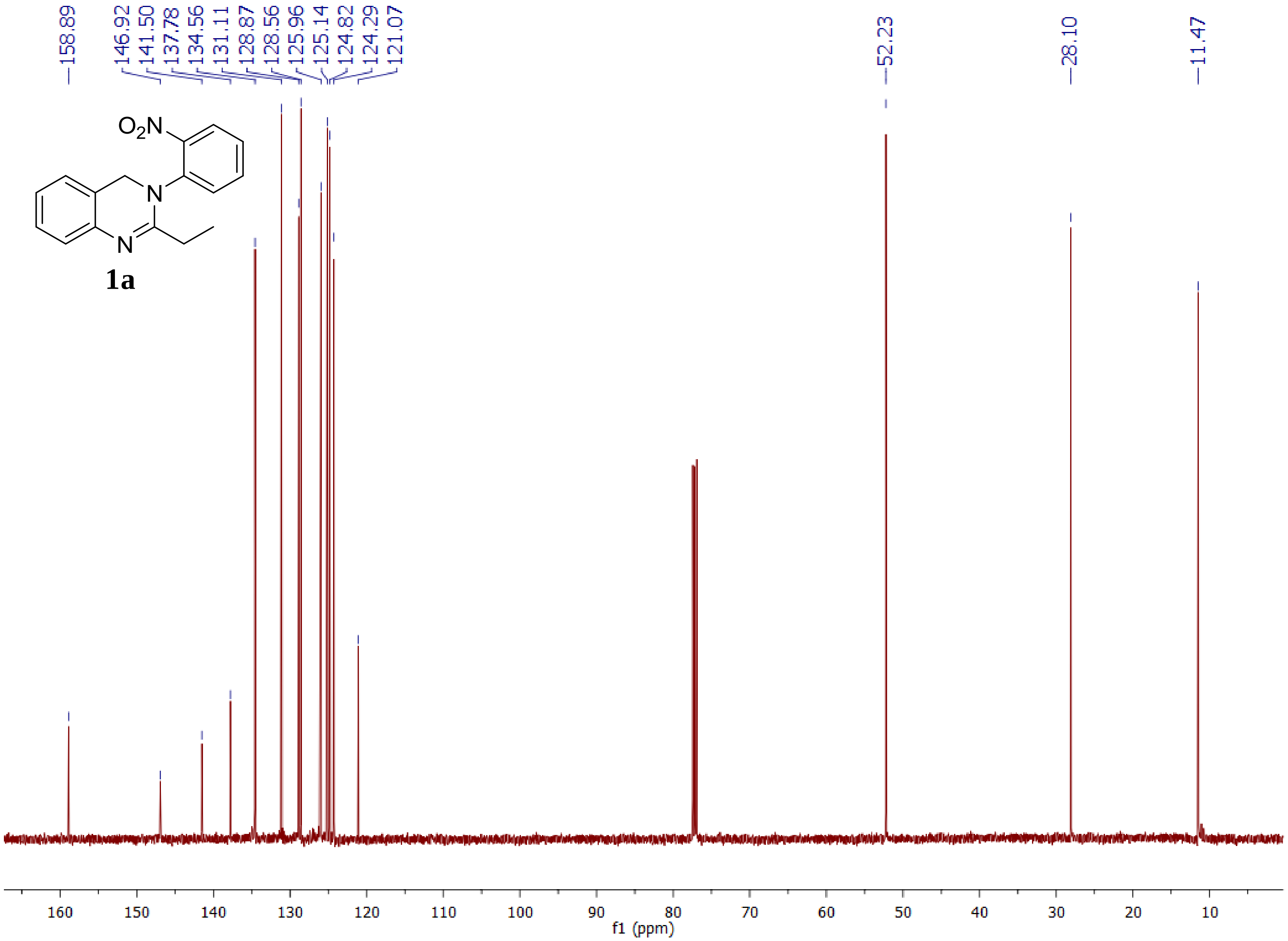

^{1}H NMR (300 MHz, $CDCl_3$) spectrum of compound **1b**

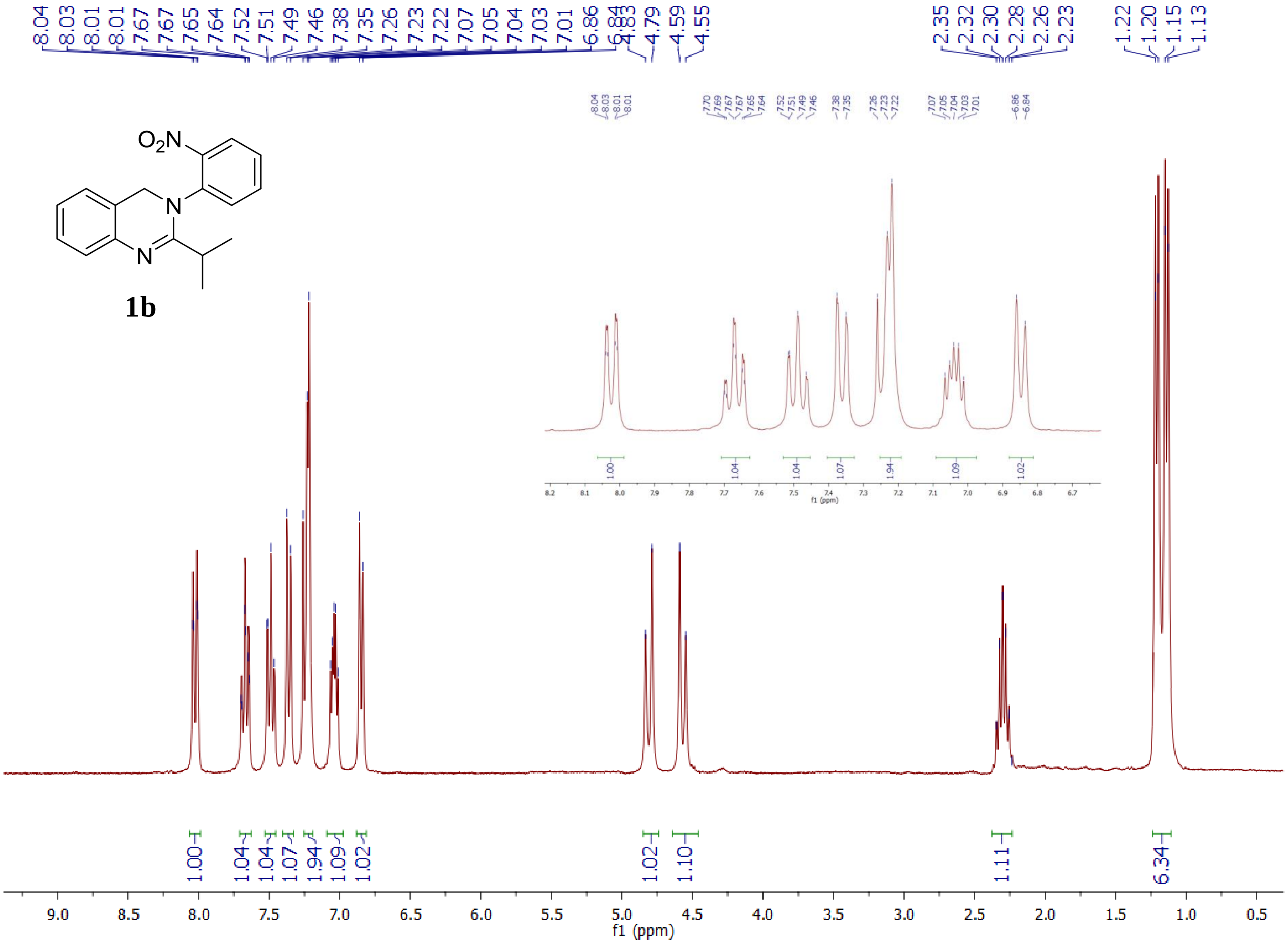

^{13}C NMR (75 MHz, $CDCl_3$) spectrum of compound **1b**

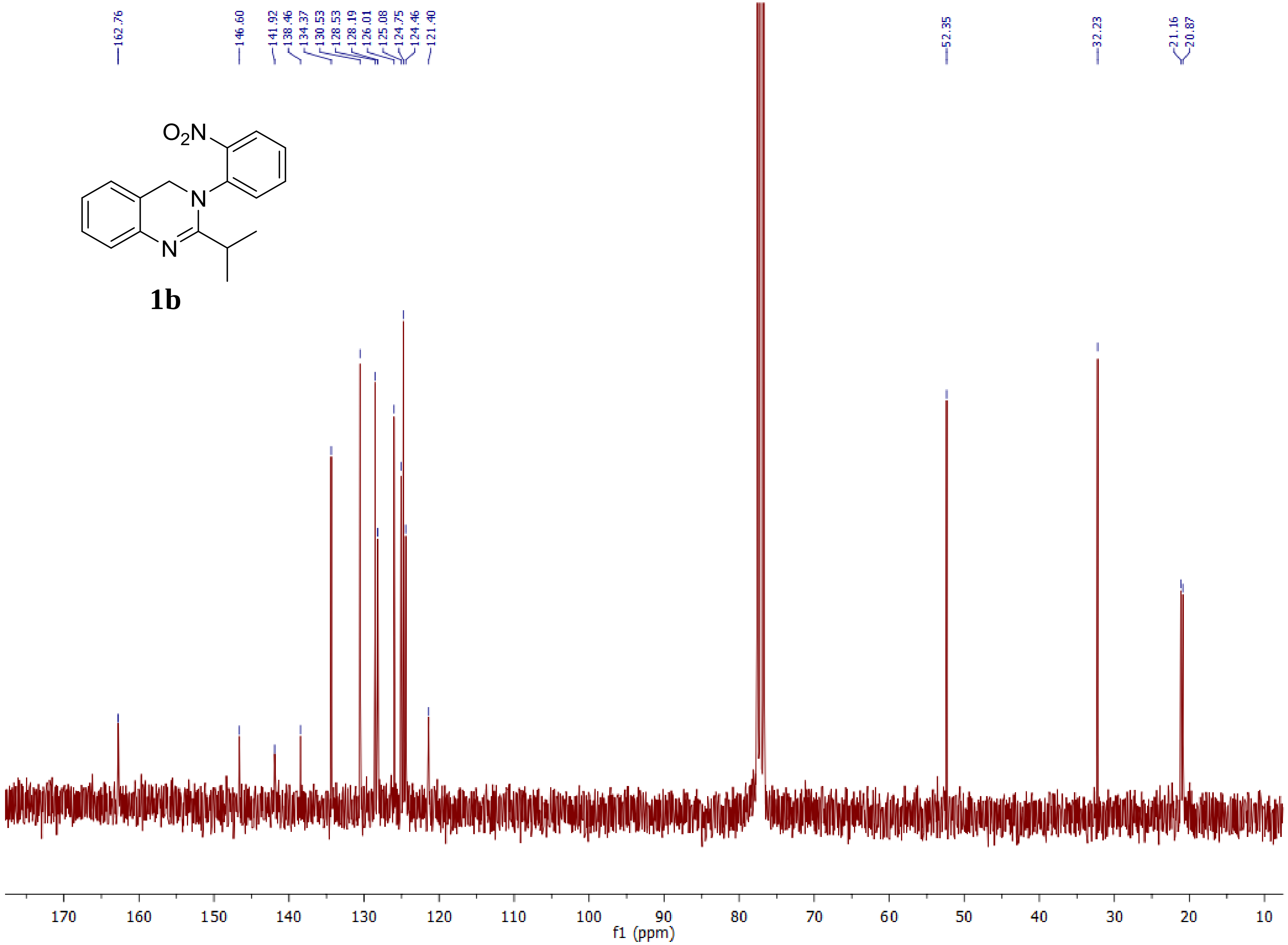

^{1}H NMR (600 MHz, $CDCl_3$) spectrum of compound **1c**

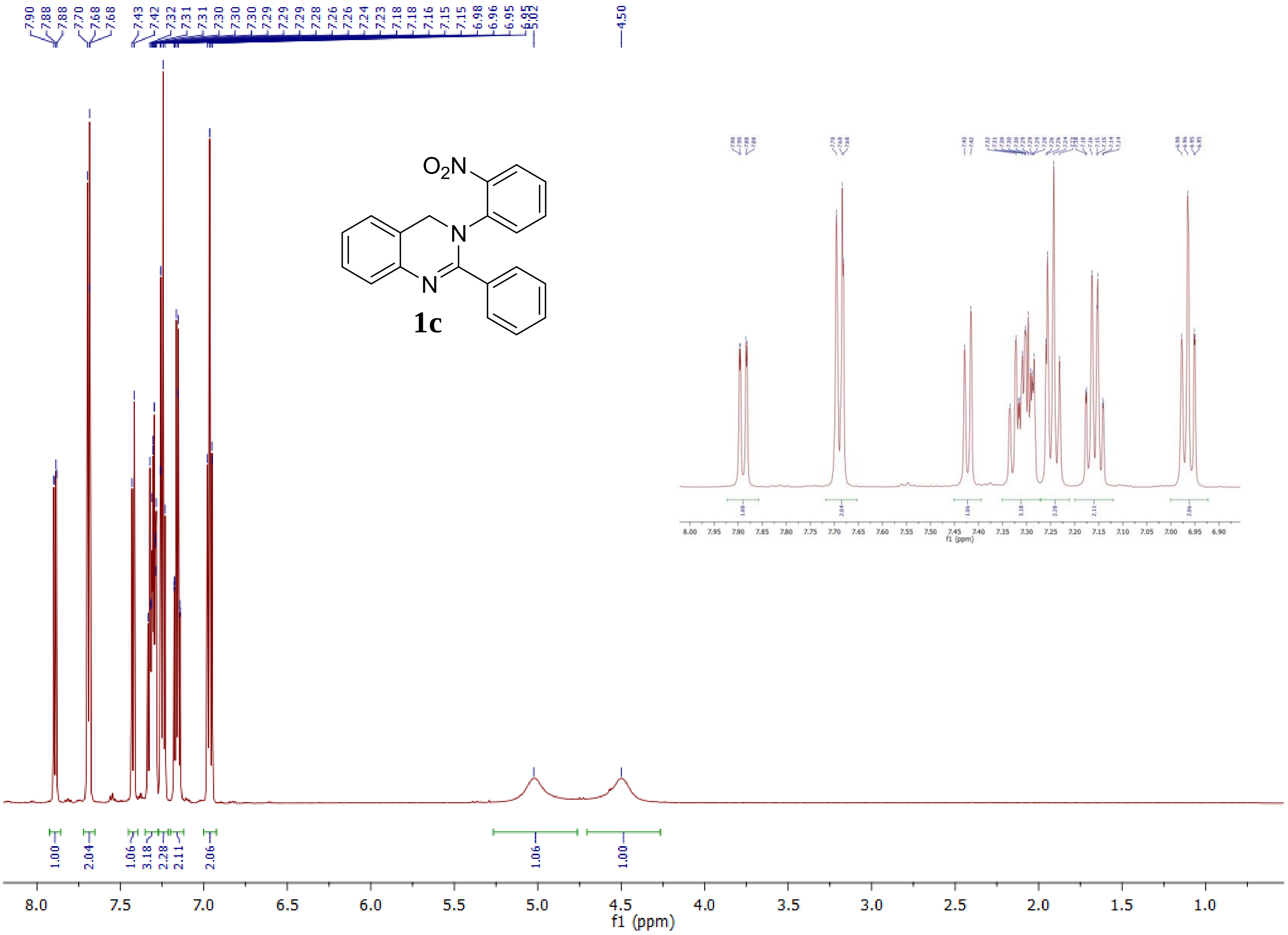

^{13}C NMR (151 MHz, $CDCl_3$) spectrum of compound **1c**

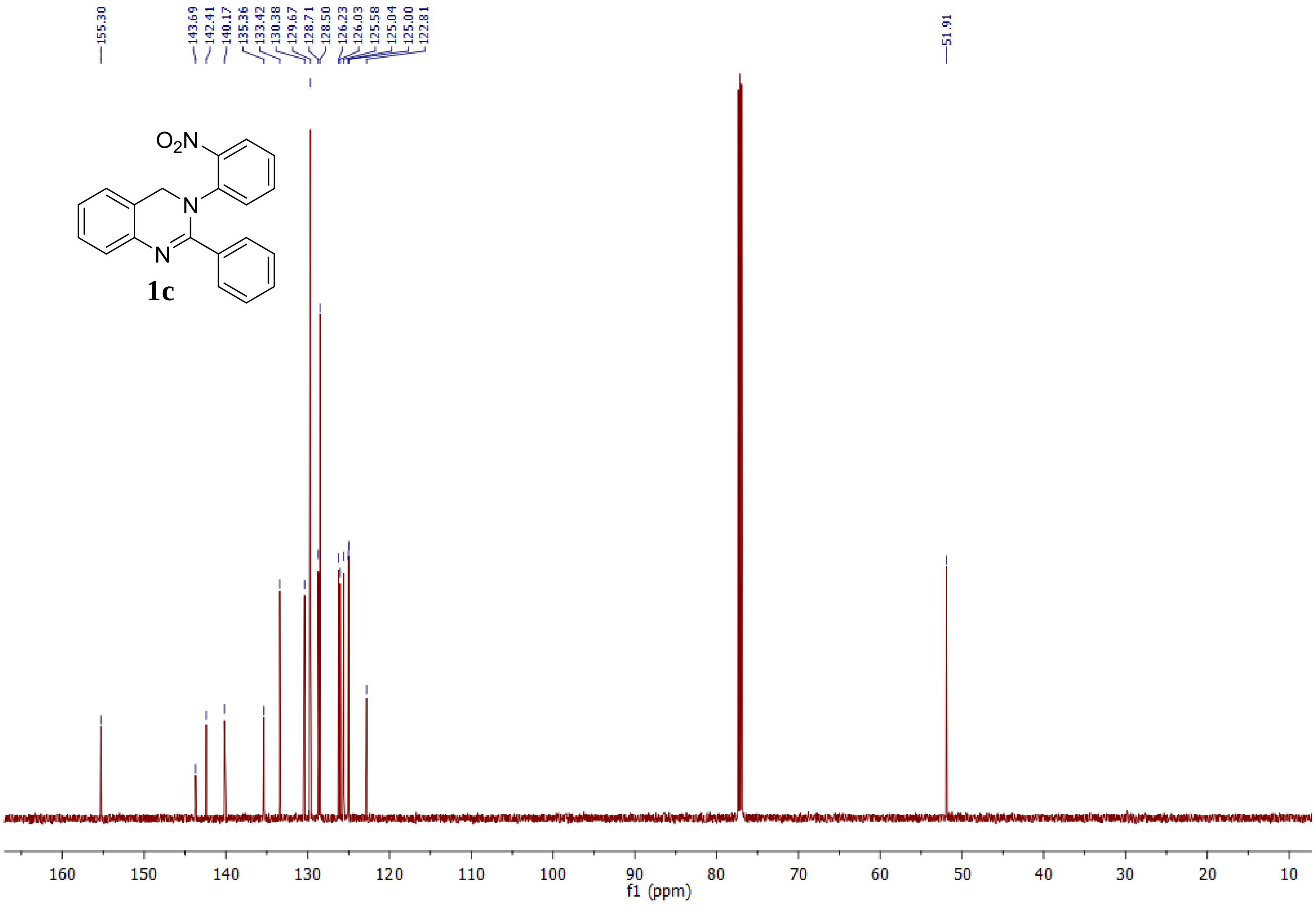

1c

^{1}H NMR (300 MHz, $CDCl_3$) spectrum of compound **1d**

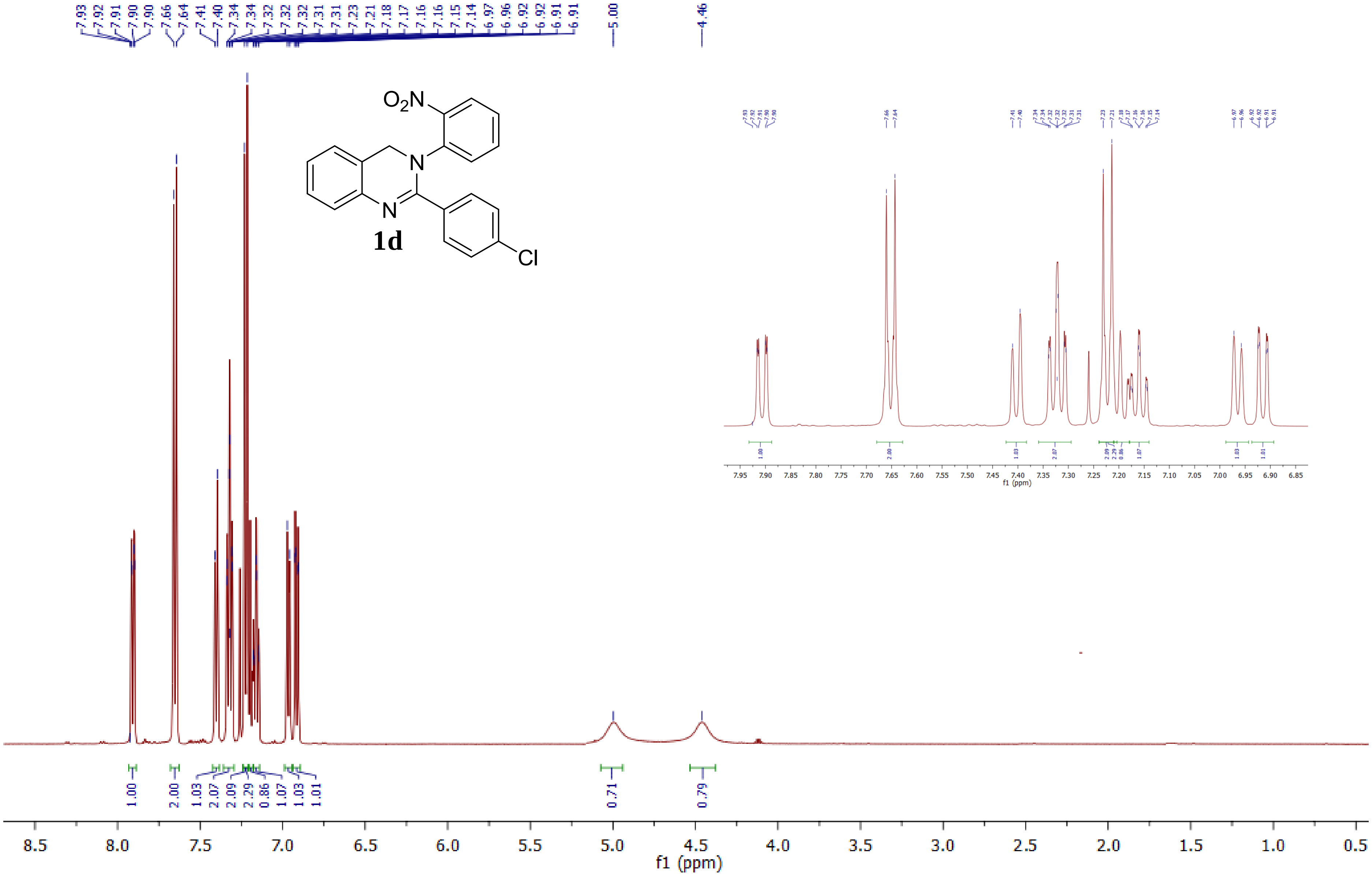

^{13}C NMR (151 MHz, $CDCl_3$) spectrum of compound **1d**

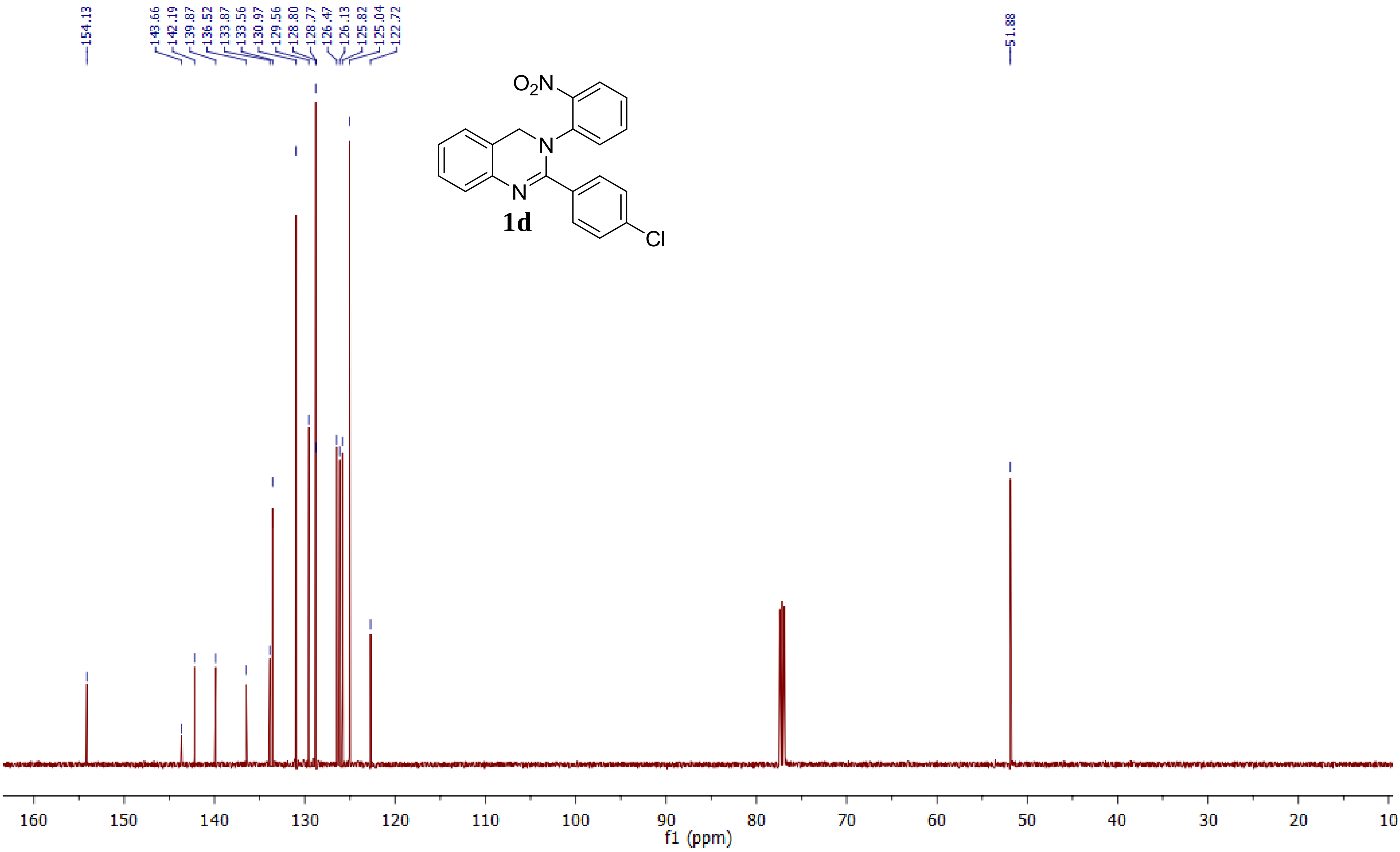

^{1}H NMR (600 MHz, $CDCl_3$) spectrum of compound **1e**

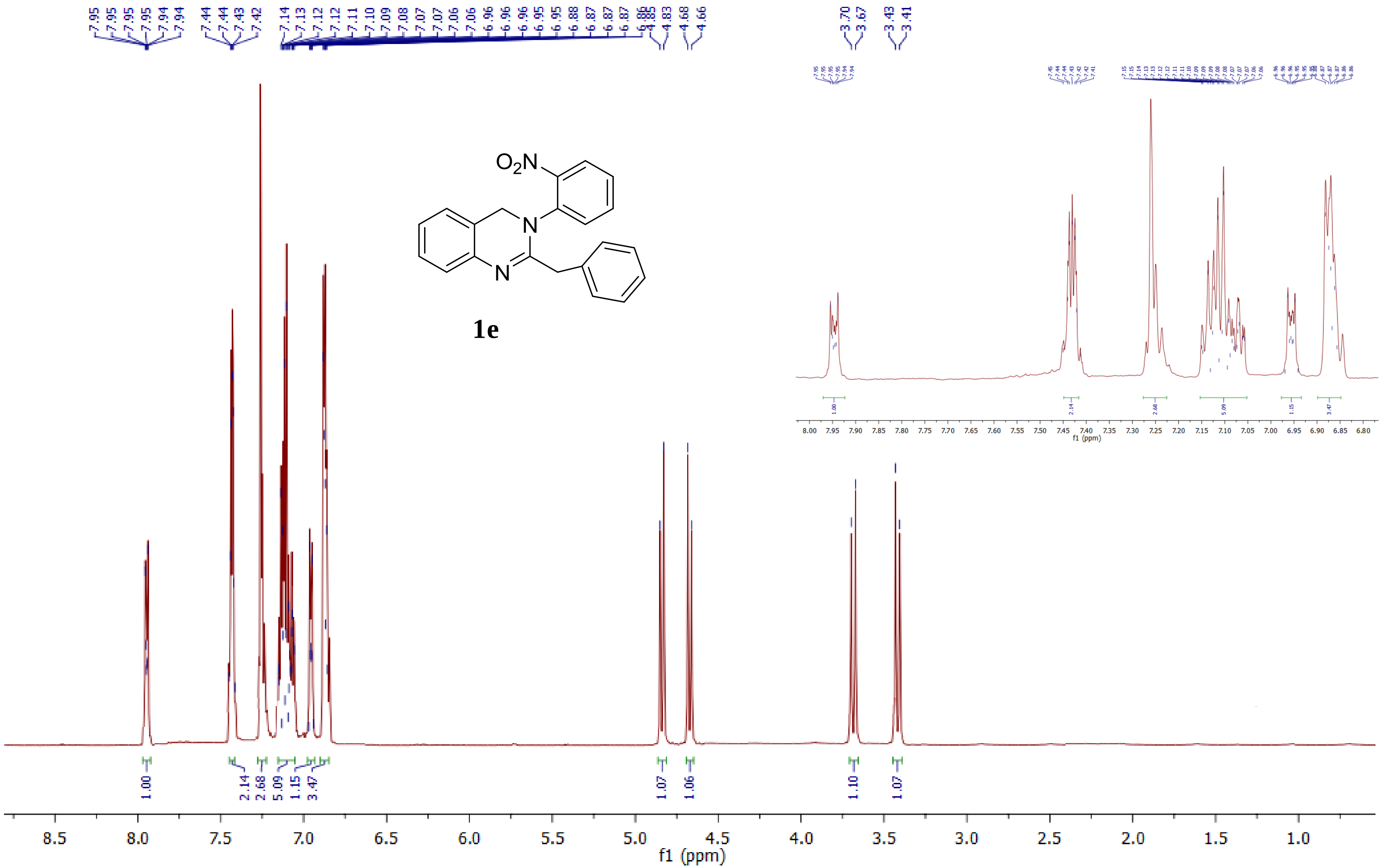

^{13}C NMR (151 MHz, $CDCl_3$) spectrum of compound **1e**

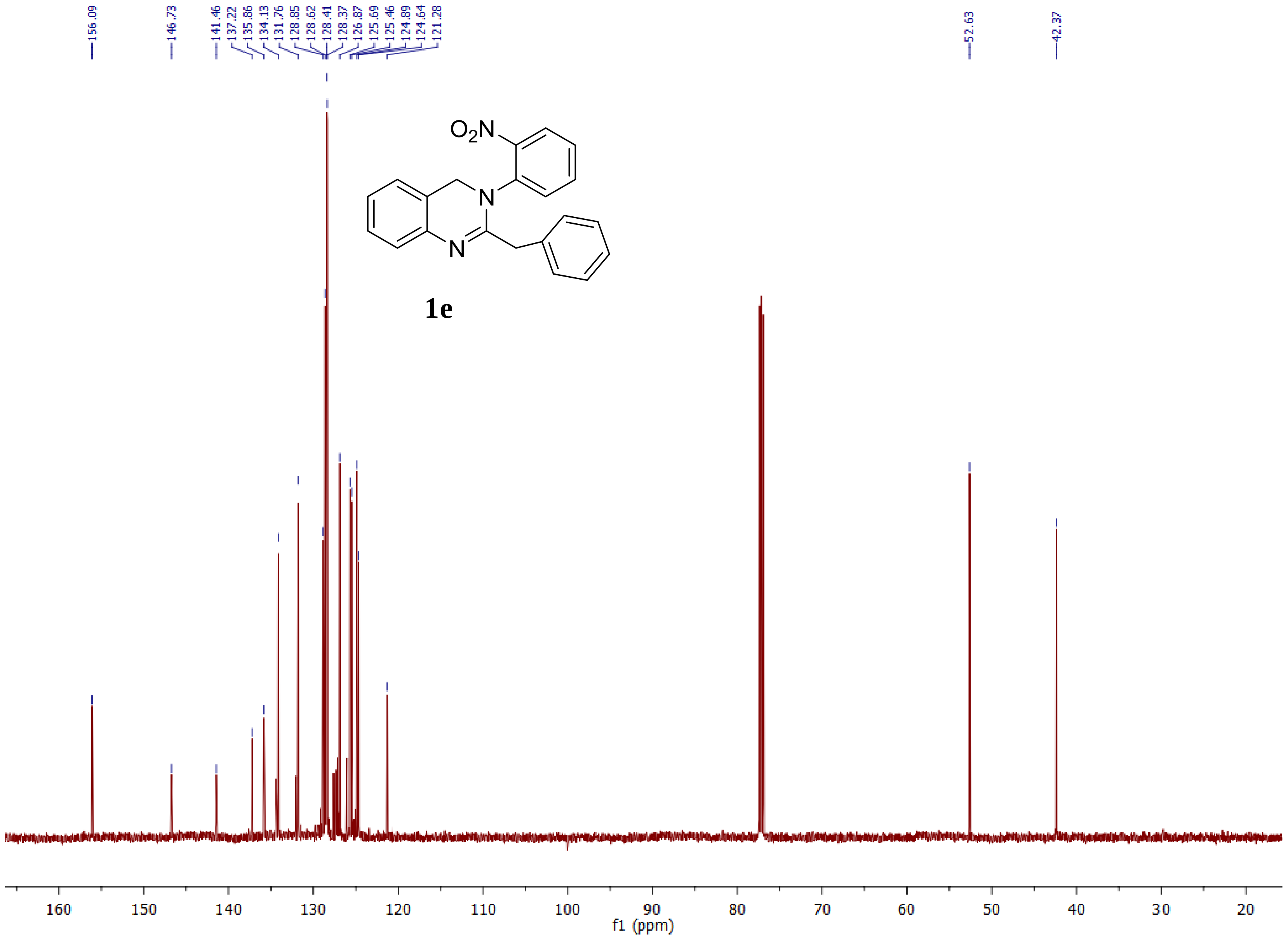

^{1}H NMR (300 MHz, $CDCl_3$) spectrum of compound **1f**

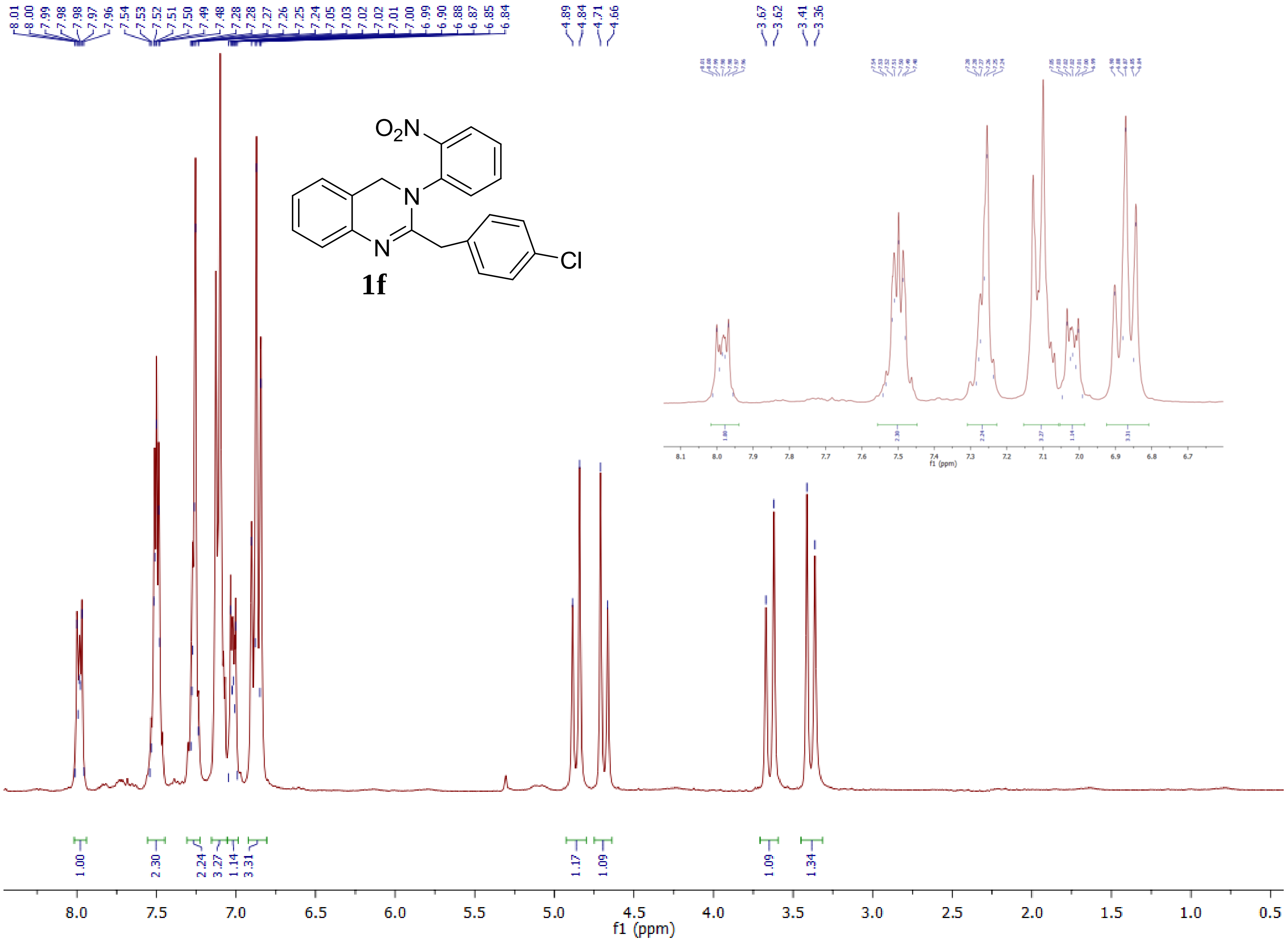

^{13}C NMR (75 MHz, $CDCl_3$) spectrum of compound **1f**

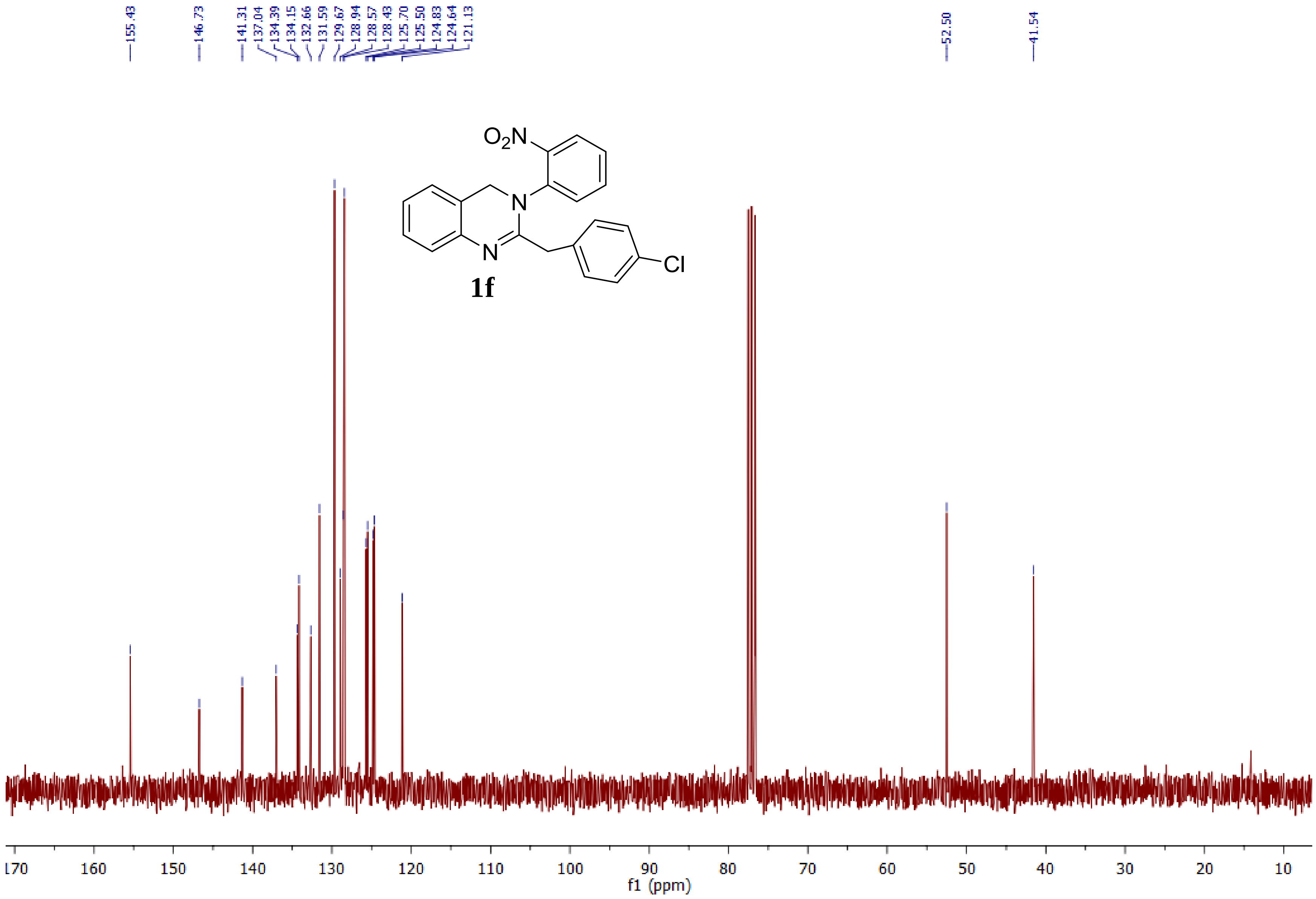

^{1}H NMR (300 MHz, $CDCl_3$) spectrum of compound **1g**

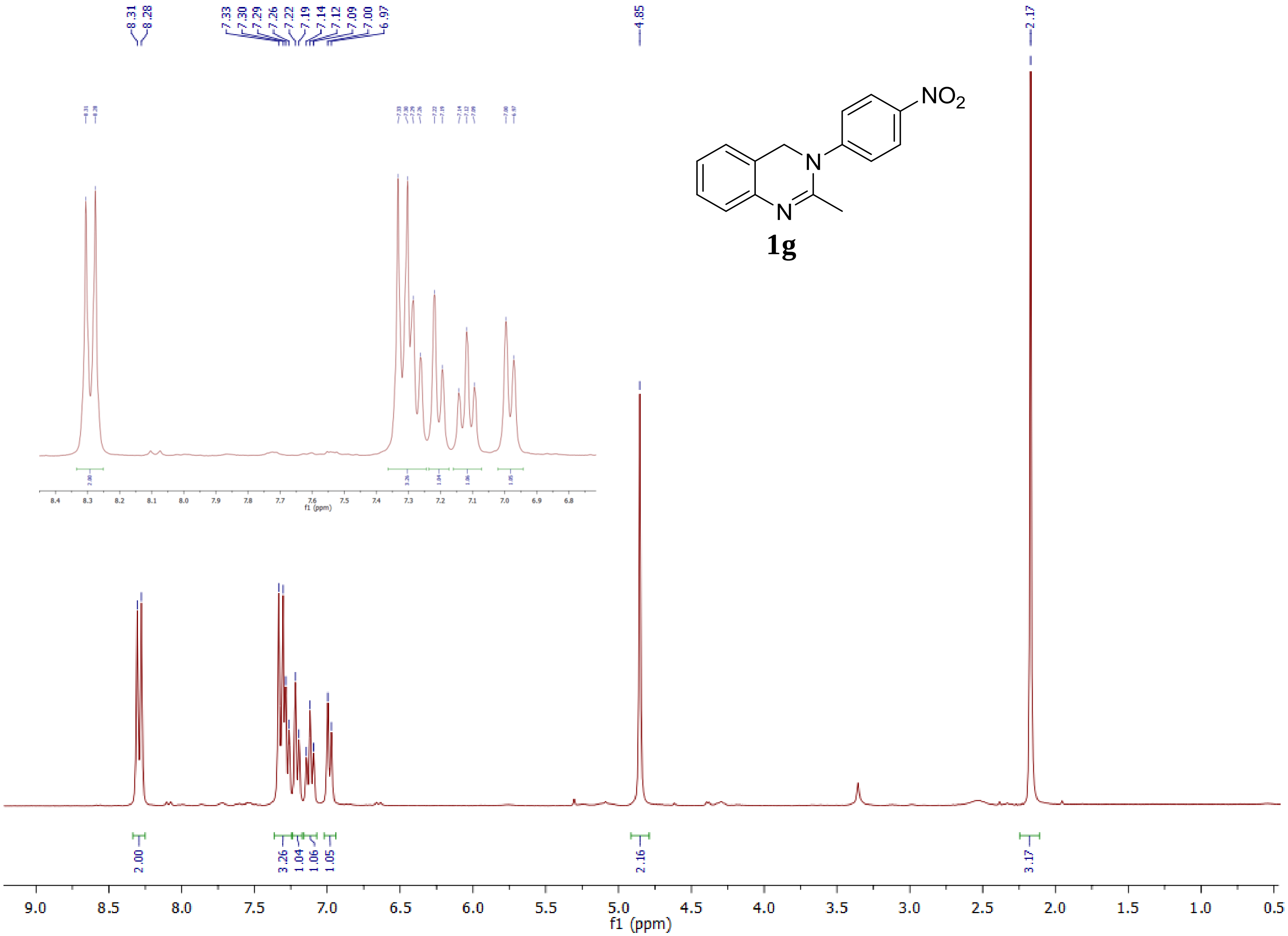

^{13}C NMR (75 MHz, $CDCl_3$) spectrum of compound **1g**

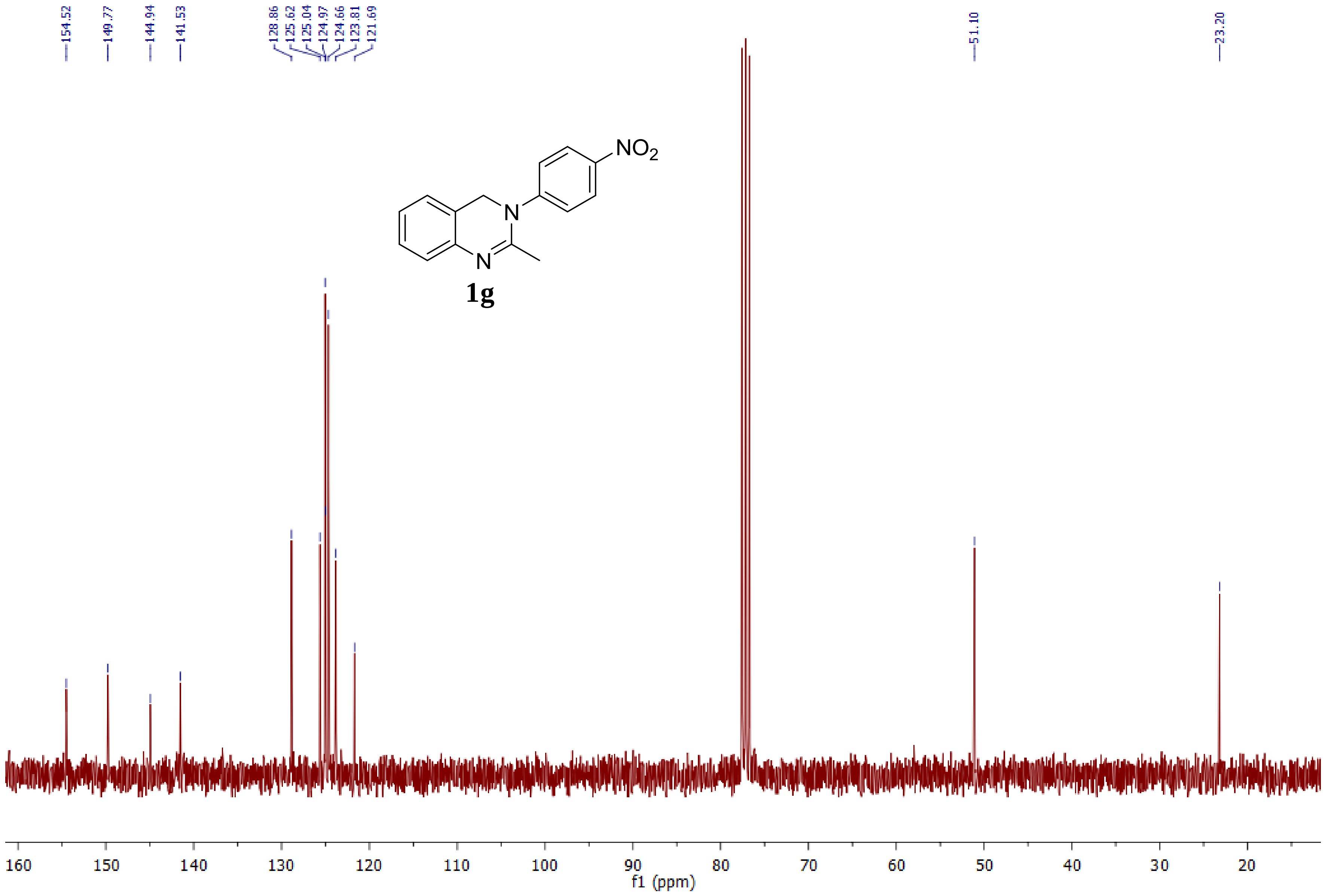

^{1}H NMR (500 MHz, $CDCl_3$) spectrum of compound **1h**

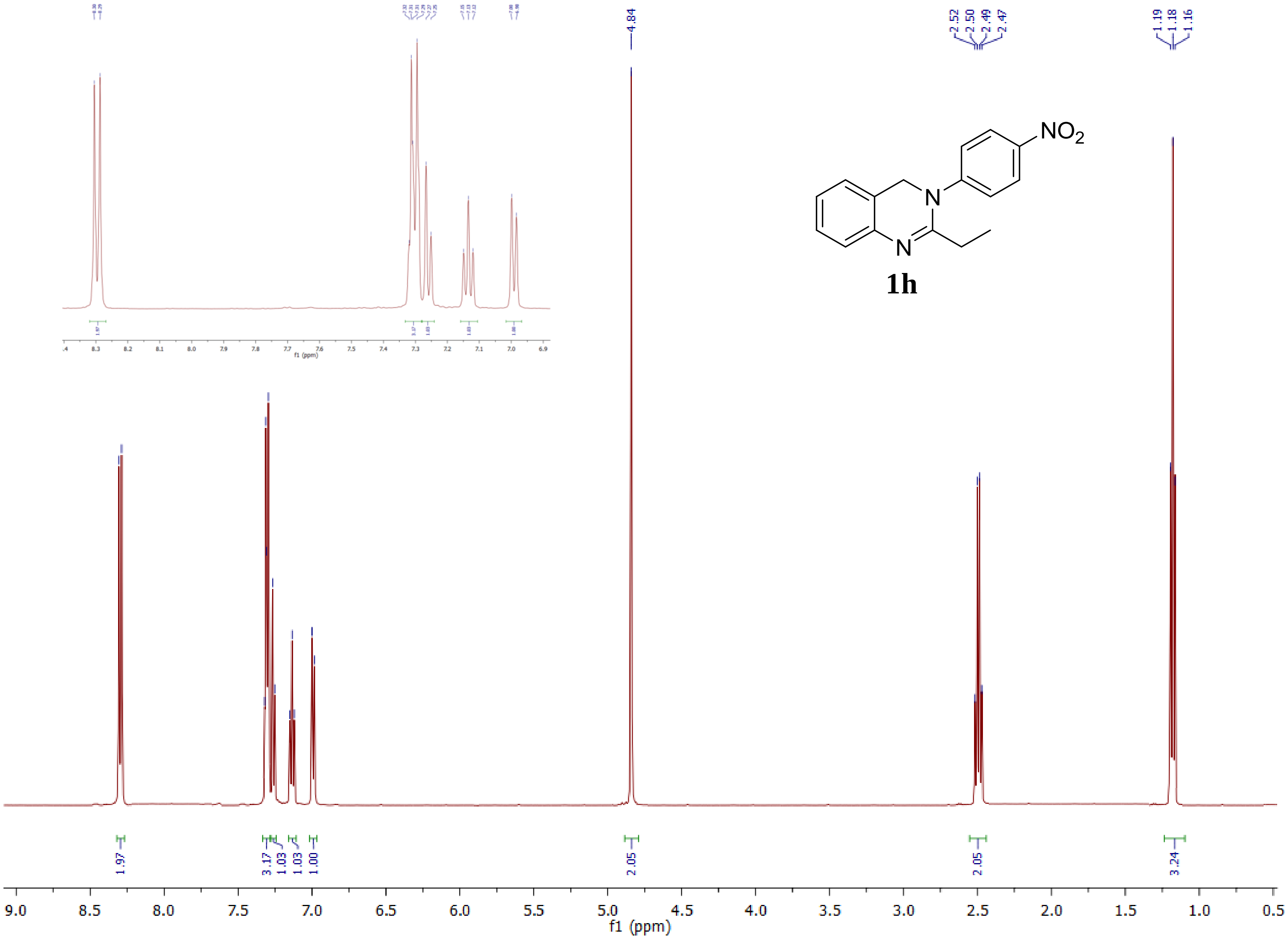

^{13}C NMR (126 MHz, $CDCl_3$) spectrum of compound **1h**

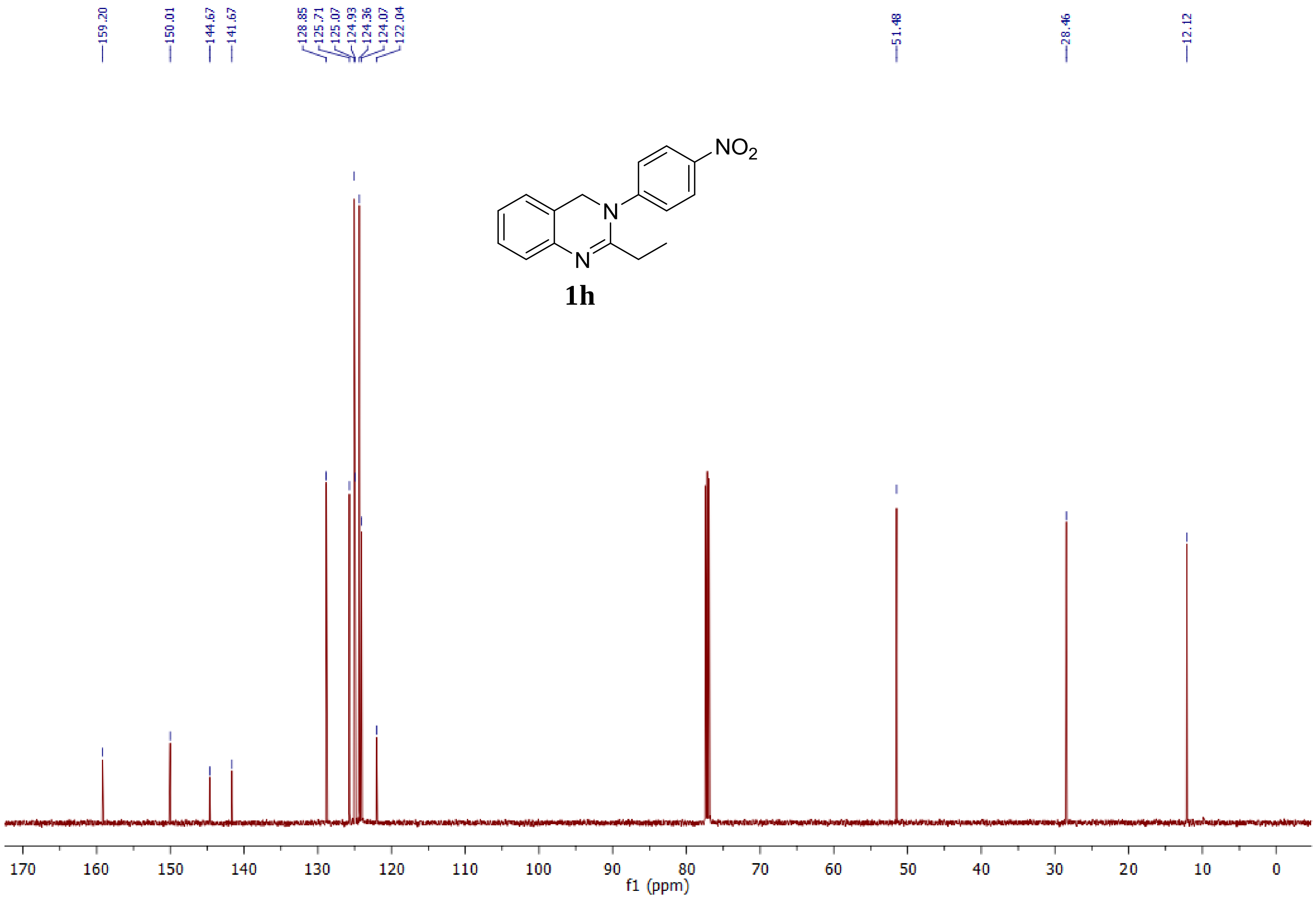

^{1}H NMR (600 MHz, $CDCl_3$) spectrum of compound **1i**

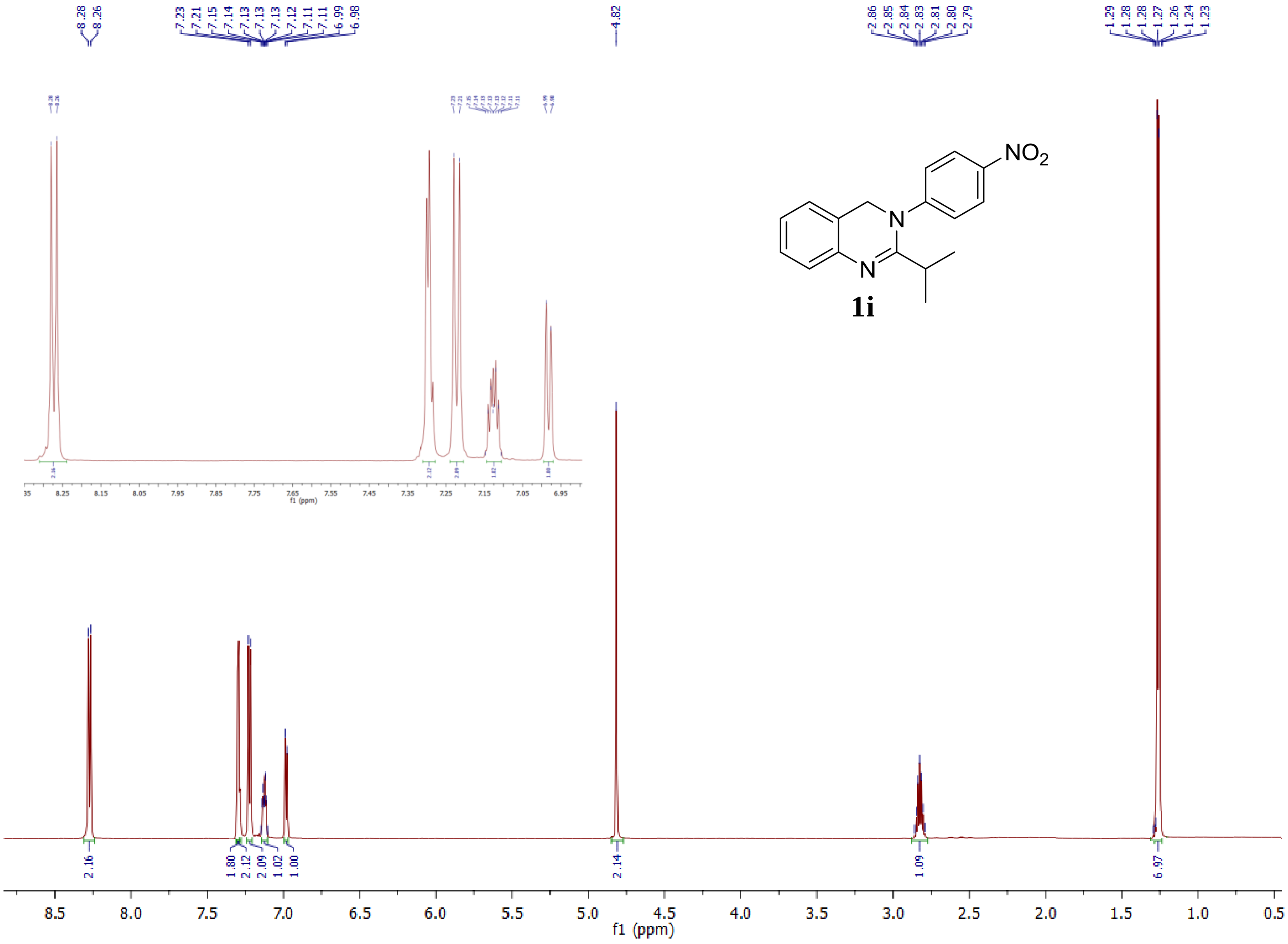

^{13}C NMR (126 MHz, $CDCl_3$) spectrum of compound **1i**

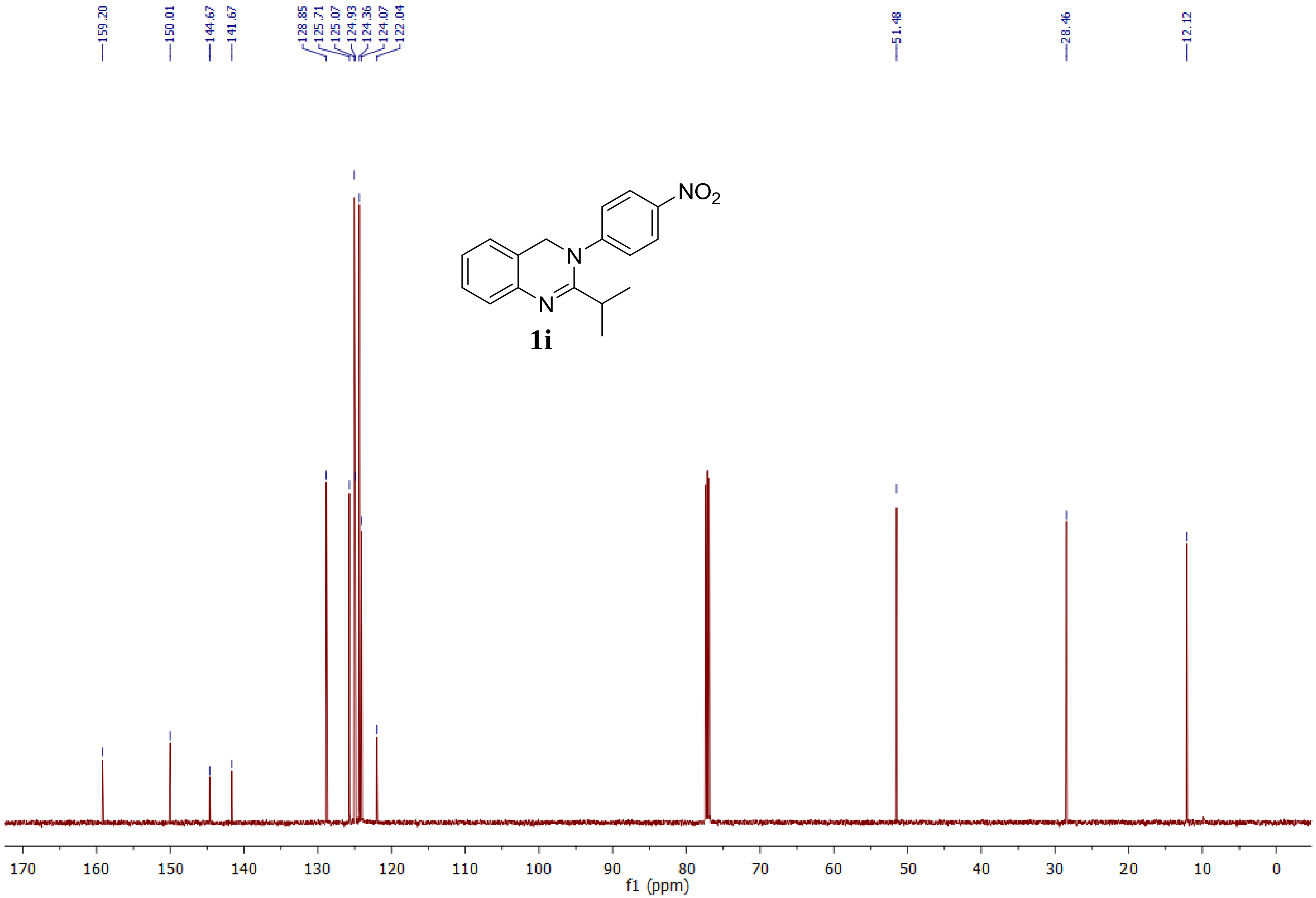

^{1}H NMR (500 MHz, $CDCl_3$) spectrum of compound **1j**

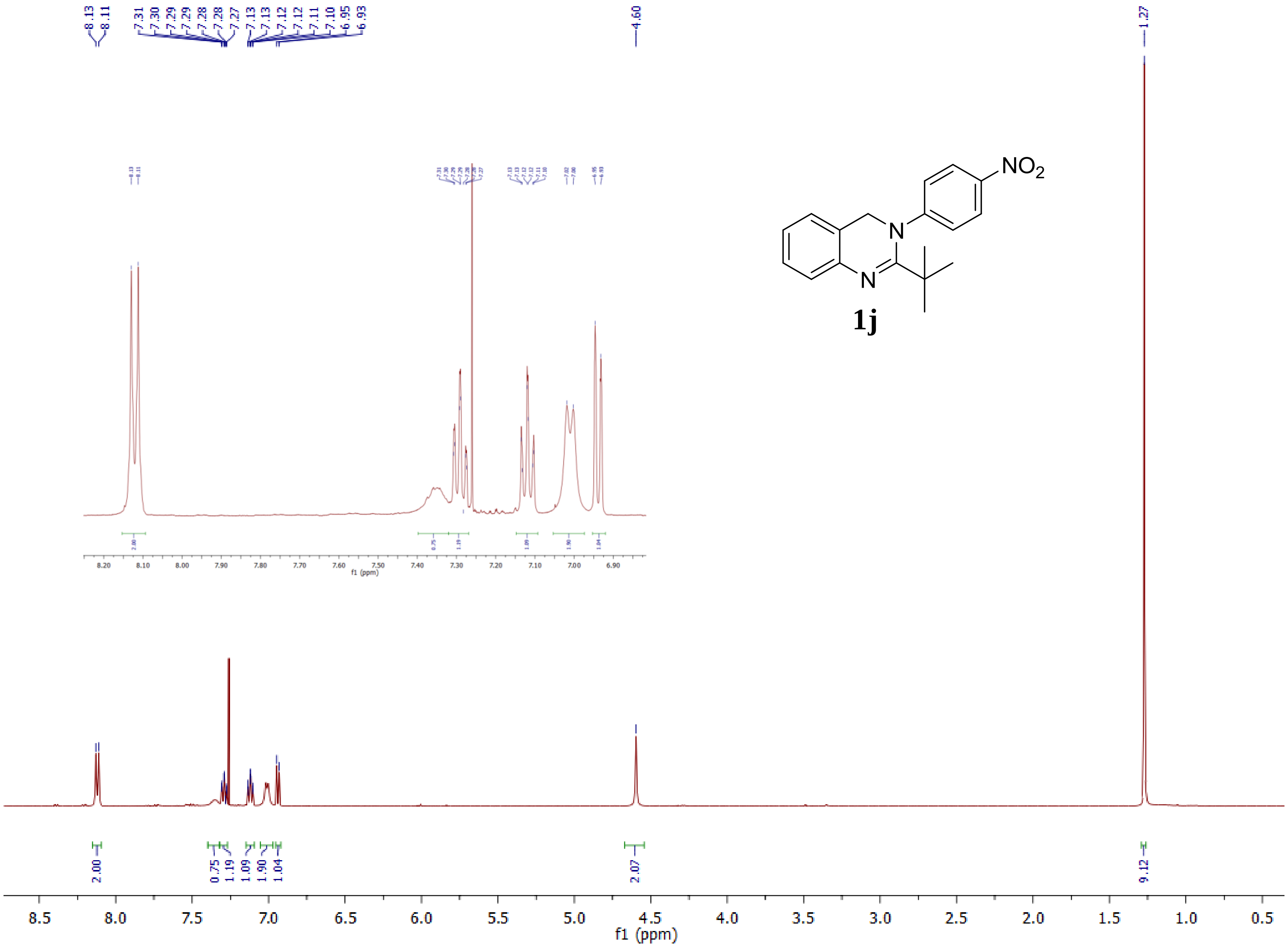

^{13}C NMR (126 MHz, $CDCl_3$) spectrum of compound **1j**

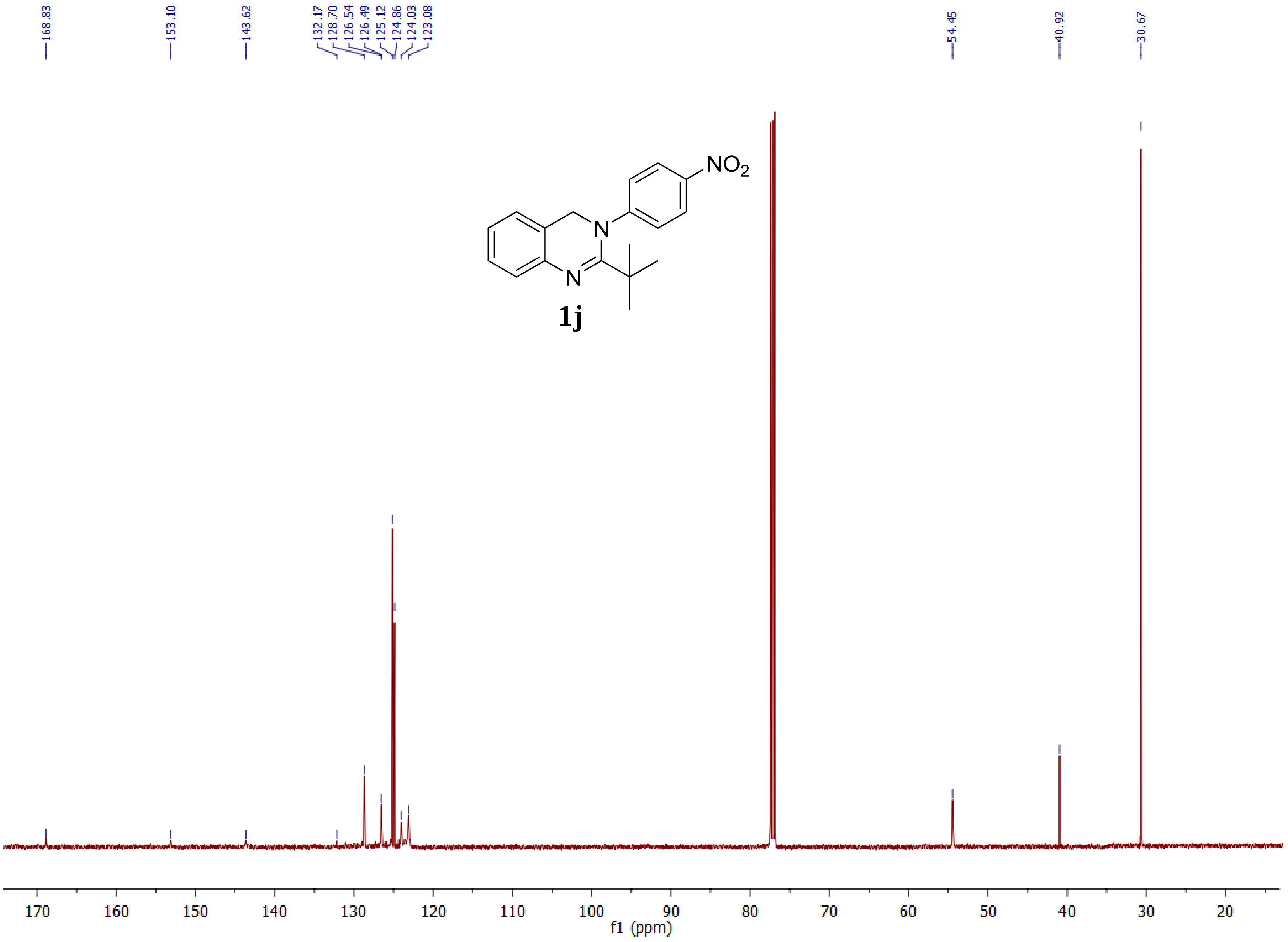

^{1}H NMR (600 MHz, $CDCl_3$) spectrum of compound **1k**

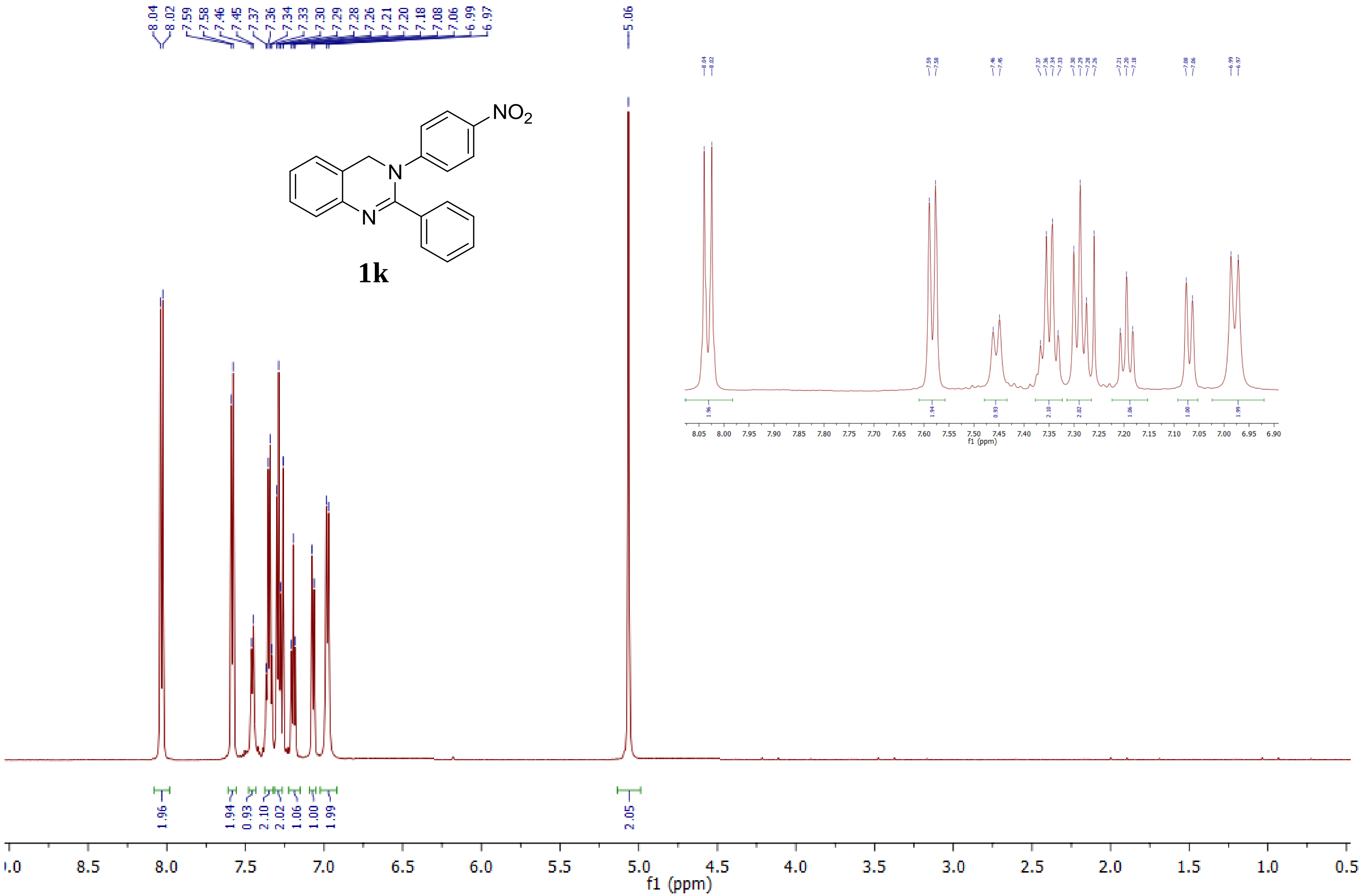

^{13}C NMR (151 MHz, $CDCl_3$) spectrum of compound **1k**

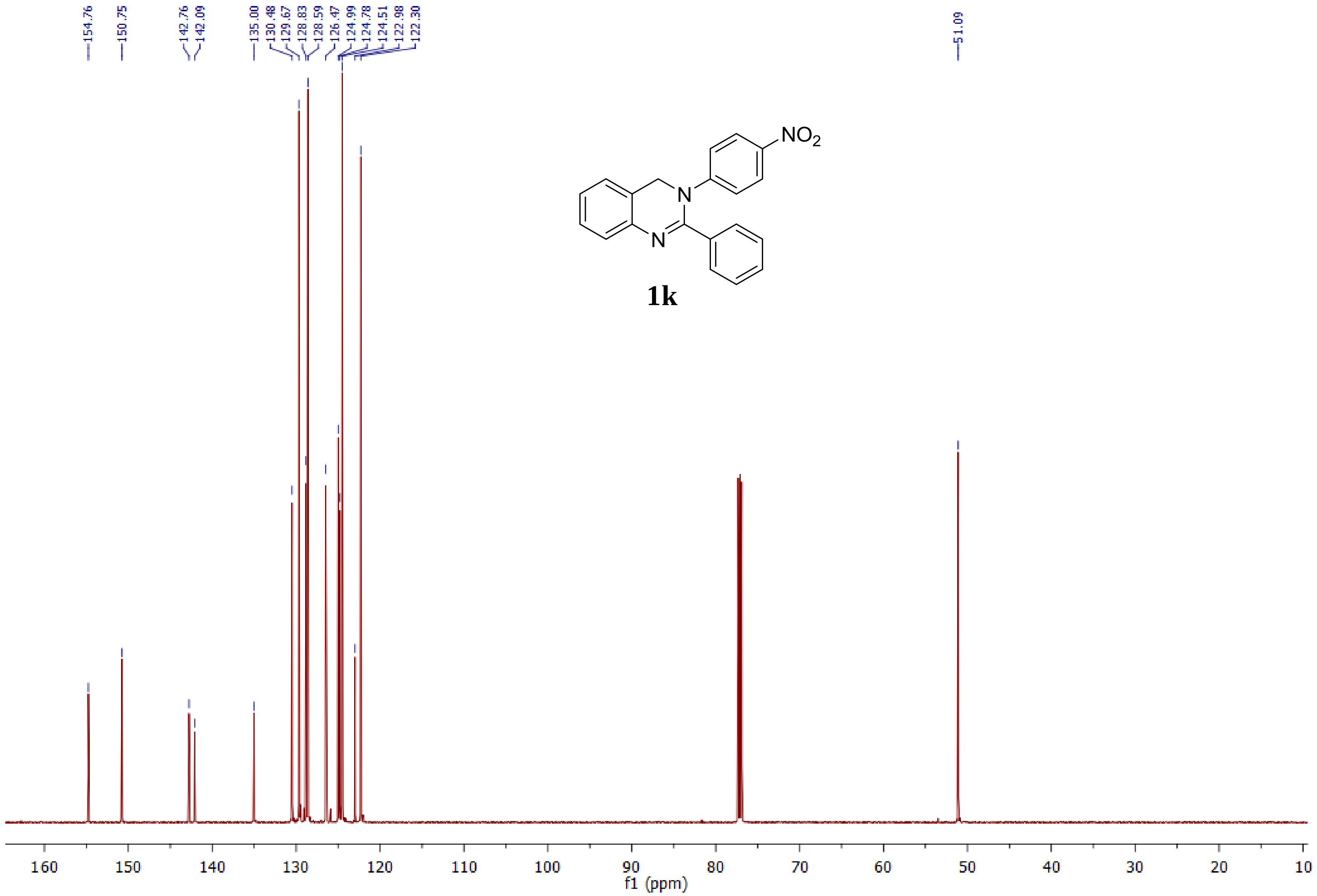

^{1}H NMR (300 MHz, $CDCl_3$) spectrum of compound **1l**

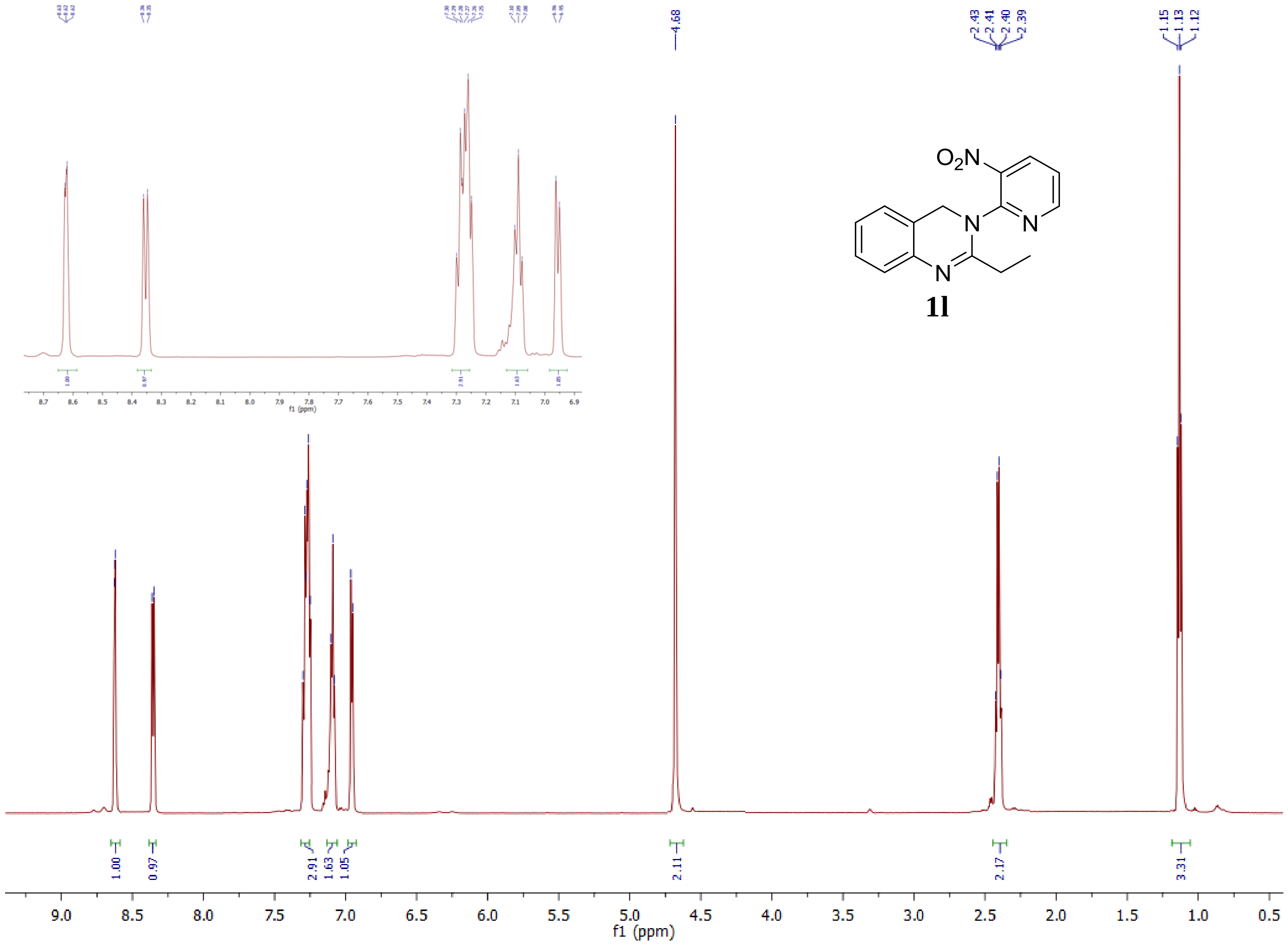

^{13}C NMR (75 MHz, $CDCl_3$) spectrum of compound **1l**

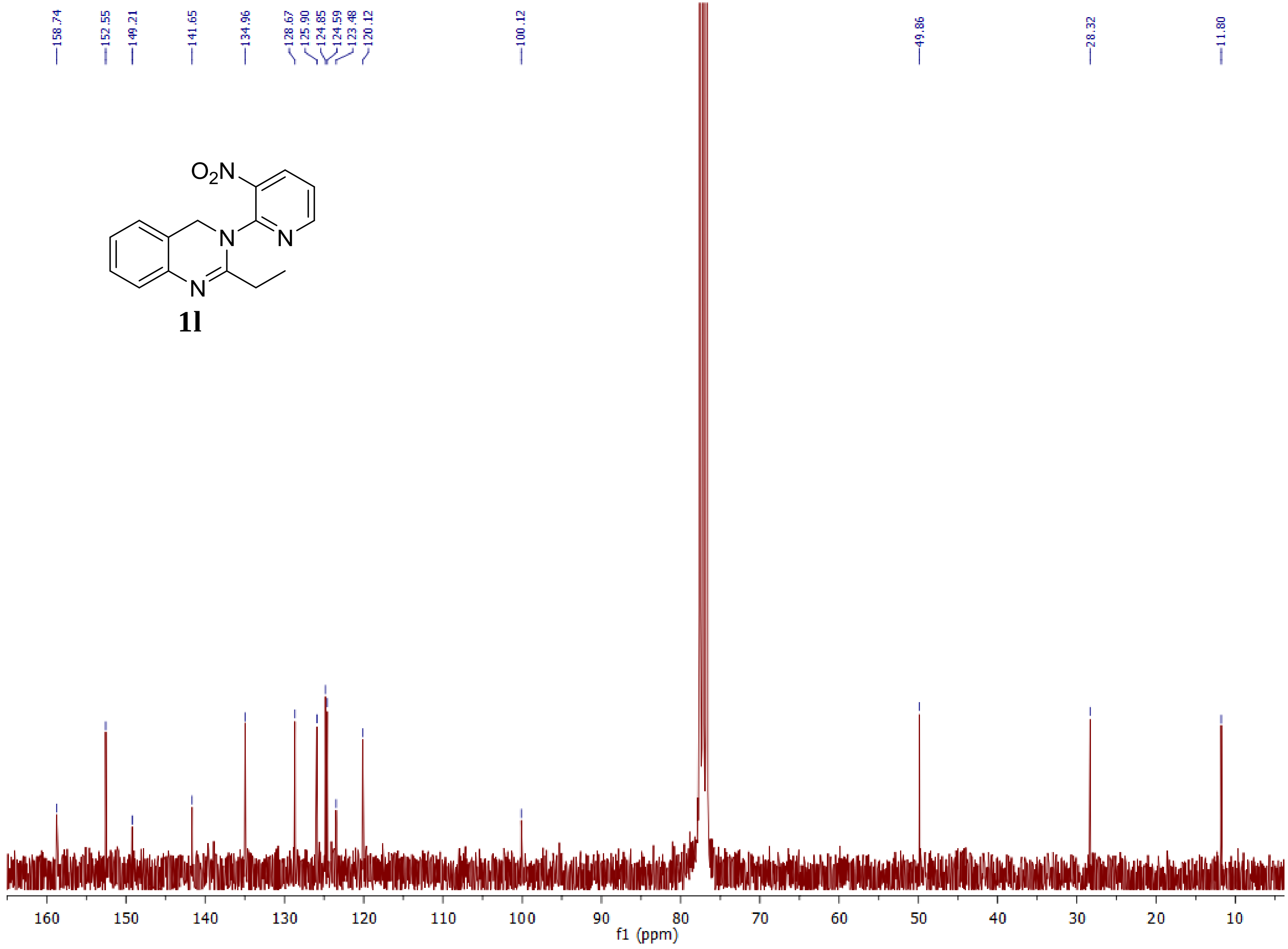

^{1}H NMR (600 MHz, $CDCl_3$) spectrum of compound **1m**

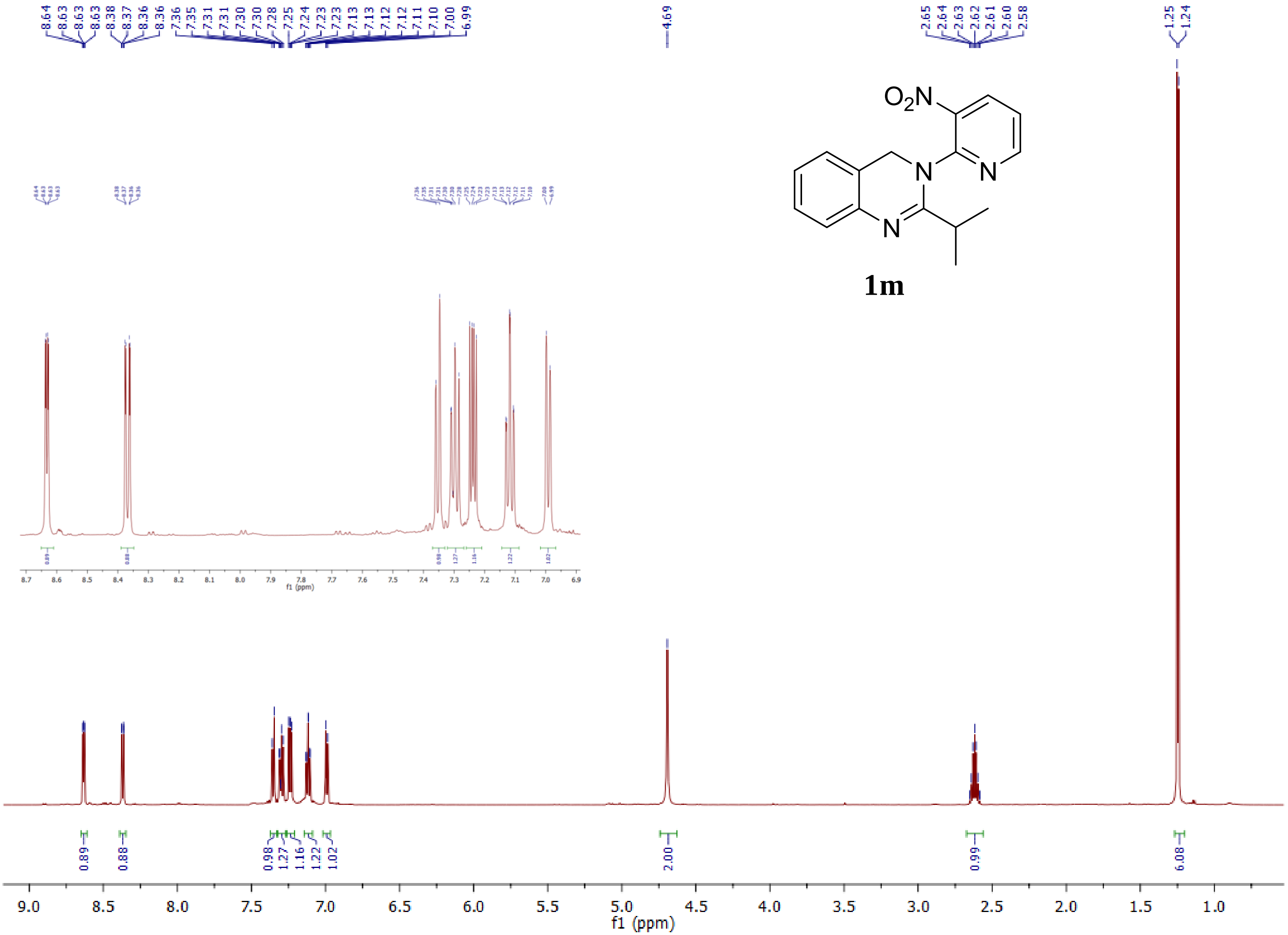

^{13}C NMR (151 MHz, $CDCl_3$) spectrum of compound **1m**

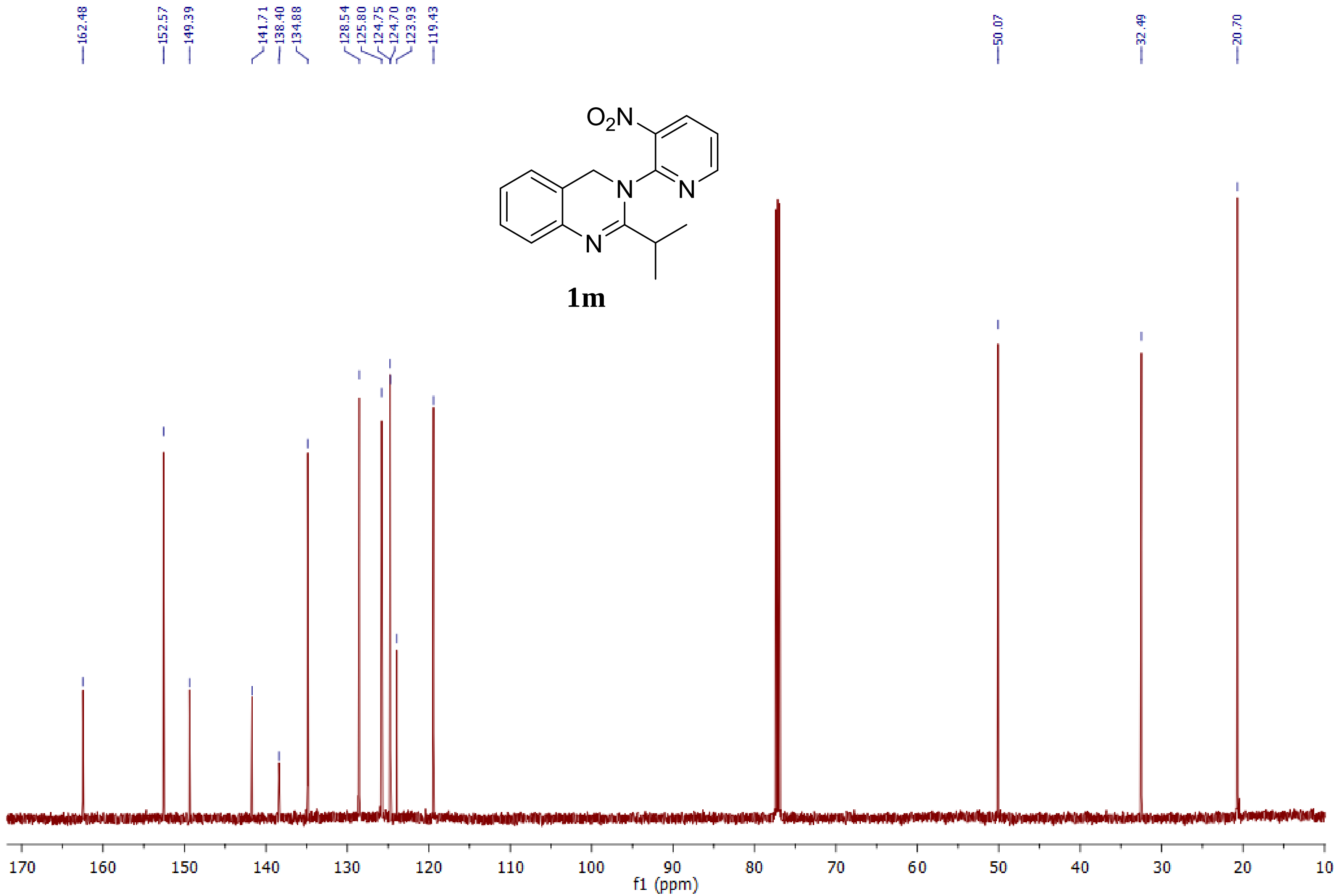

^{1}H NMR (600 MHz, $CDCl_3$) spectrum of compound **1n**

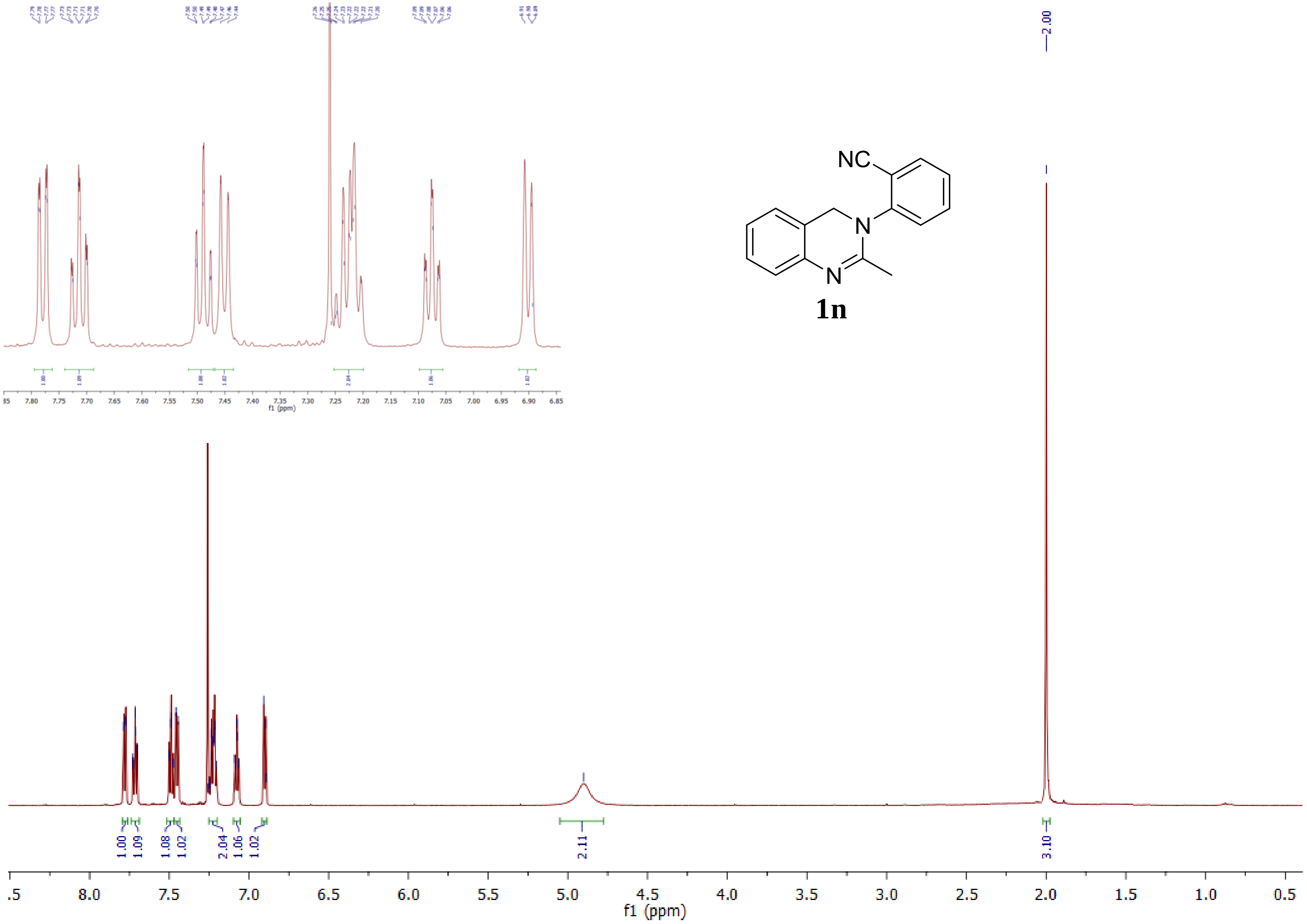

^{13}C NMR (151 MHz, $CDCl_3$) spectrum of compound **1n**

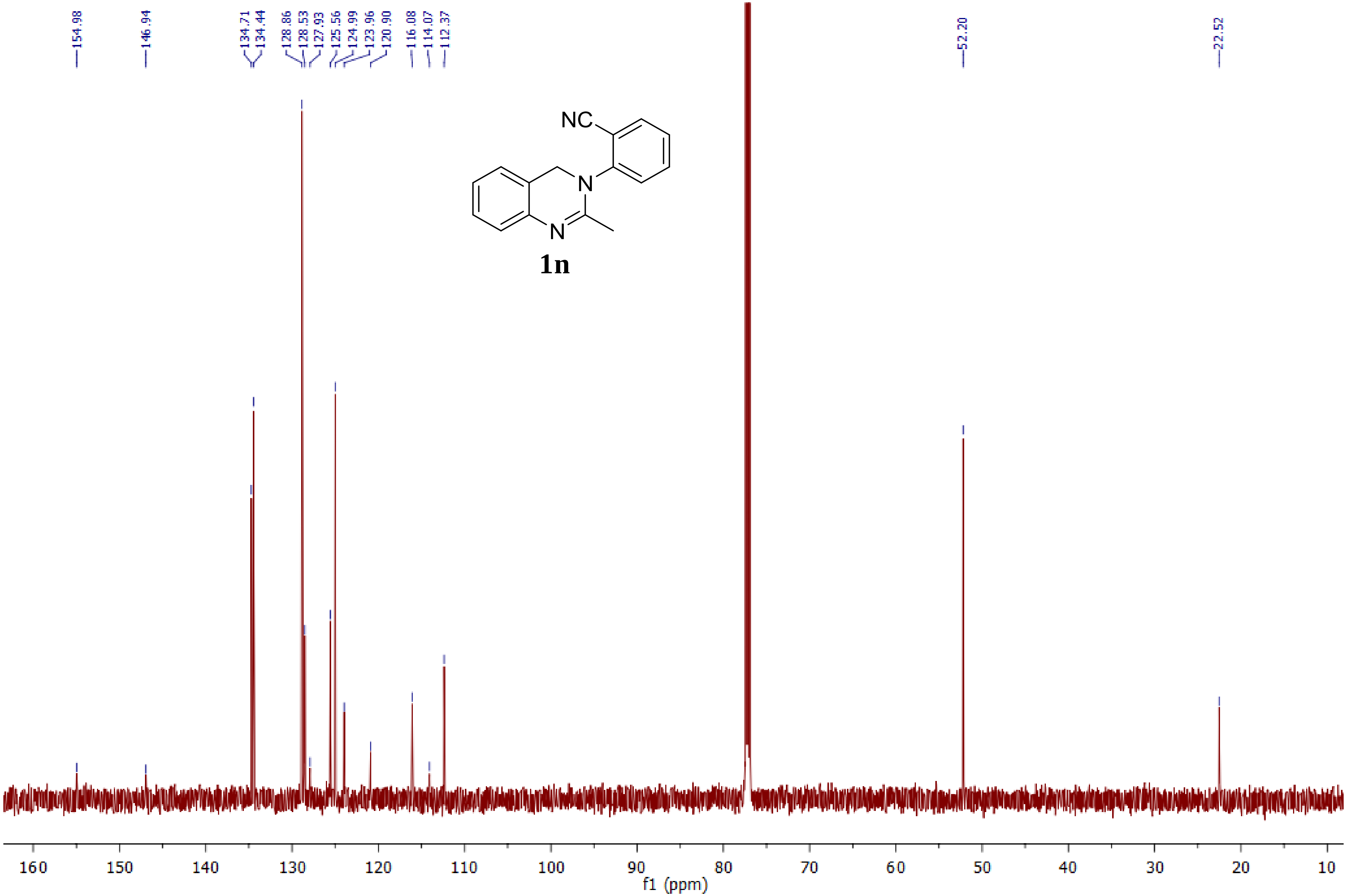

^{1}H NMR (600 MHz, $CDCl_3$) spectrum of compound **1o**

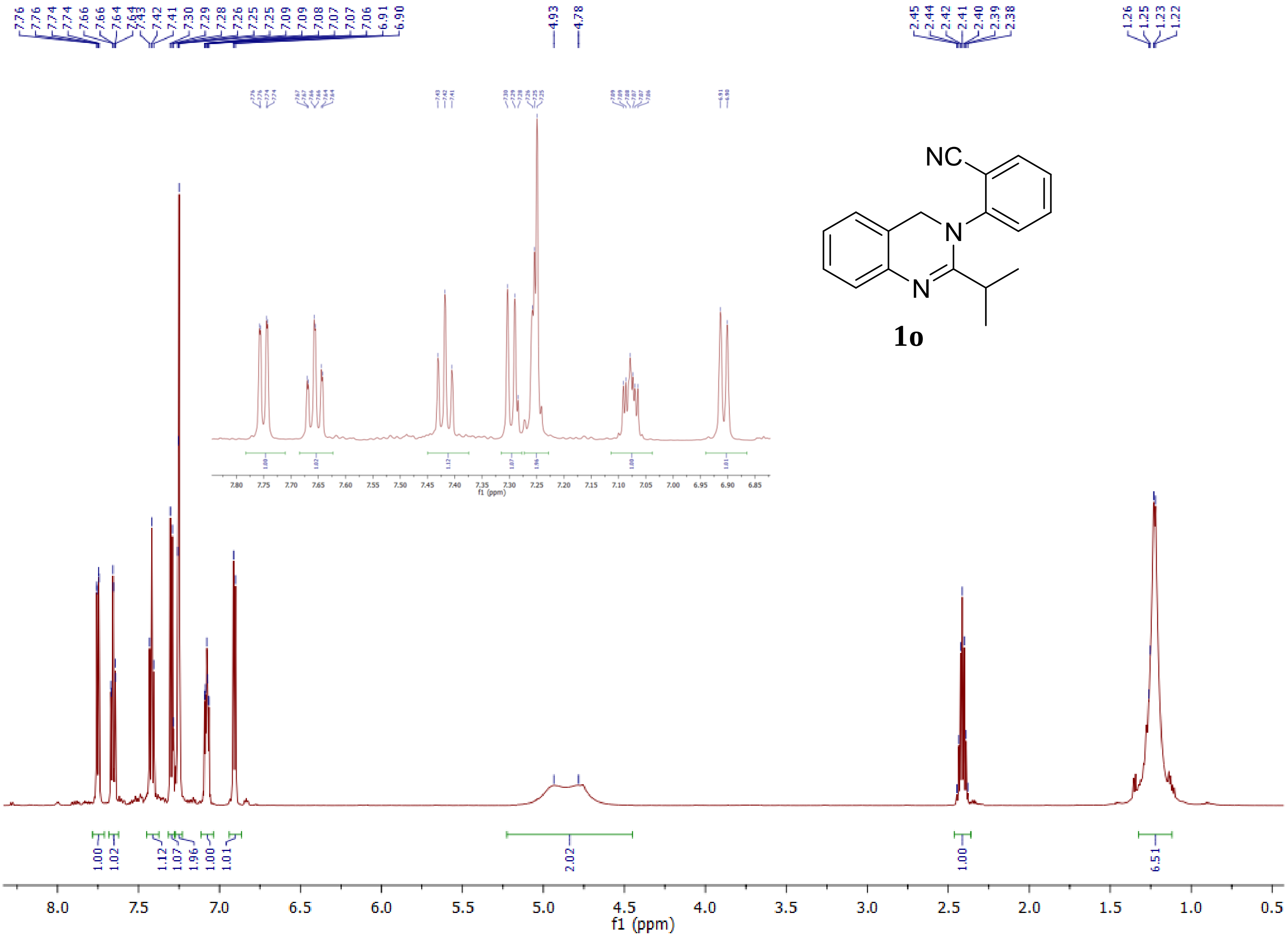

1o

^{13}C NMR (151 MHz, $CDCl_3$) spectrum of compound **1o**

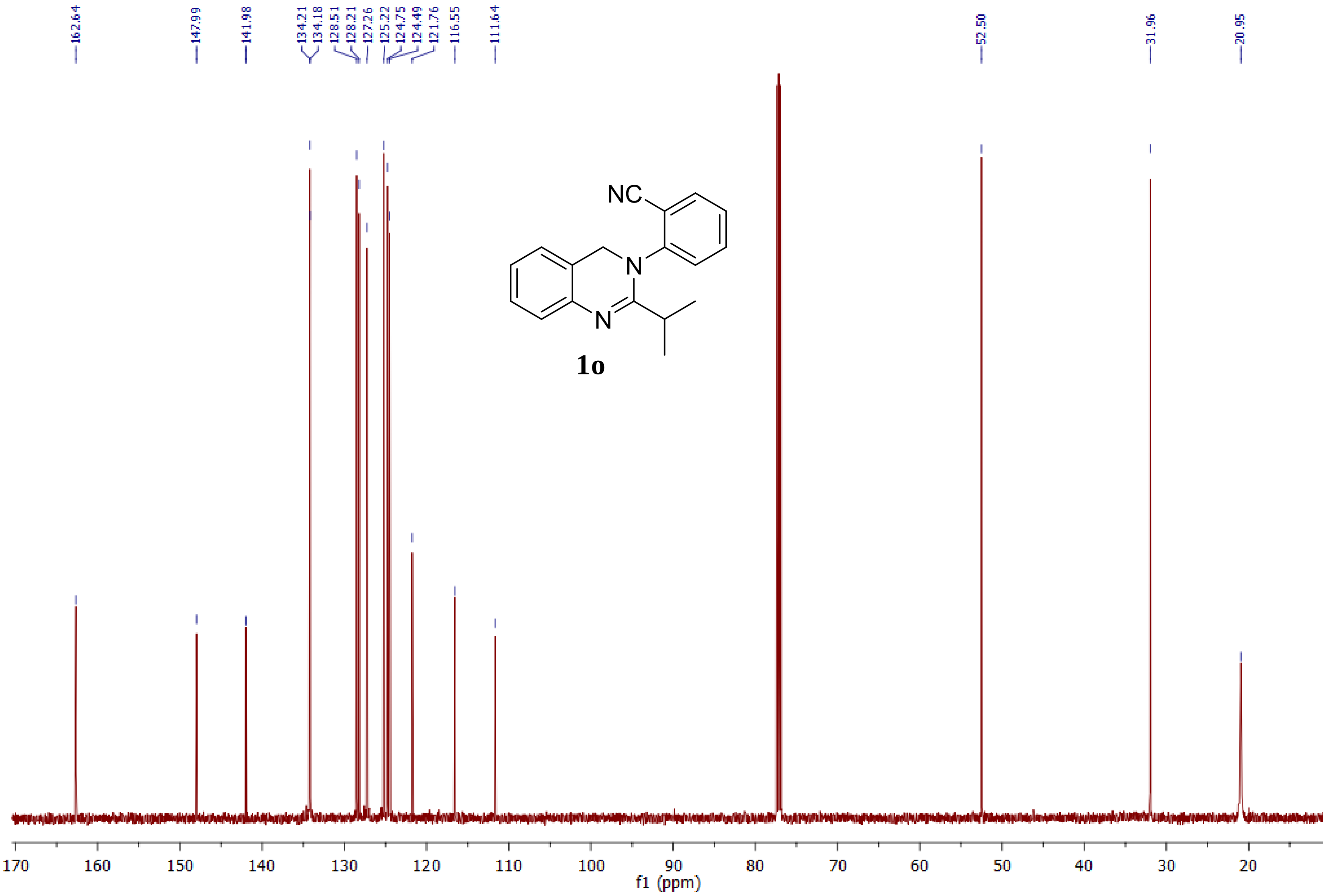

^{1}H NMR (500 MHz, $CDCl_3$) spectrum of compound **2a**

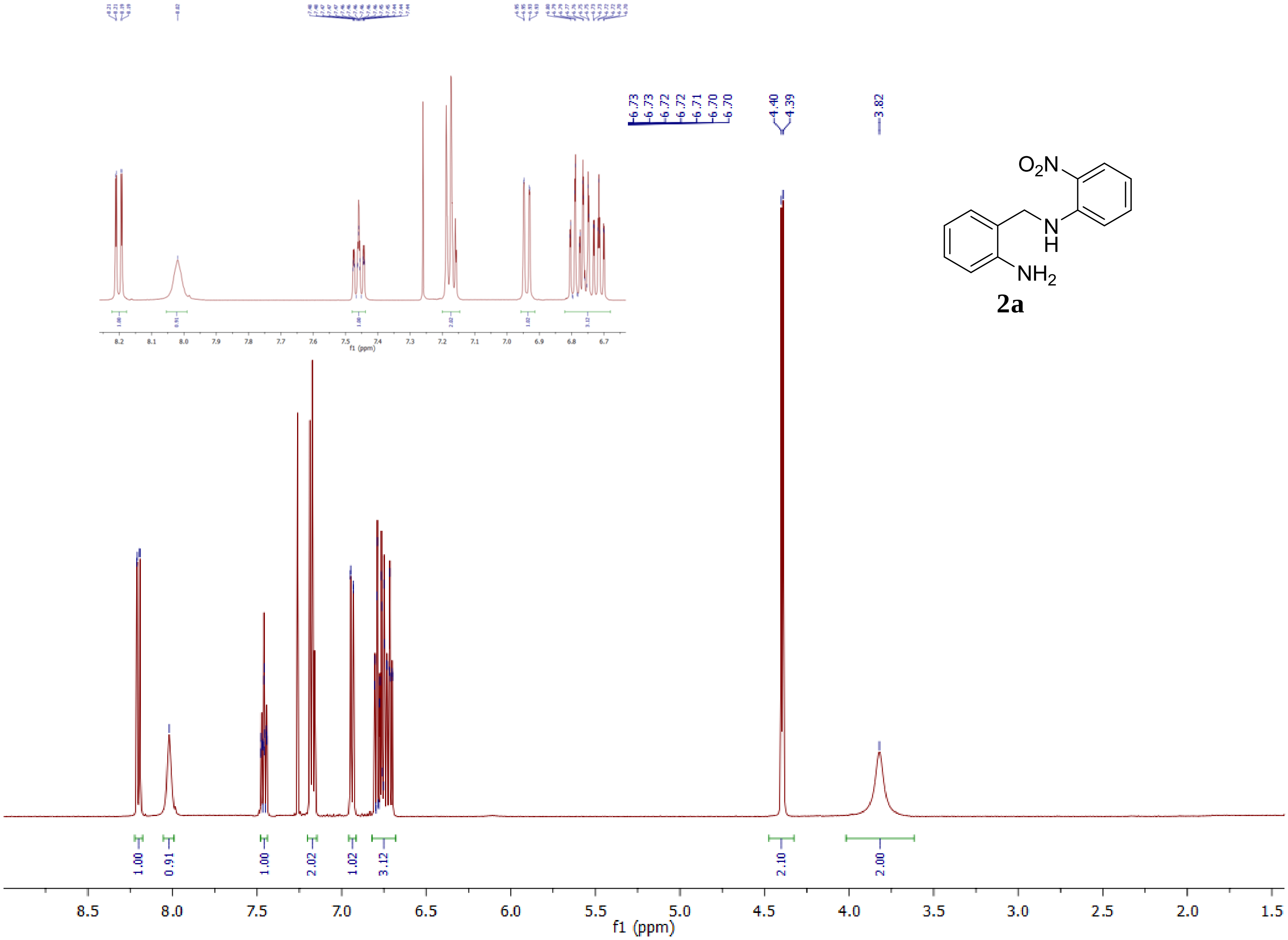

2a

^{13}C NMR (126 MHz, $CDCl_3$) spectrum of compound **2a**

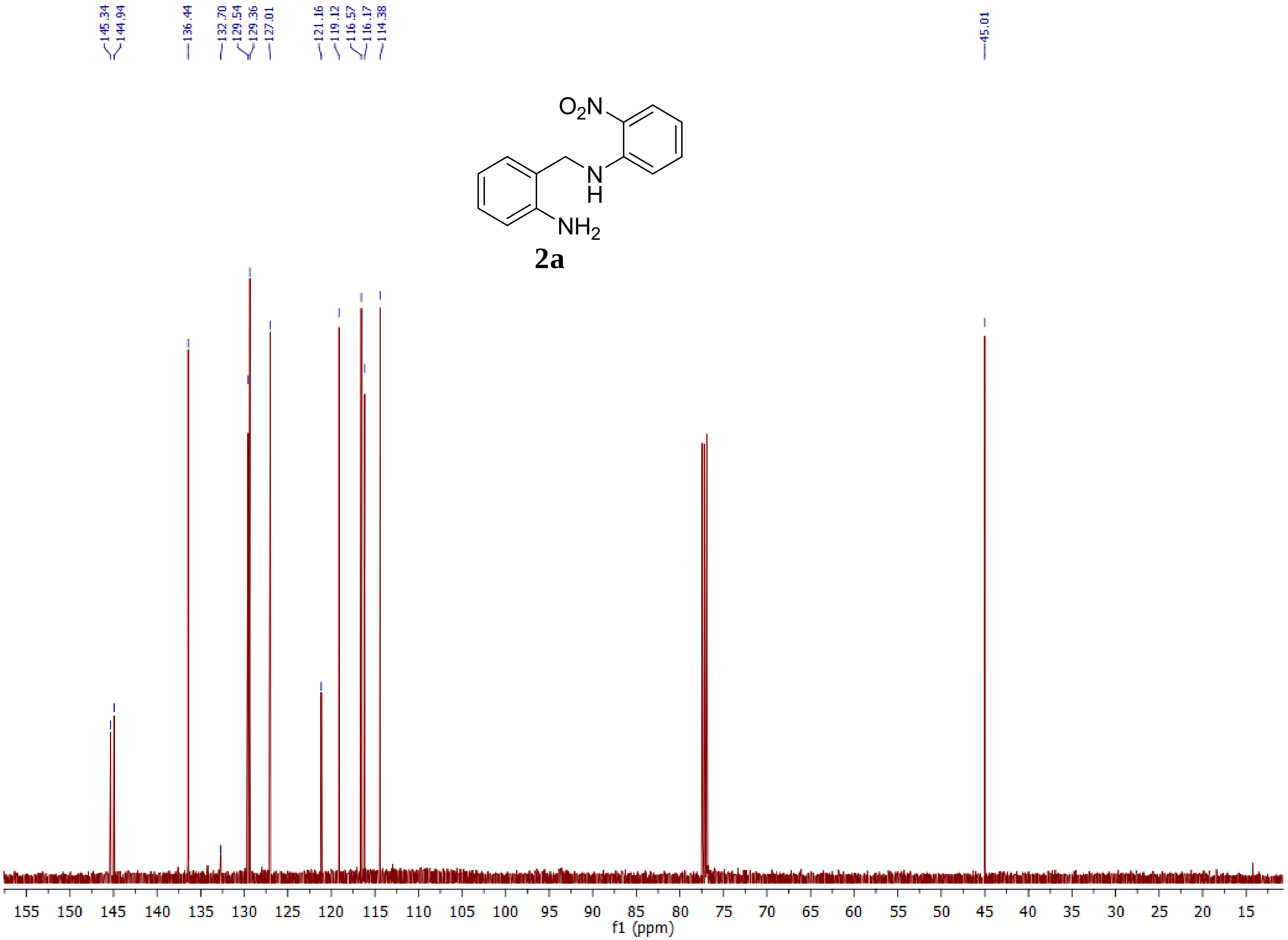

^{1}H NMR (500 MHz, $CDCl_3$) spectrum of compound **2b**

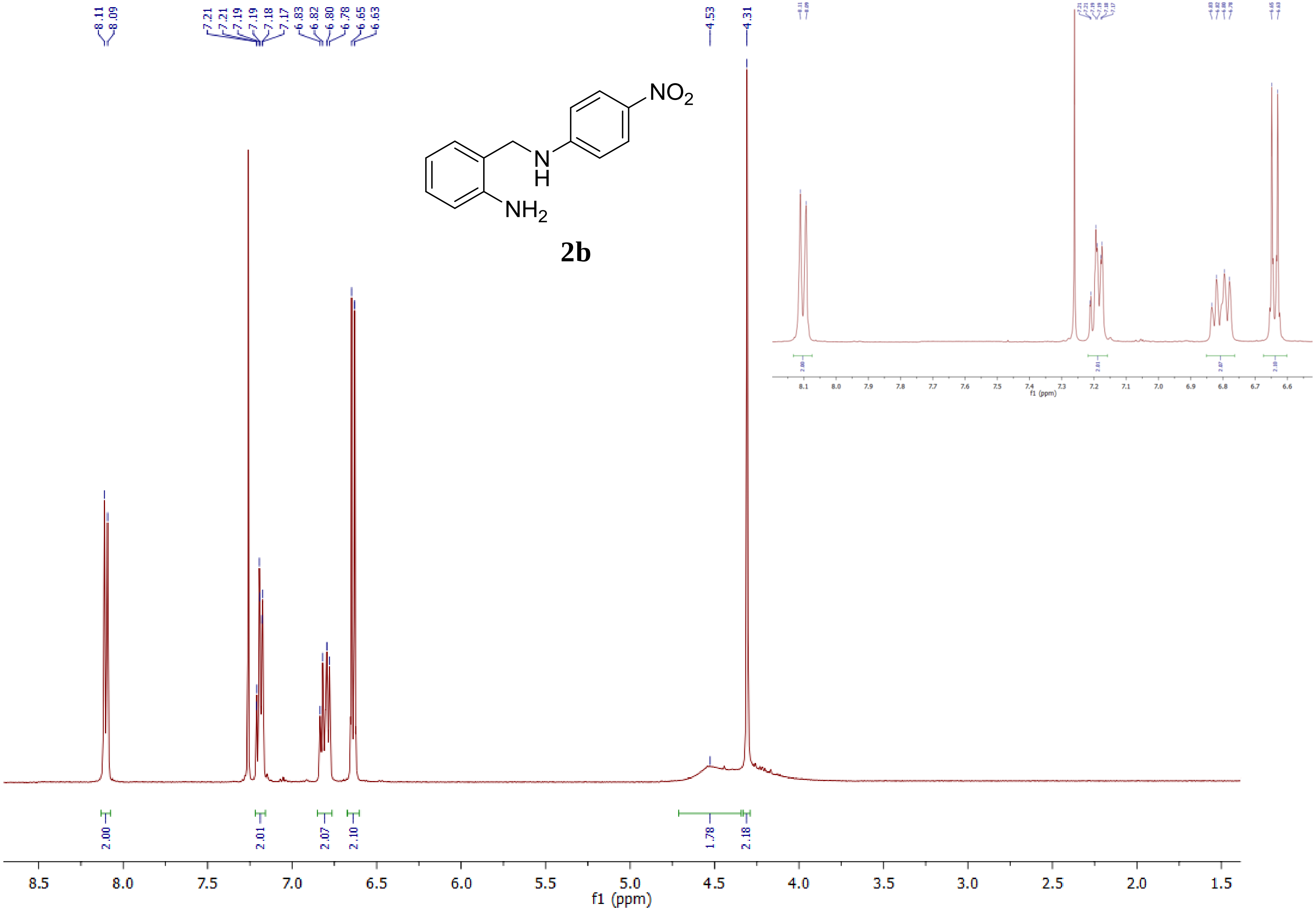

^{13}C NMR (126 MHz, $CDCl_3$) spectrum of compound **2b**

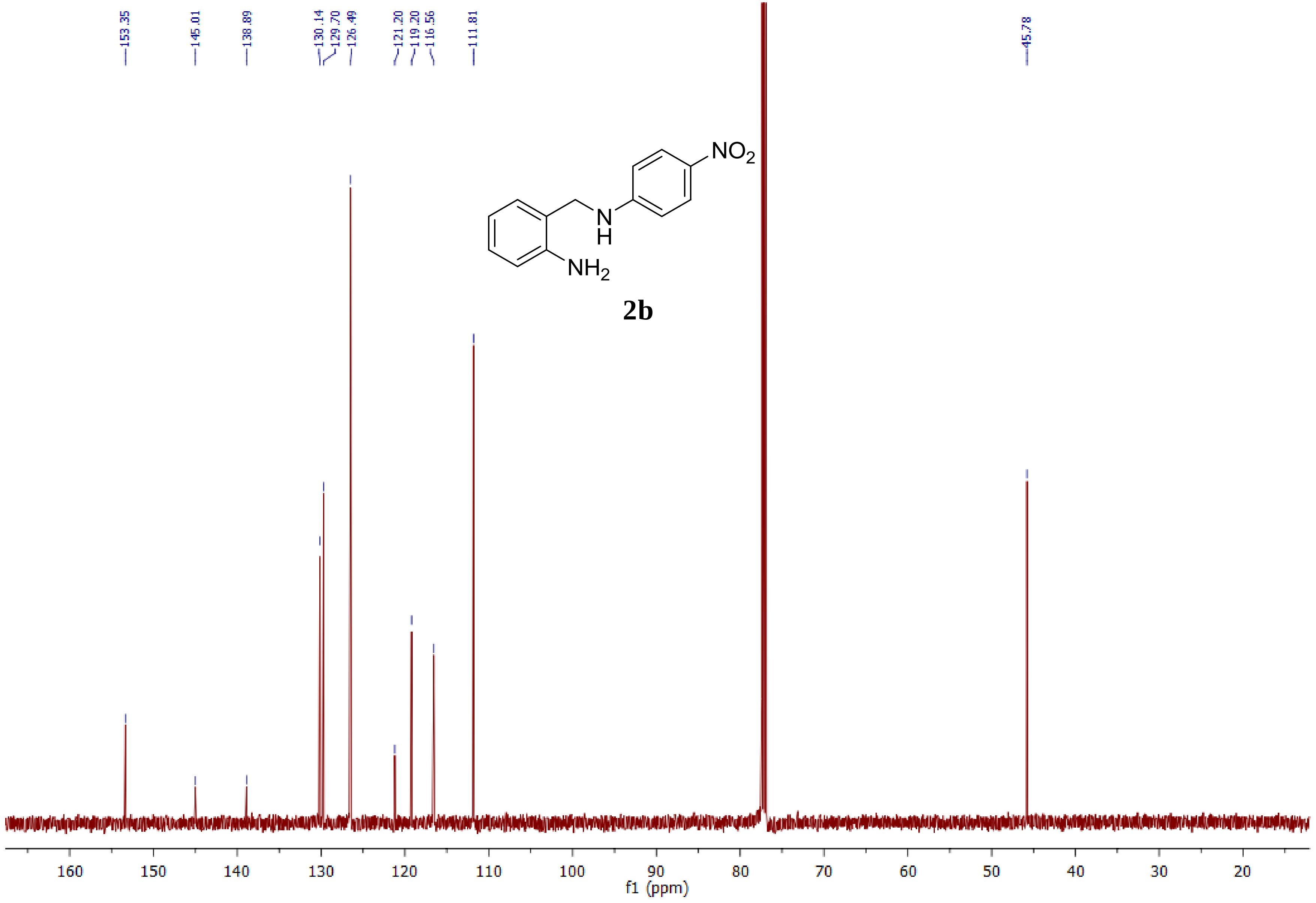

^{1}H NMR (500 MHz, $CDCl_3$) spectrum of compound **2c**

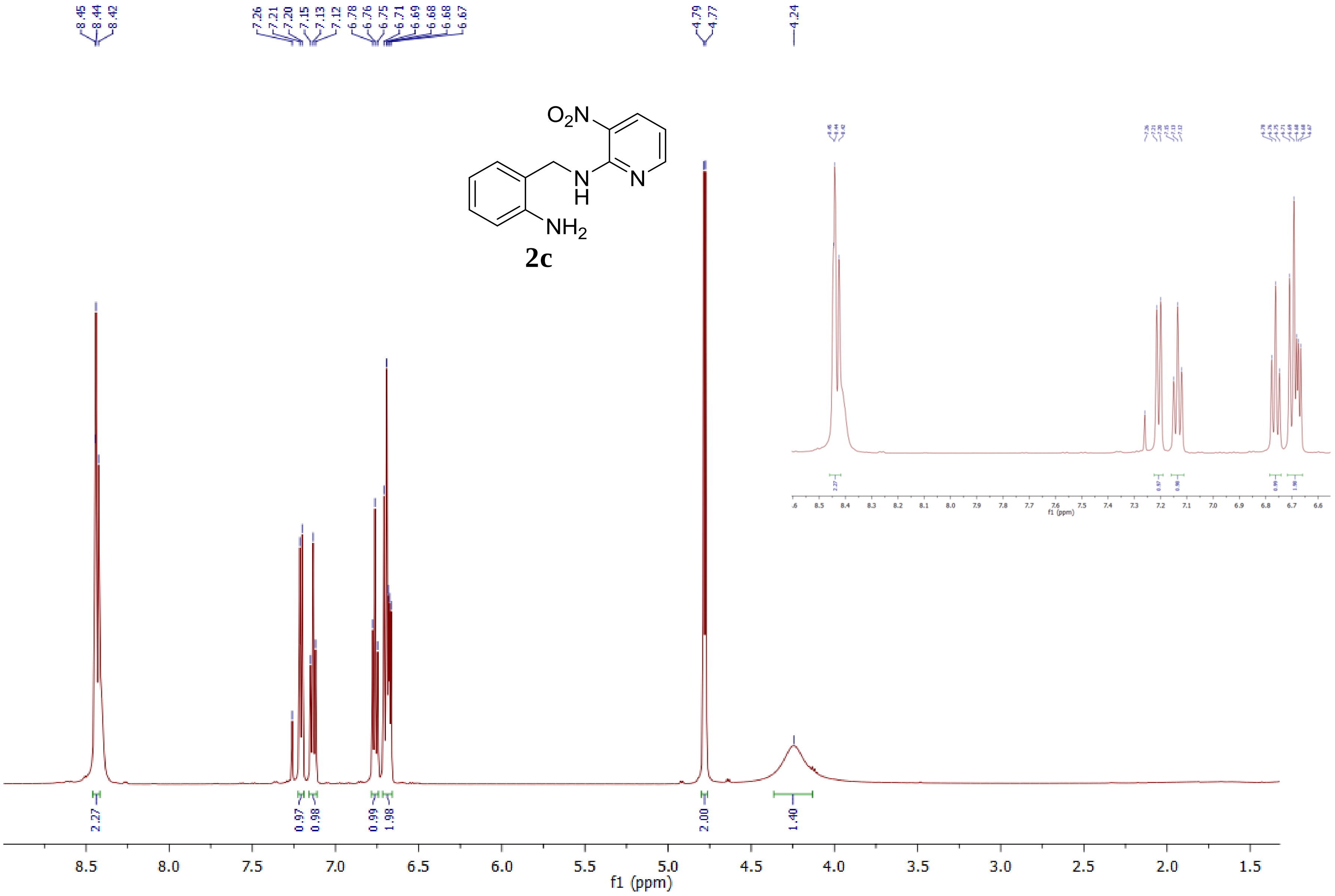

^{13}C NMR (126 MHz, $CDCl_3$) spectrum of compound **2c**

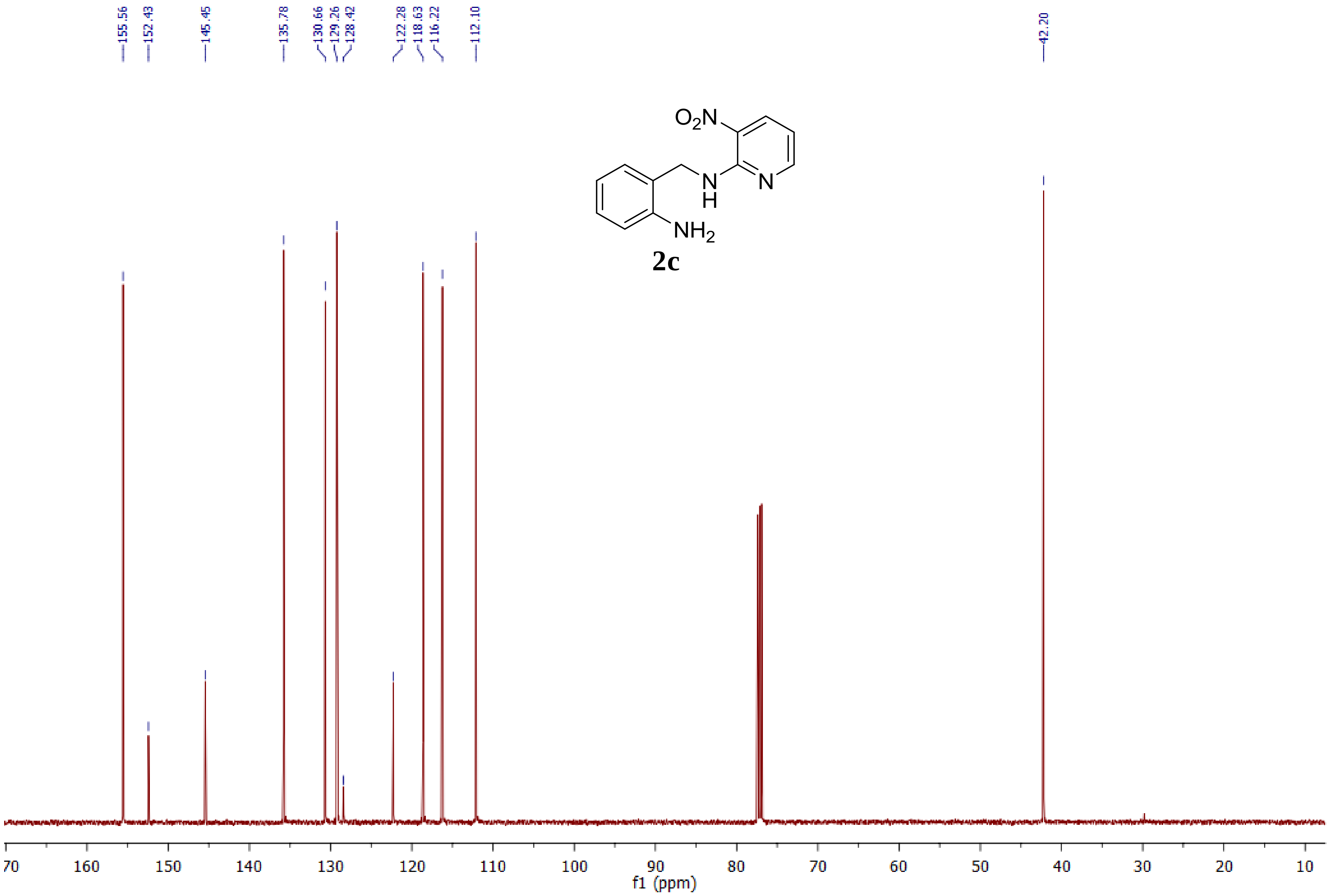

^{1}H NMR (500 MHz, $CDCl_3$) spectrum of compound **2d**

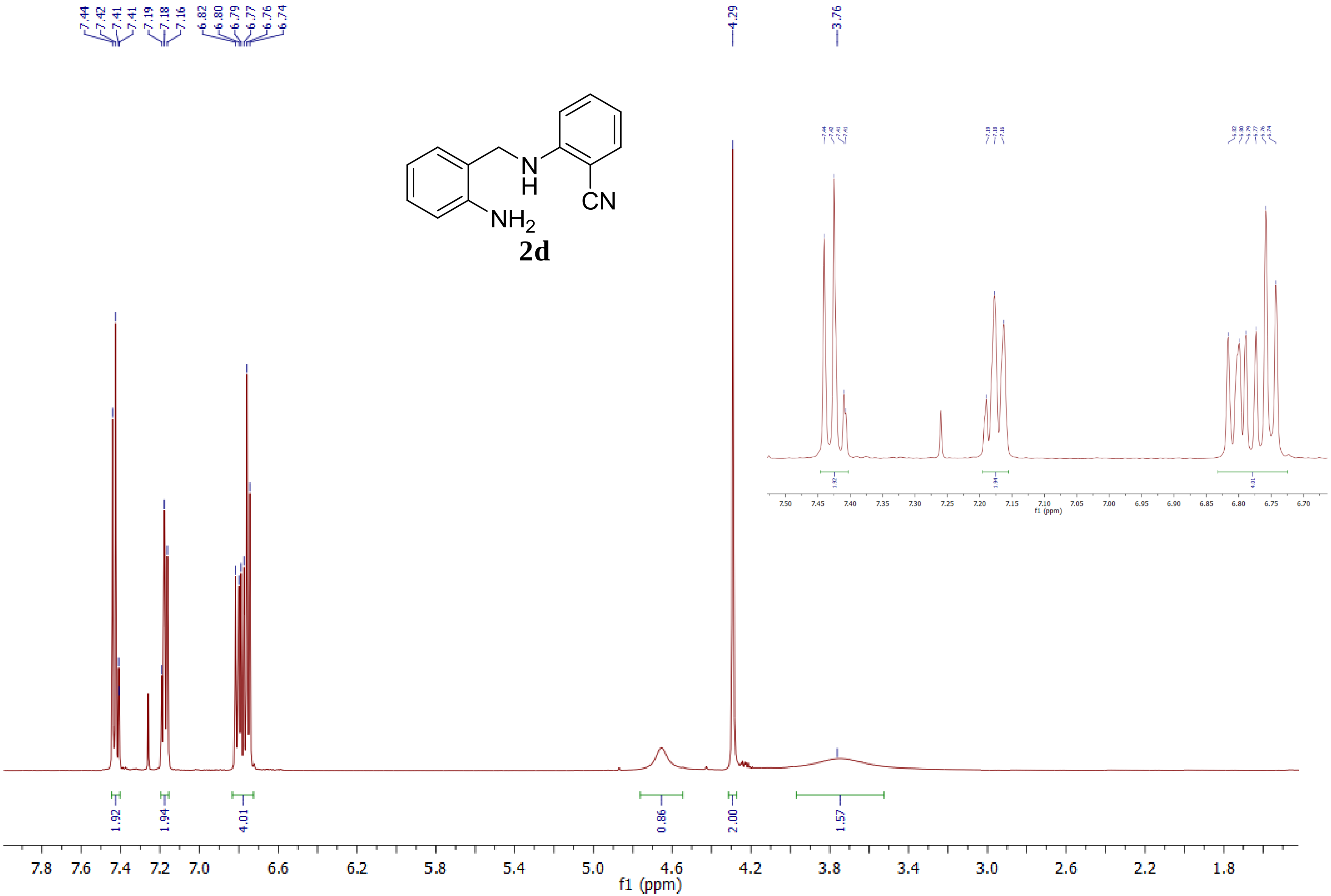

^{13}C NMR (126 MHz, $CDCl_3$) spectrum of compound **2d**

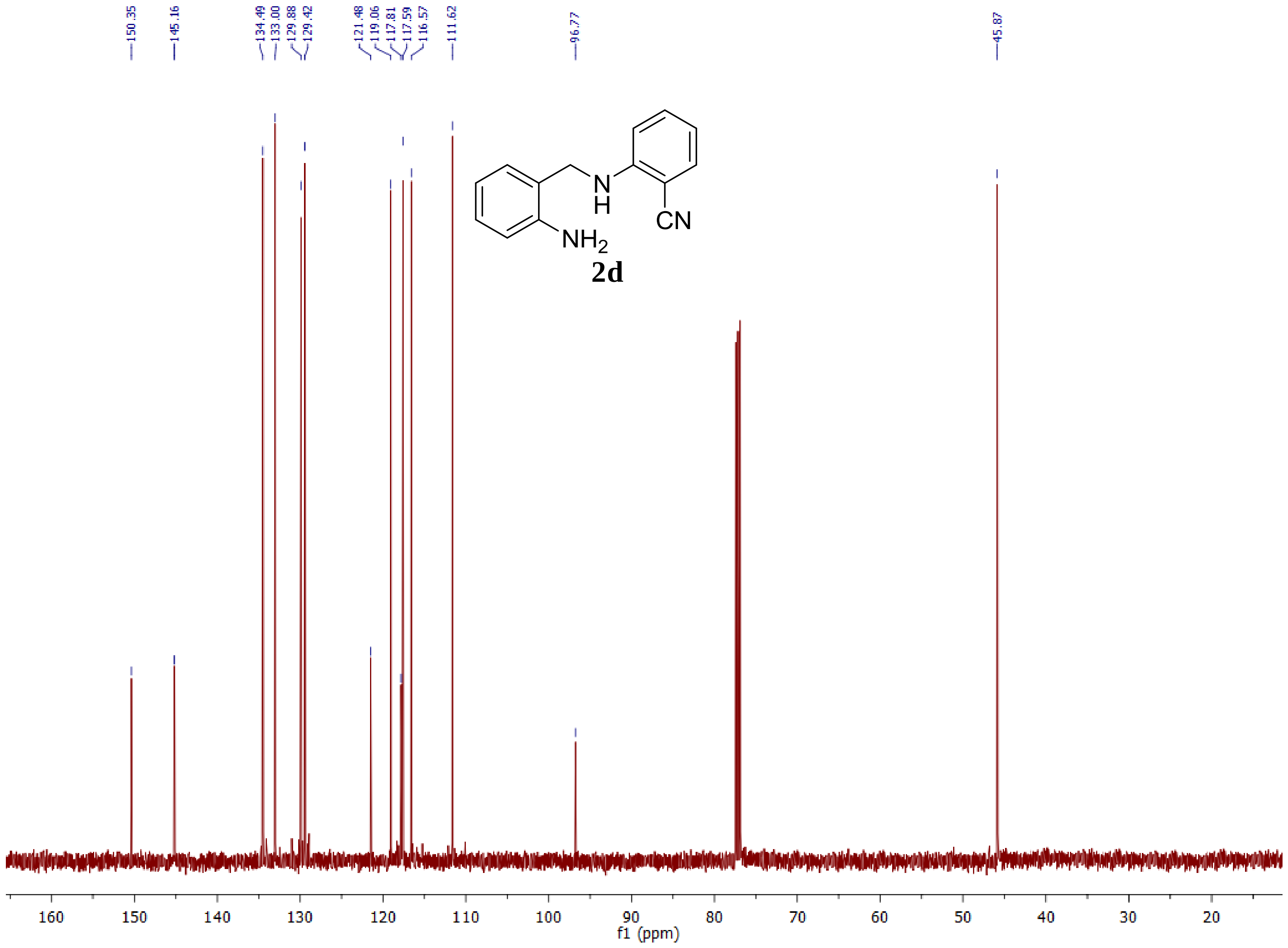

^{1}H NMR (500 MHz, $CDCl_3$) spectrum of compound **3a**

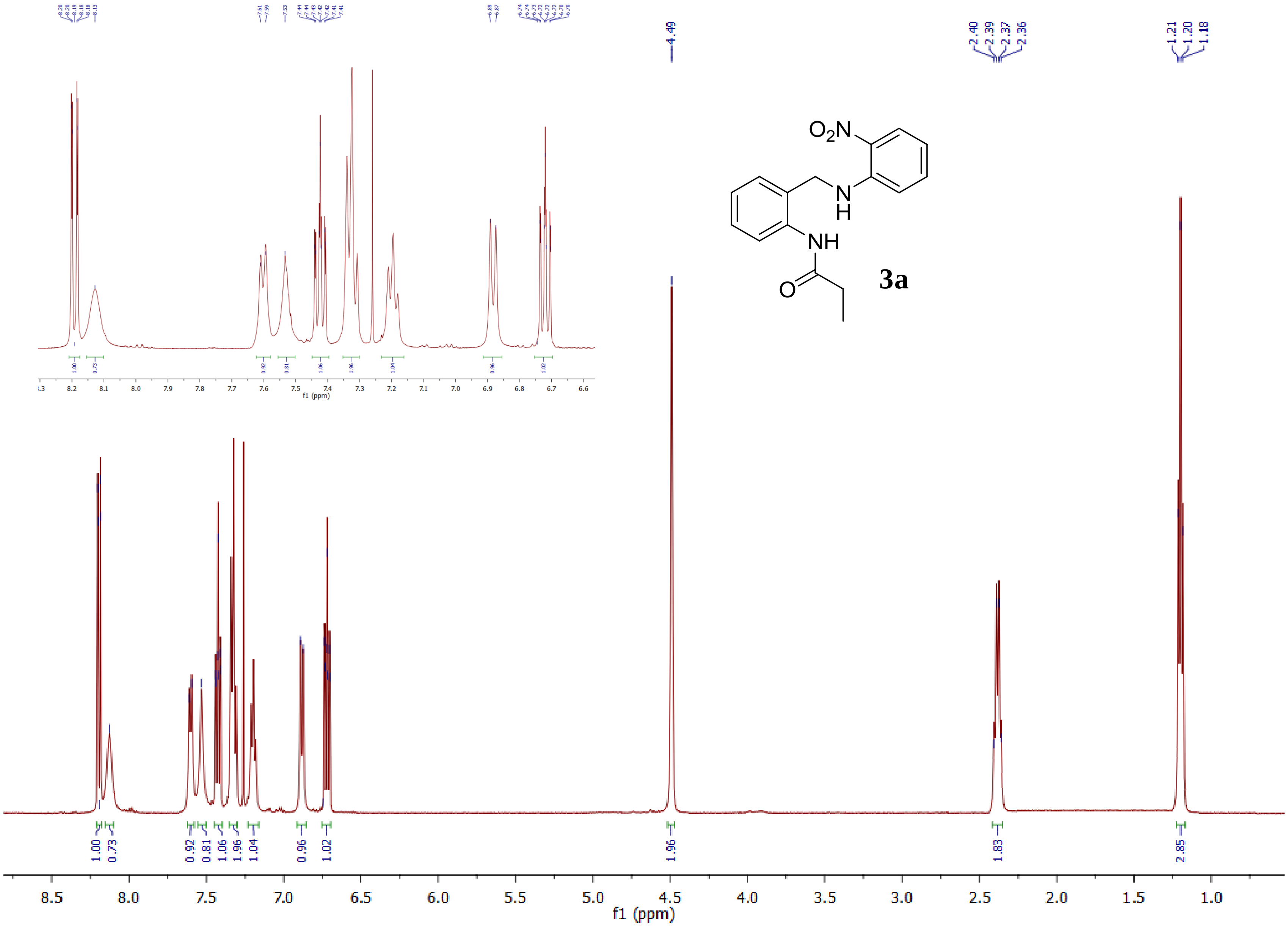

^{13}C NMR (126 MHz, $CDCl_3$) spectrum of compound **3a**

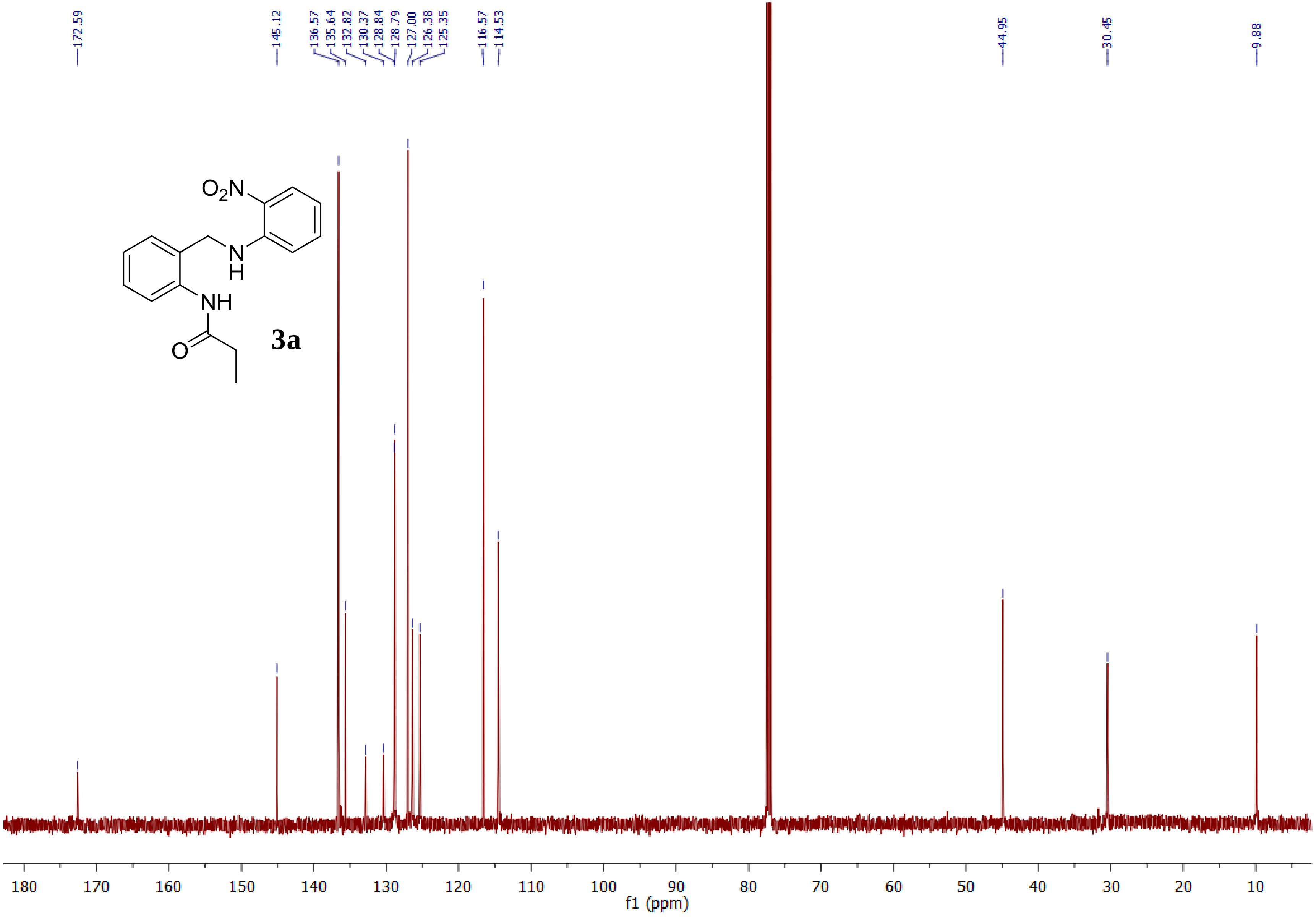

^{1}H NMR (300 MHz, $CDCl_3$) spectrum of compound **3b**

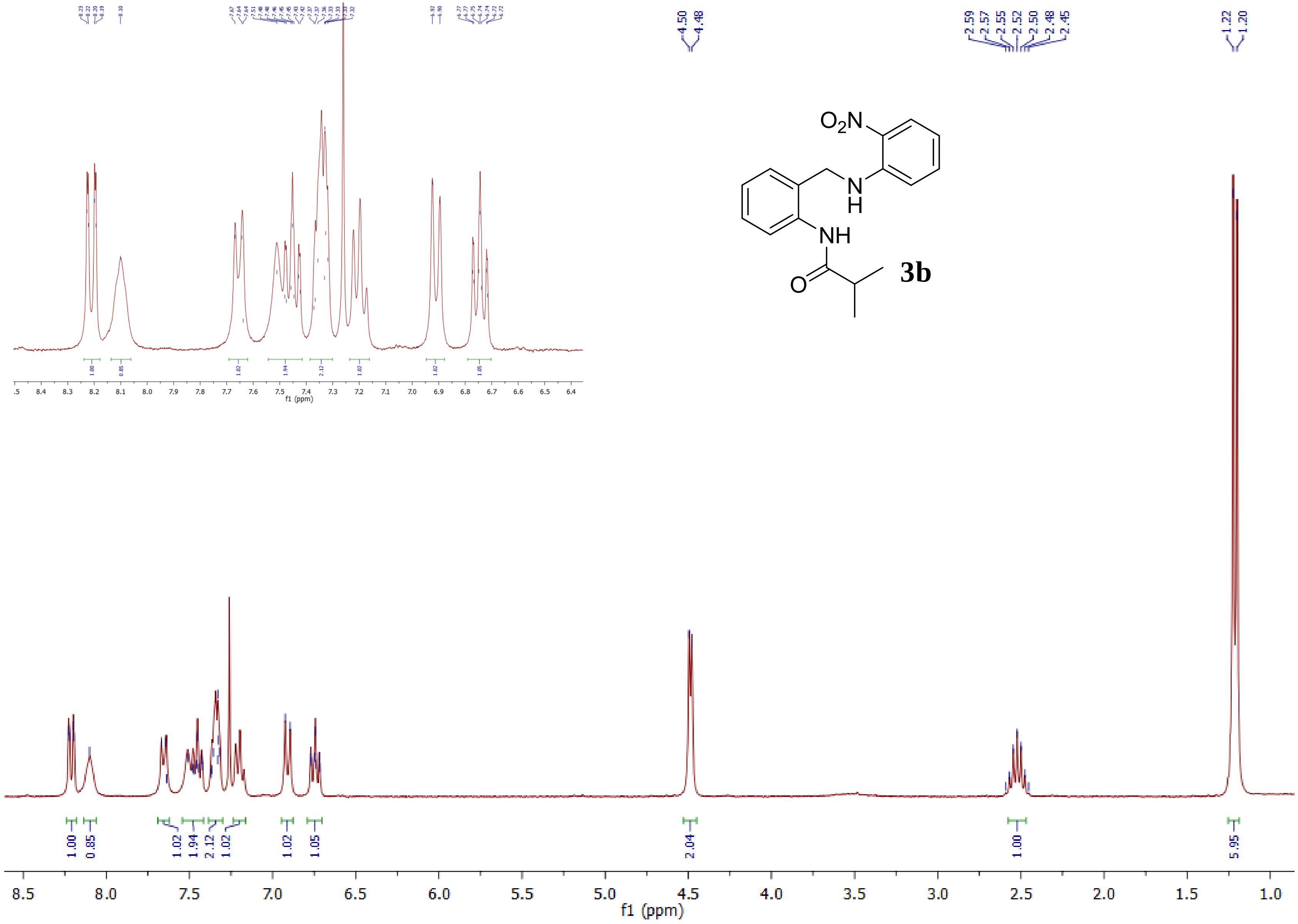

^{13}C NMR (75 MHz, $CDCl_3$) spectrum of compound **3b**

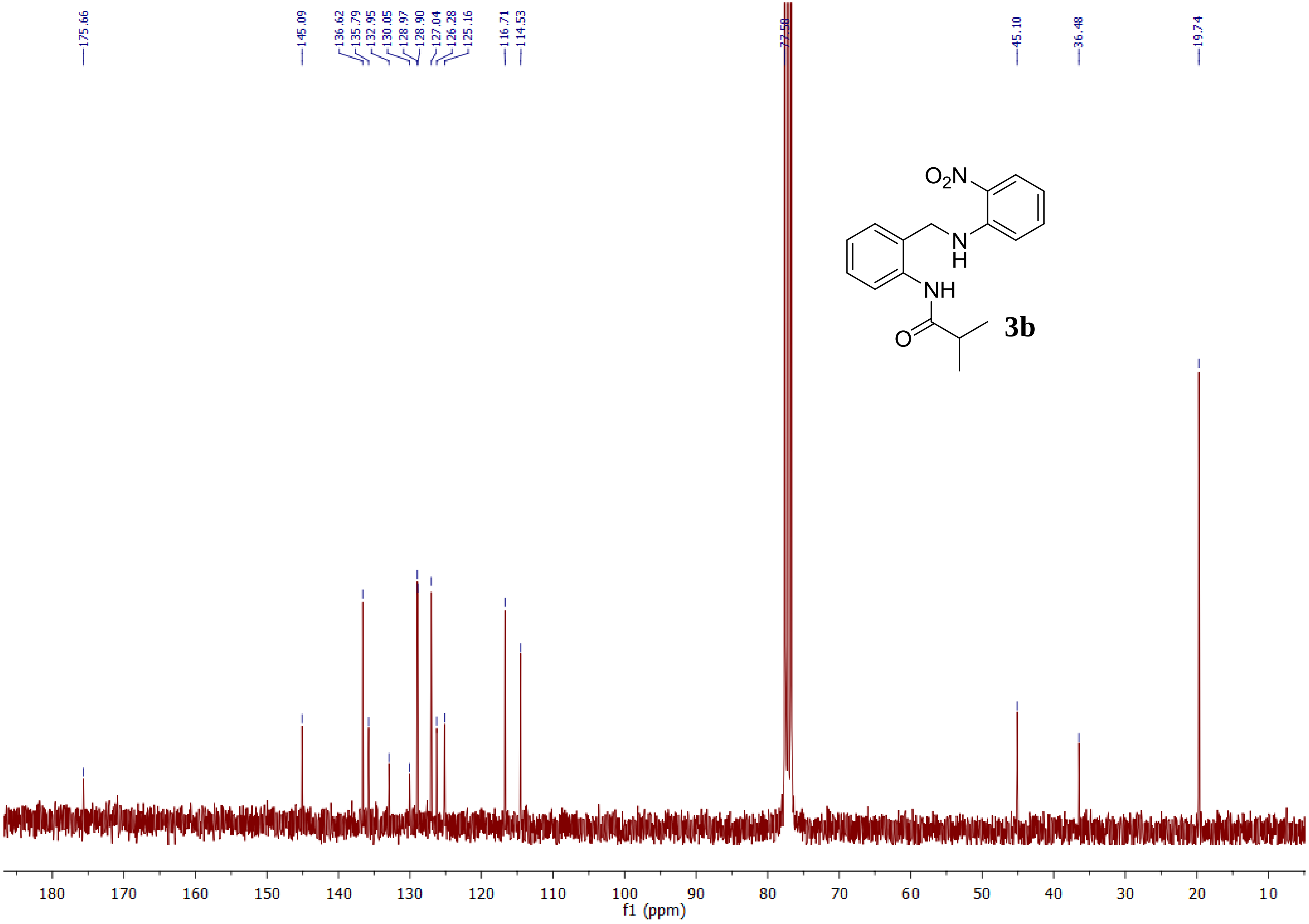

^{1}H NMR (600 MHz, $CDCl_3$) spectrum of compound **3c**

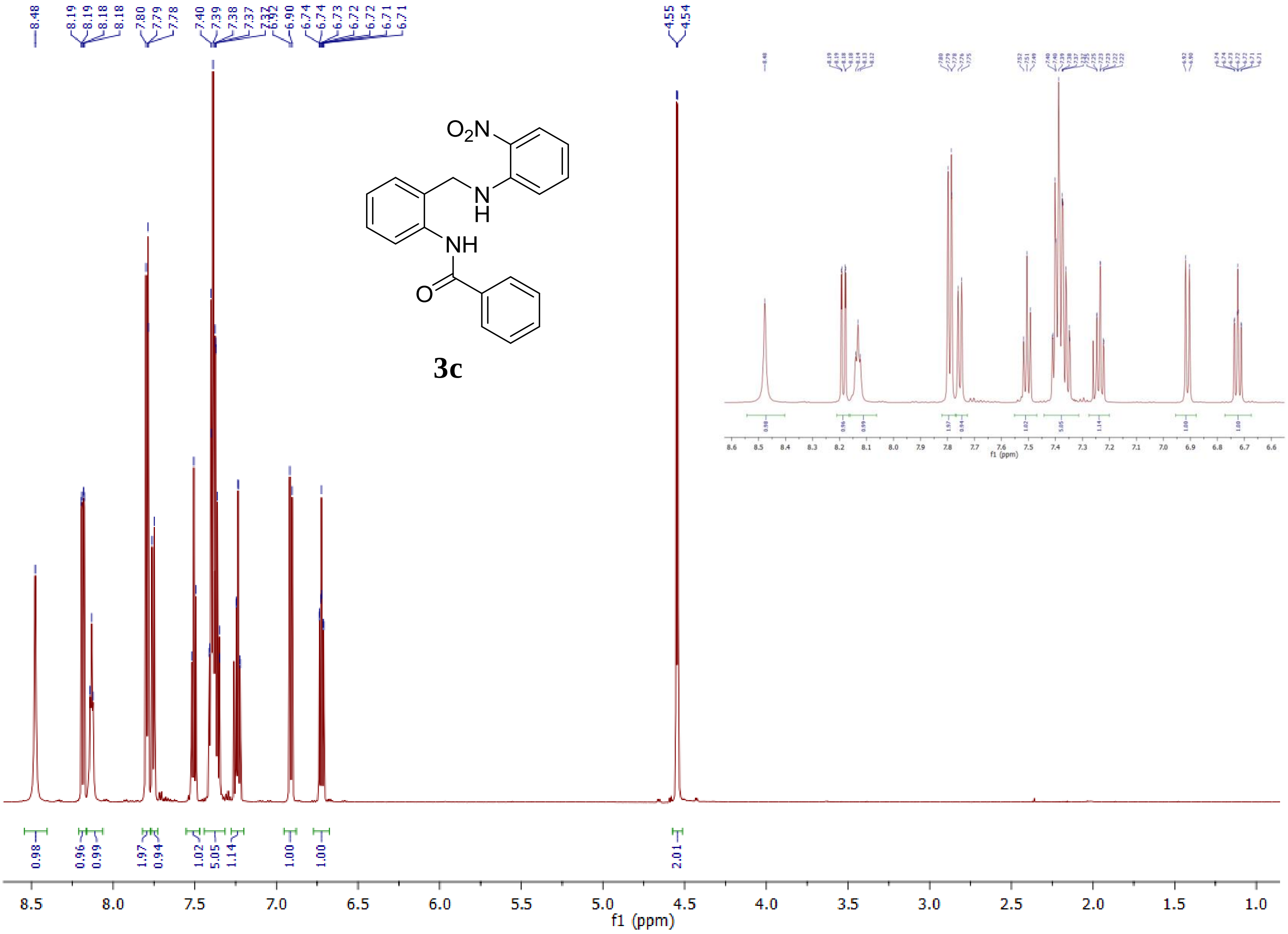

^{13}C NMR (151 MHz, $CDCl_3$) spectrum of compound **3c**

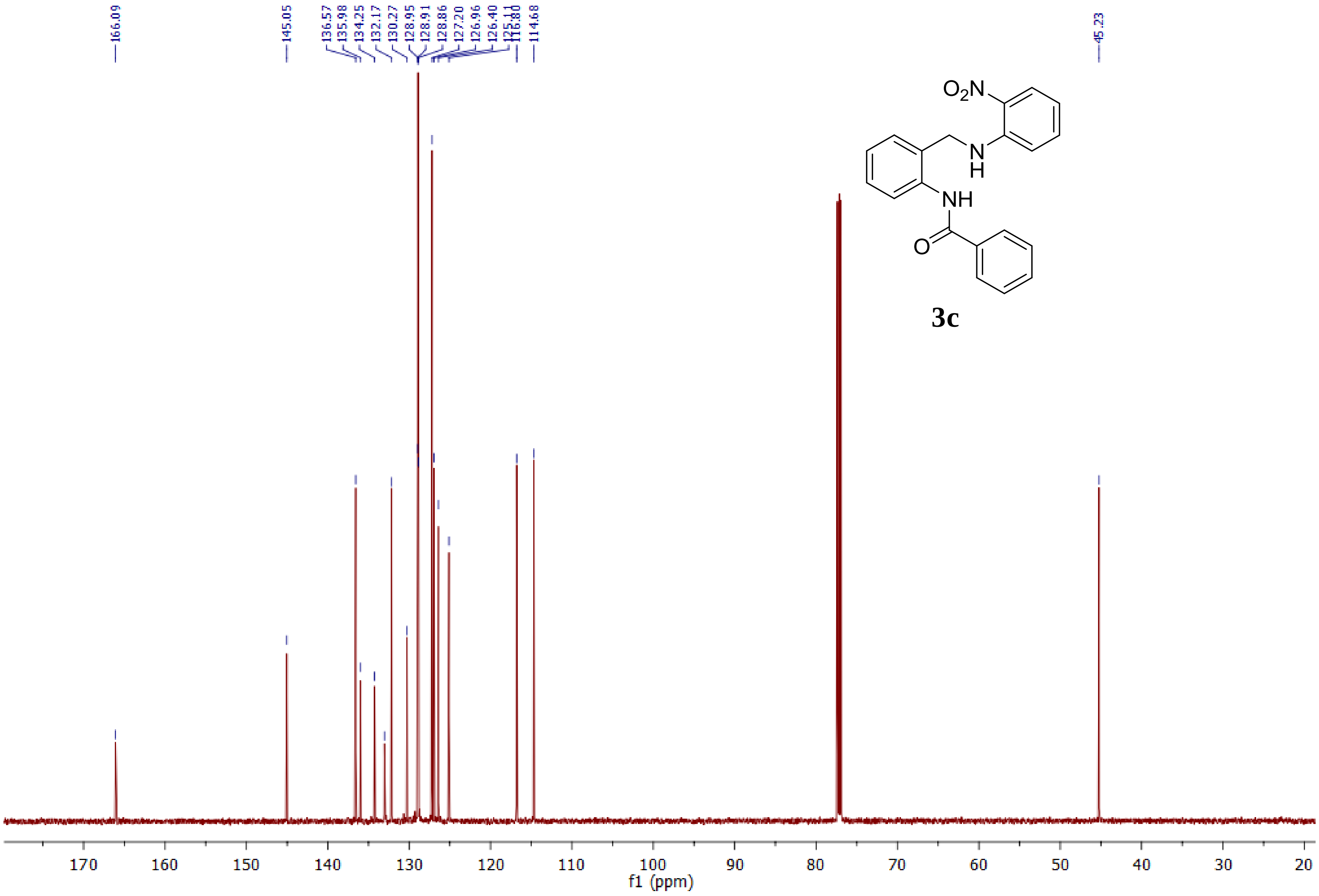

^{1}H NMR (500 MHz, $CDCl_3$) spectrum of compound **3d**

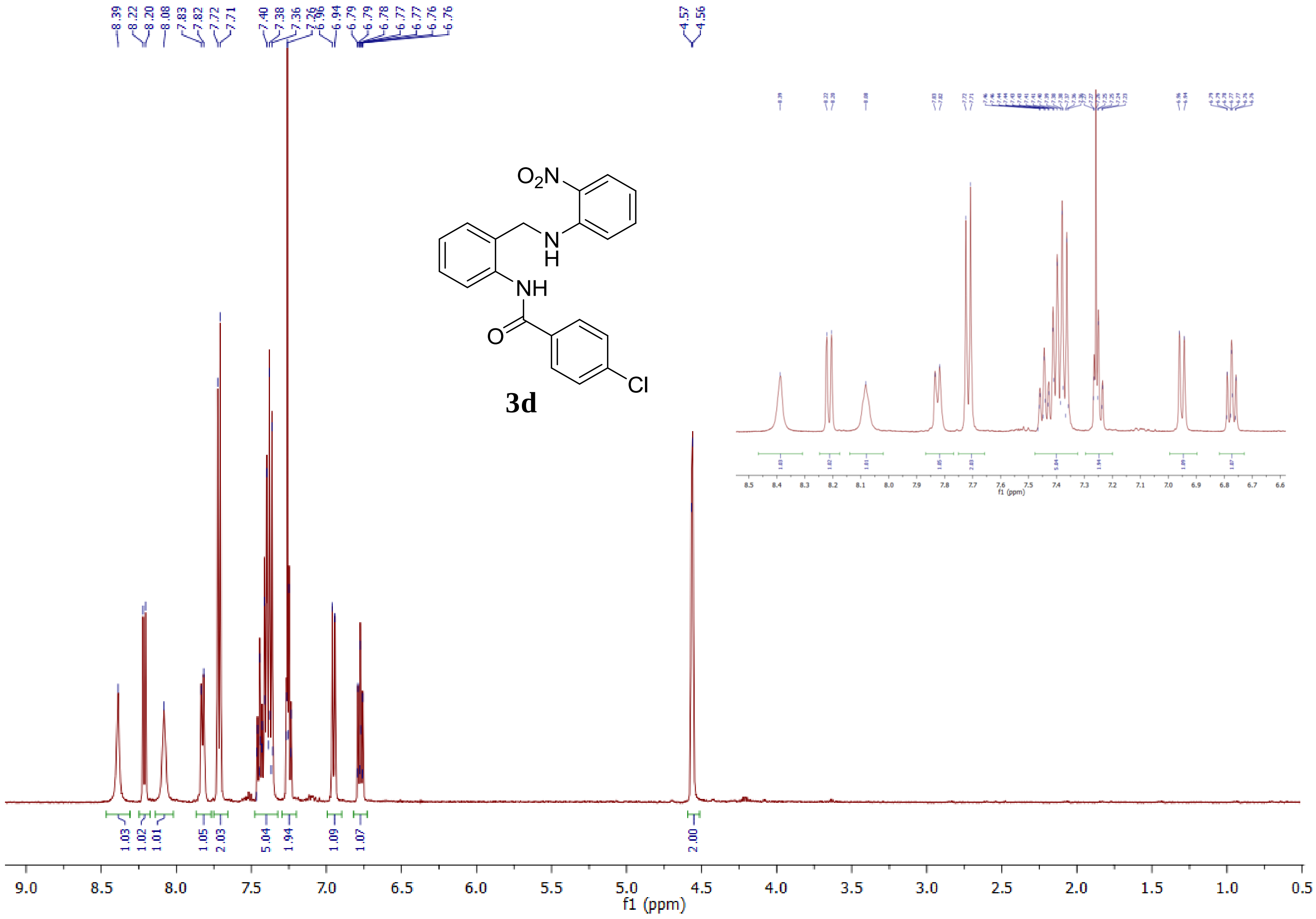

^{13}C NMR (126 MHz, $CDCl_3$) spectrum of compound **3d**

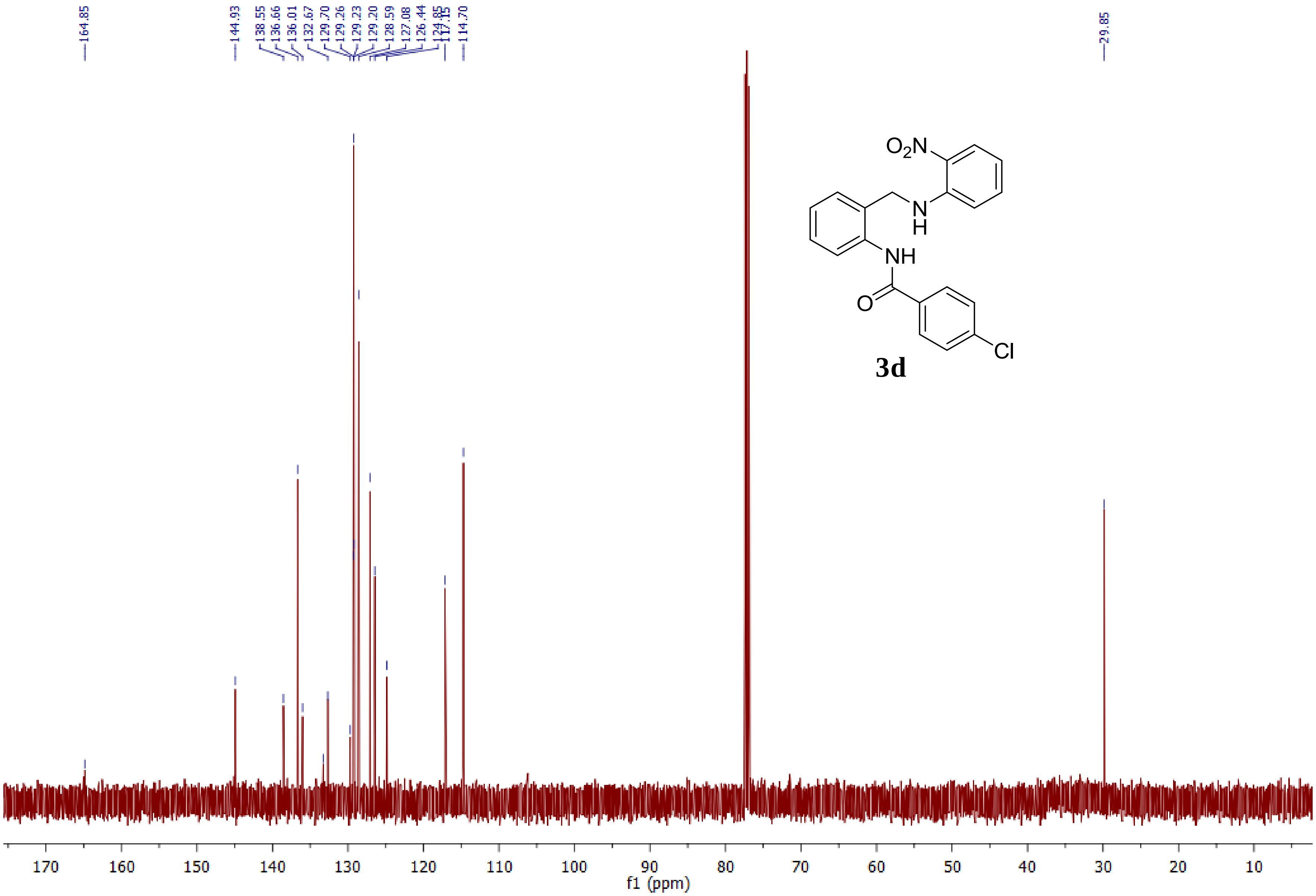

^{1}H NMR (500 MHz, $CDCl_3$) spectrum of compound **3e**

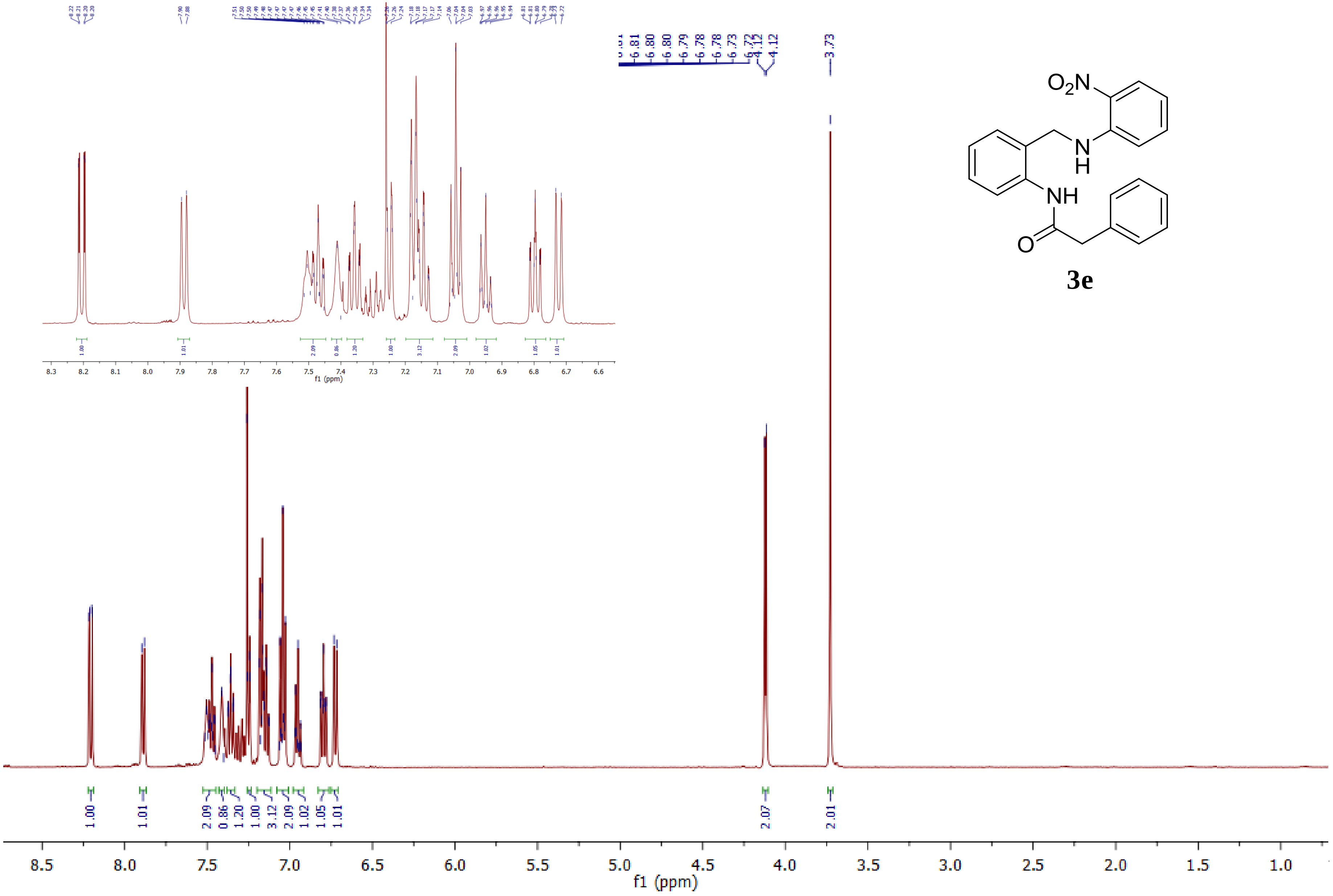

3e

^{13}C NMR (126 MHz, $CDCl_3$) spectrum of compound **3e**

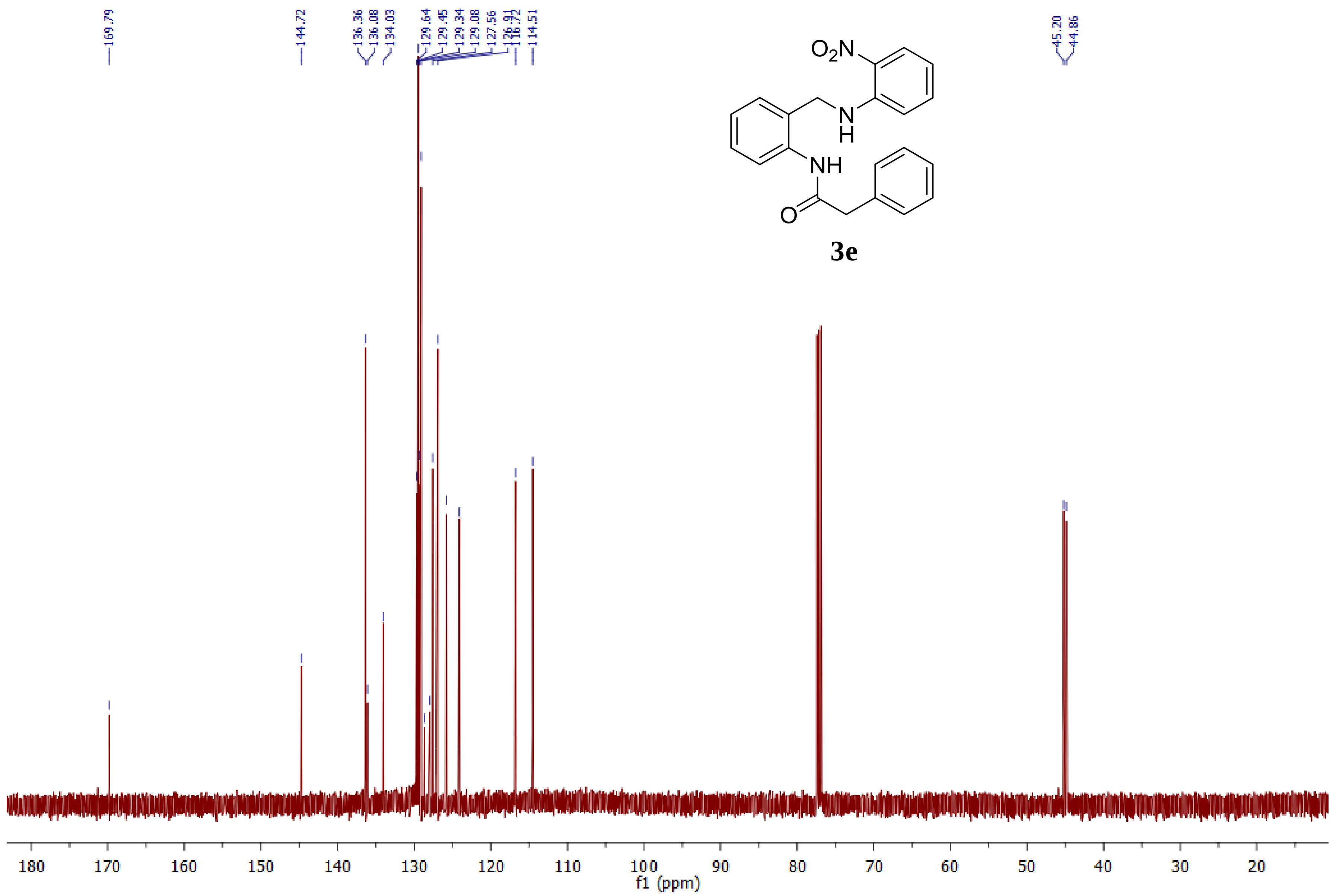

^{1}H NMR (600 MHz, $CDCl_3$) spectrum of compound **3f**

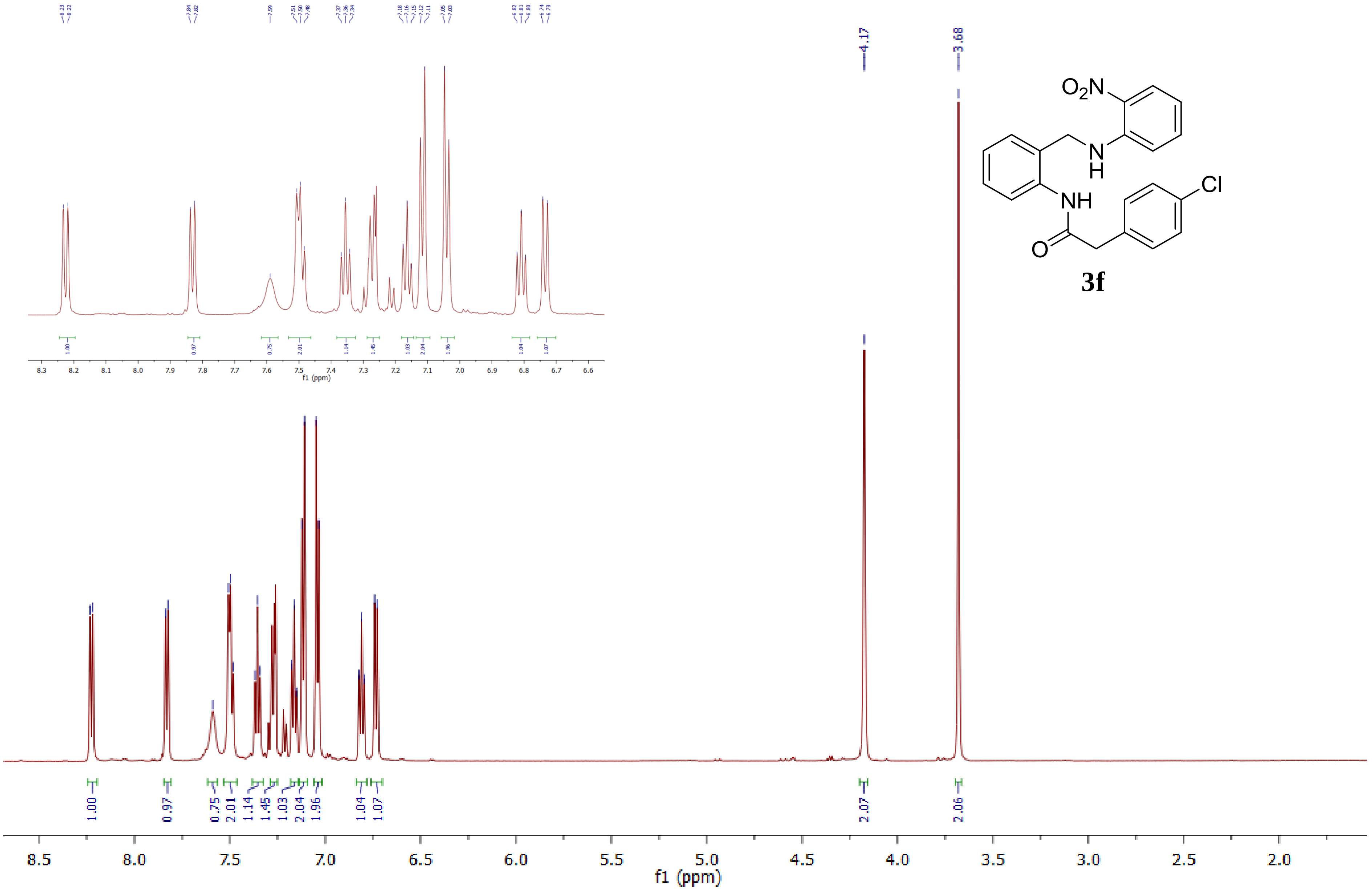

^{13}C NMR (151 MHz, $CDCl_3$) spectrum of compound **3f**

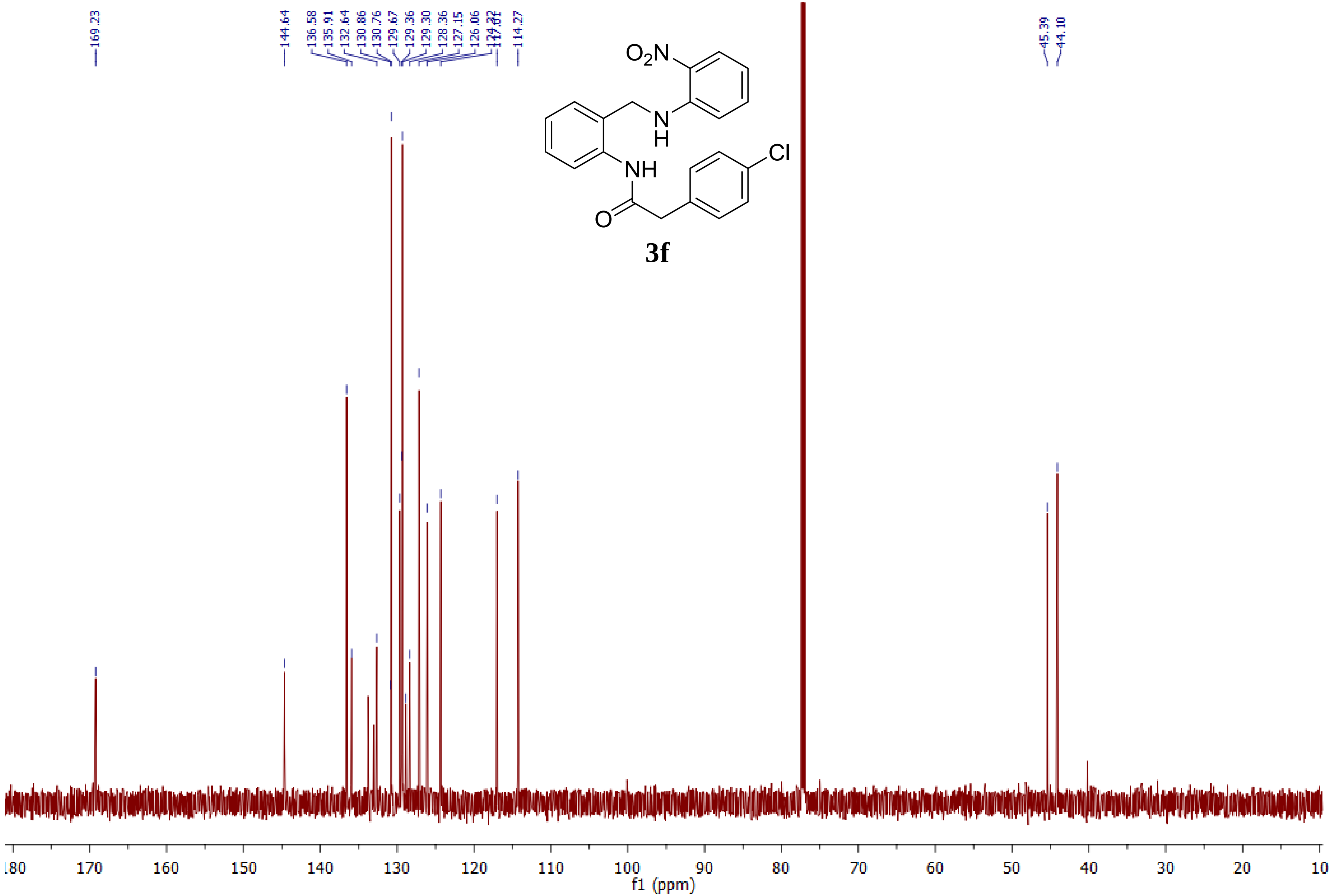

^{1}H NMR (600 MHz, DMSO-*d6*) spectrum of compound **3g**

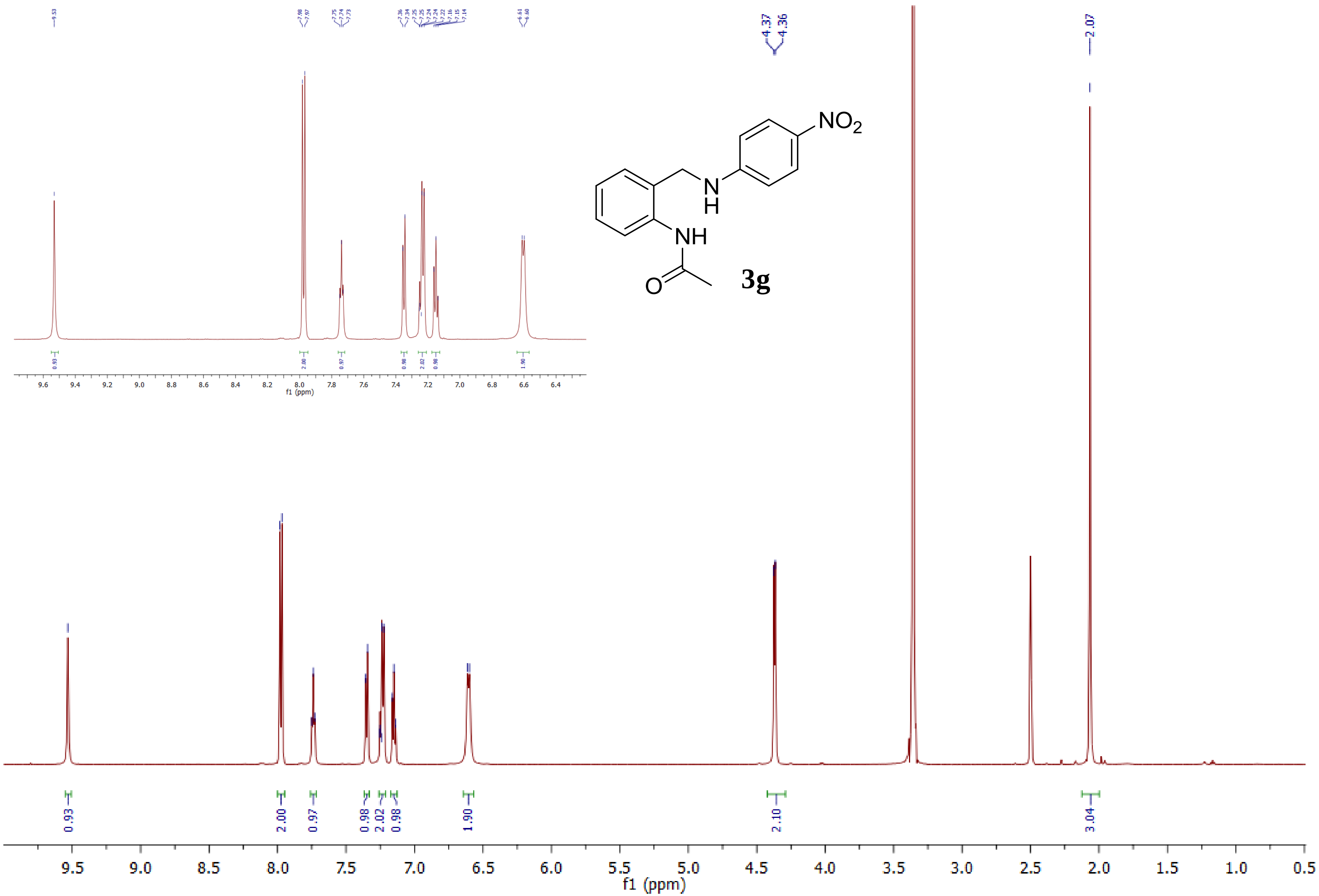

^{13}C NMR (151 MHz, DMSO-*d*6) spectrum of compound **3g**

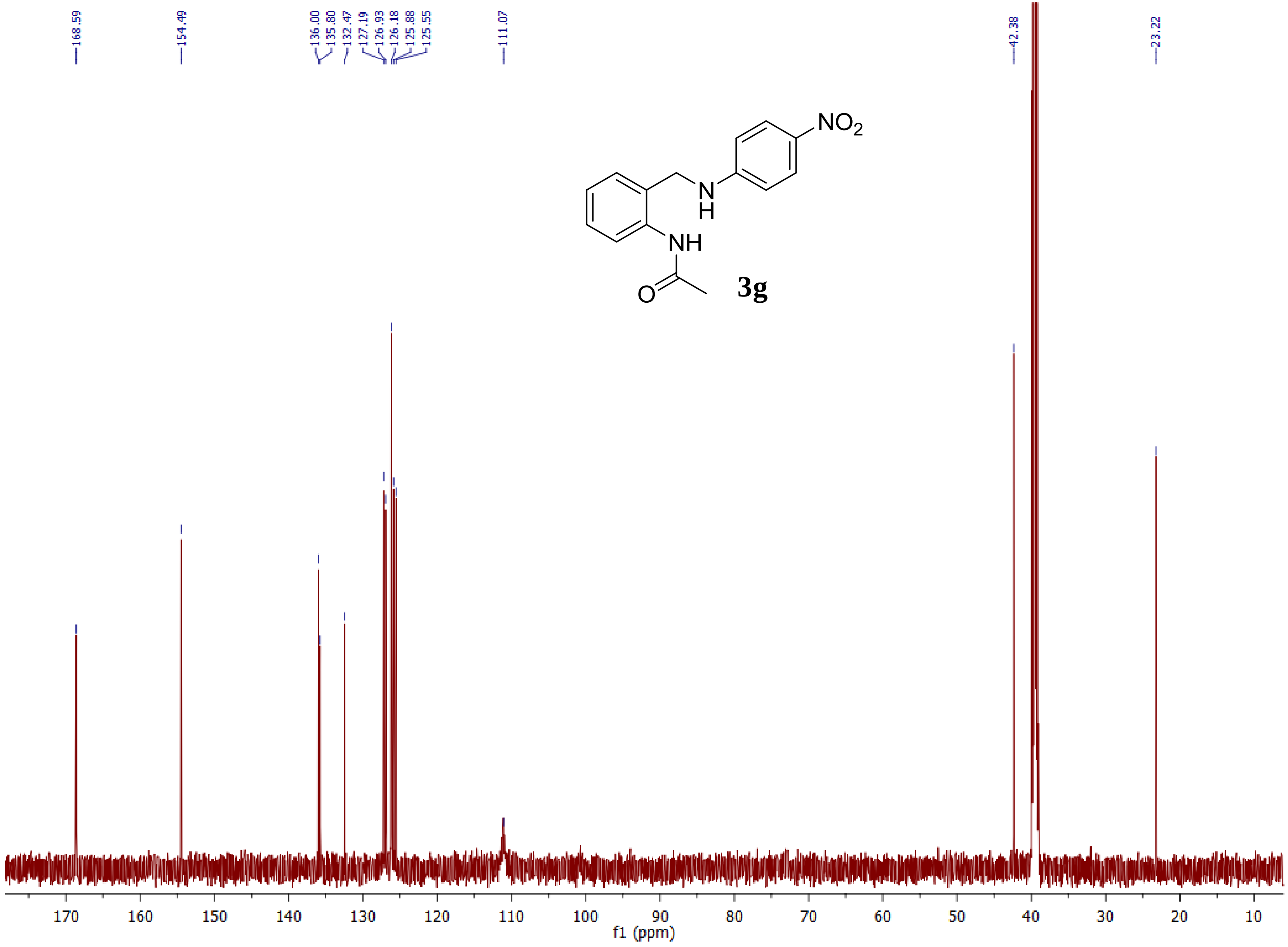

^{1}H NMR (500 MHz, DMSO-*d*6) spectrum of compound **3h**

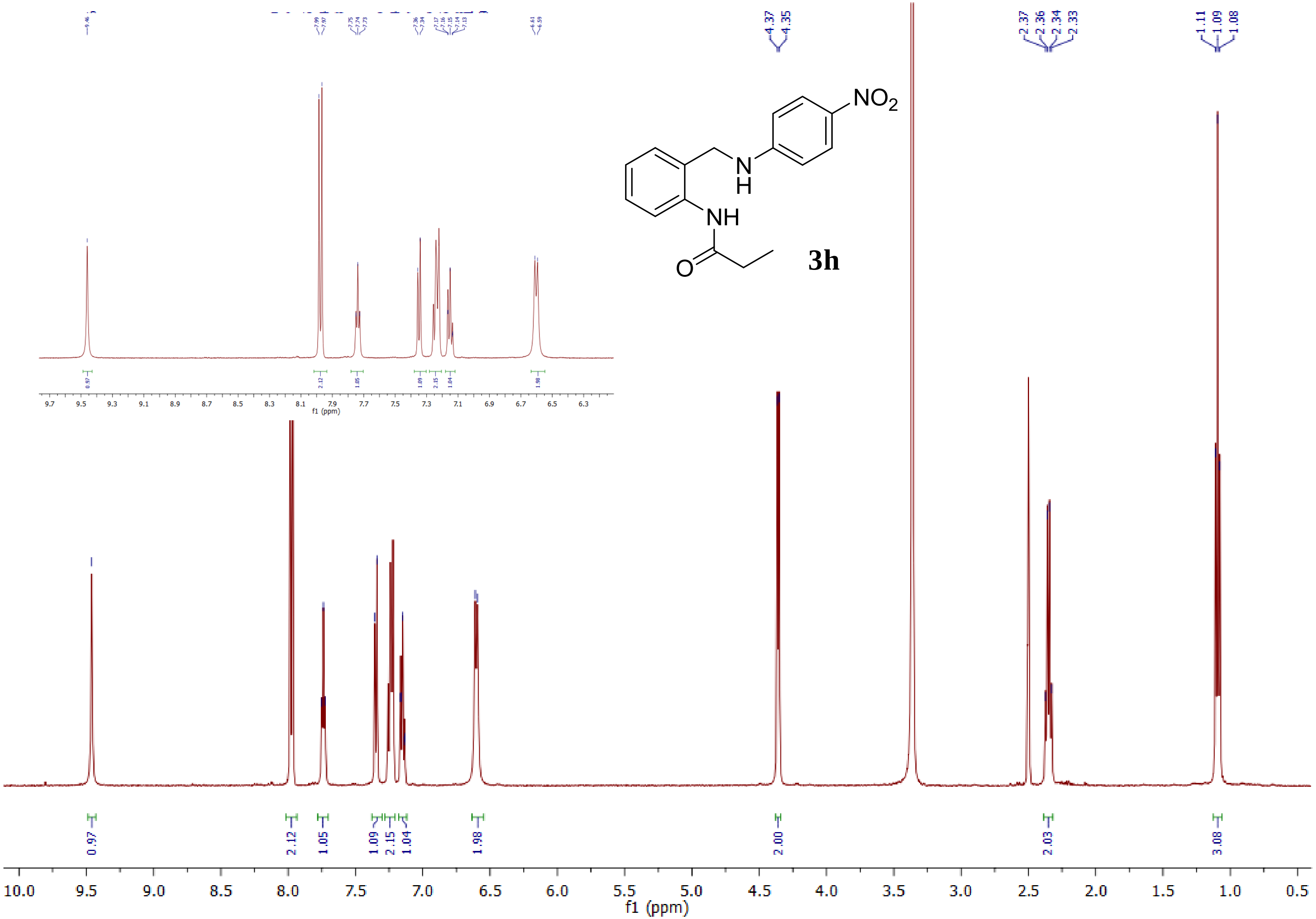

^{13}C NMR (126 MHz, DMSO-*d*6) spectrum of compound **3h**

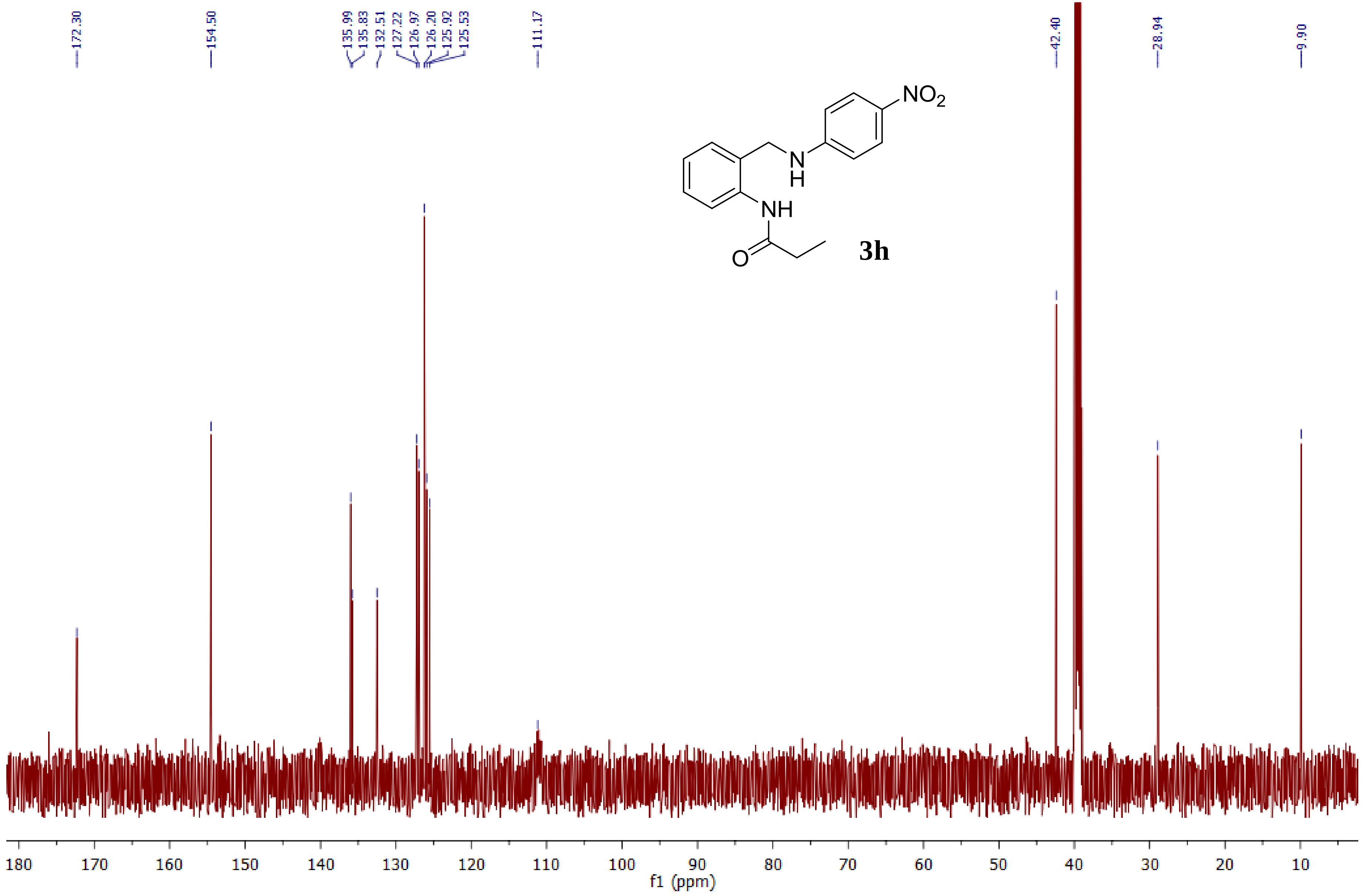

^{1}H NMR (500 MHz, DMSO-$d6$) spectrum of compound **3i**

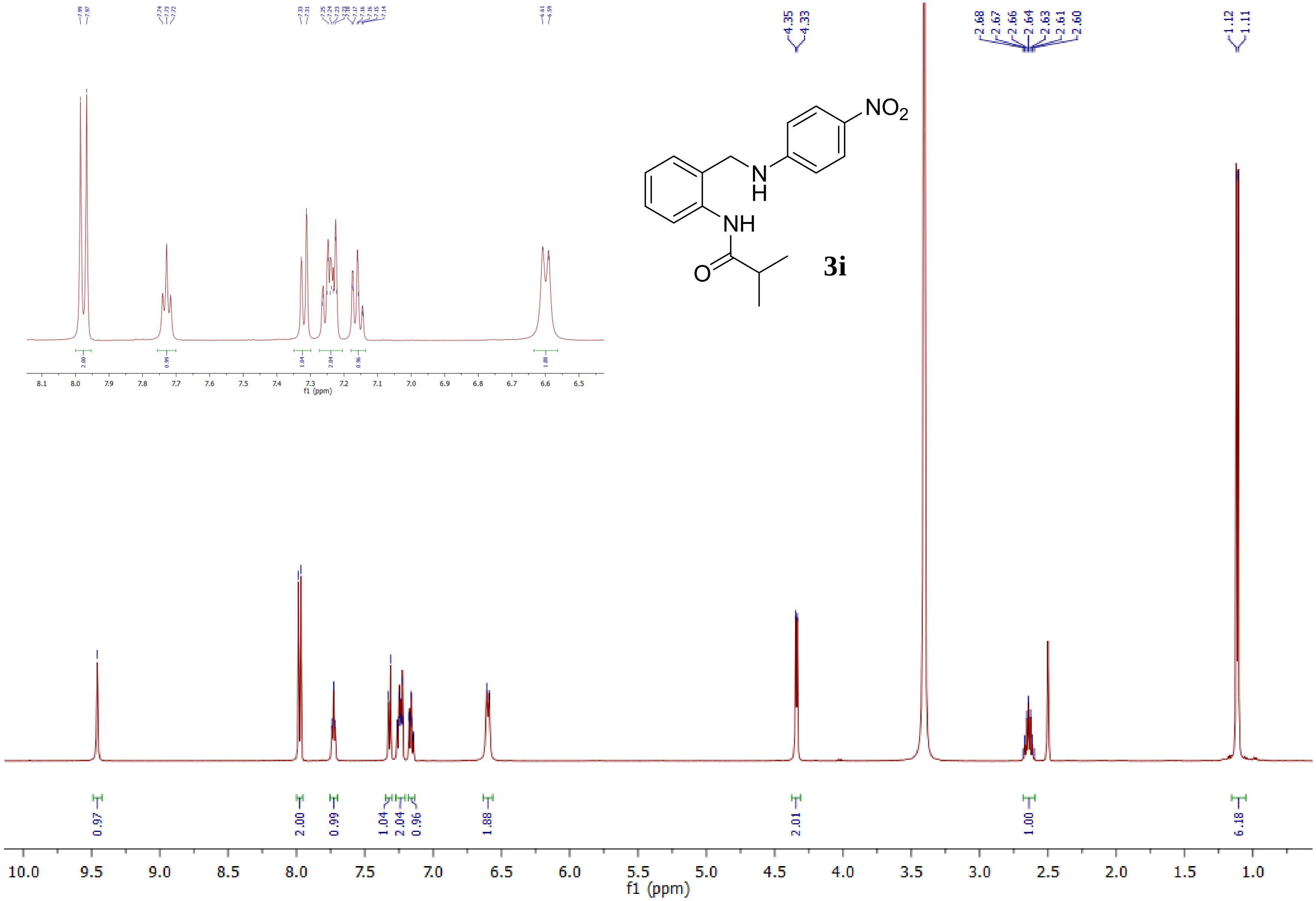

^{13}C NMR (126 MHz, DMSO-*d*6) spectrum of compound **3i**

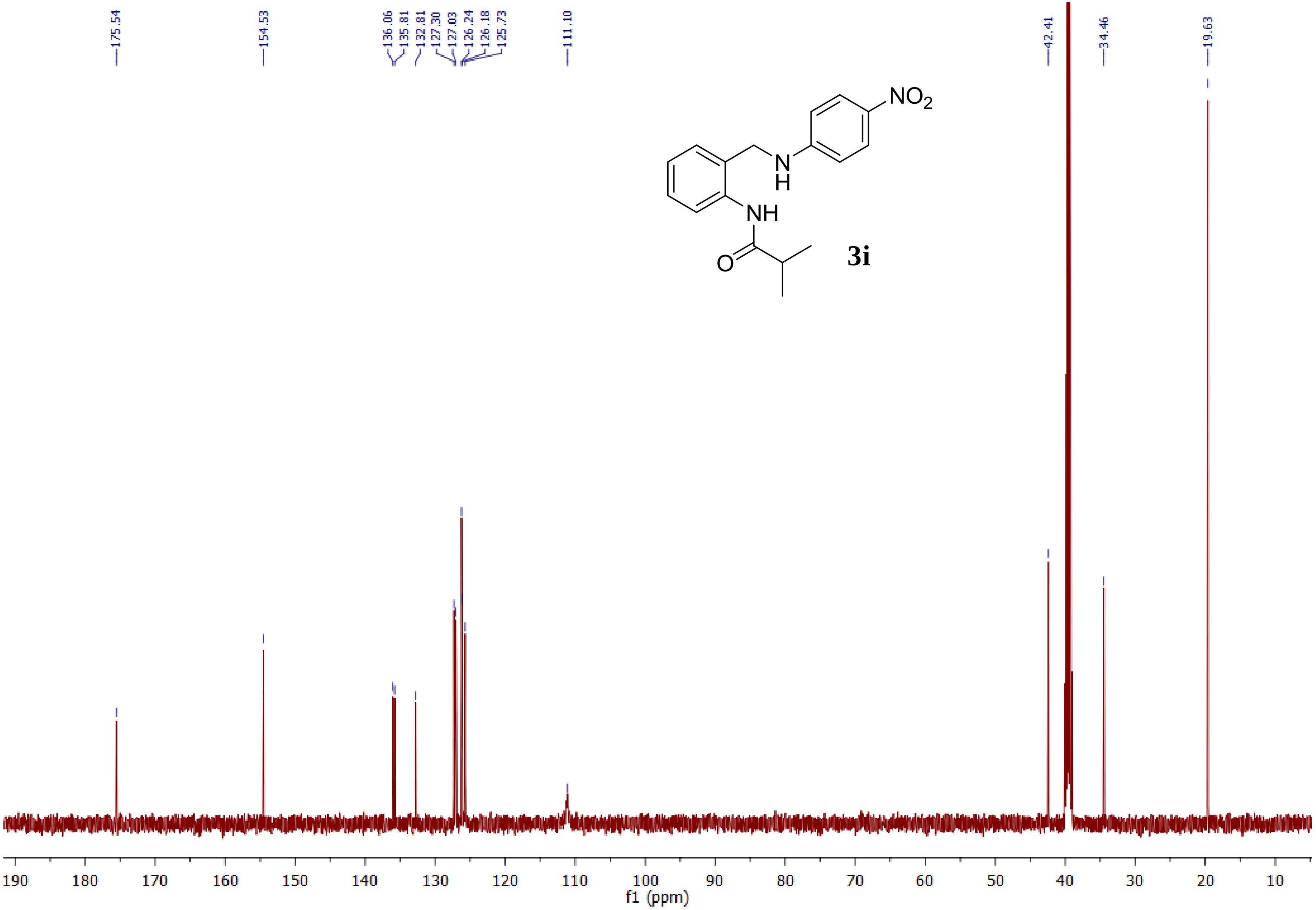

^{1}H NMR (300 MHz, DMSO-*d*6) spectrum of compound **3j**

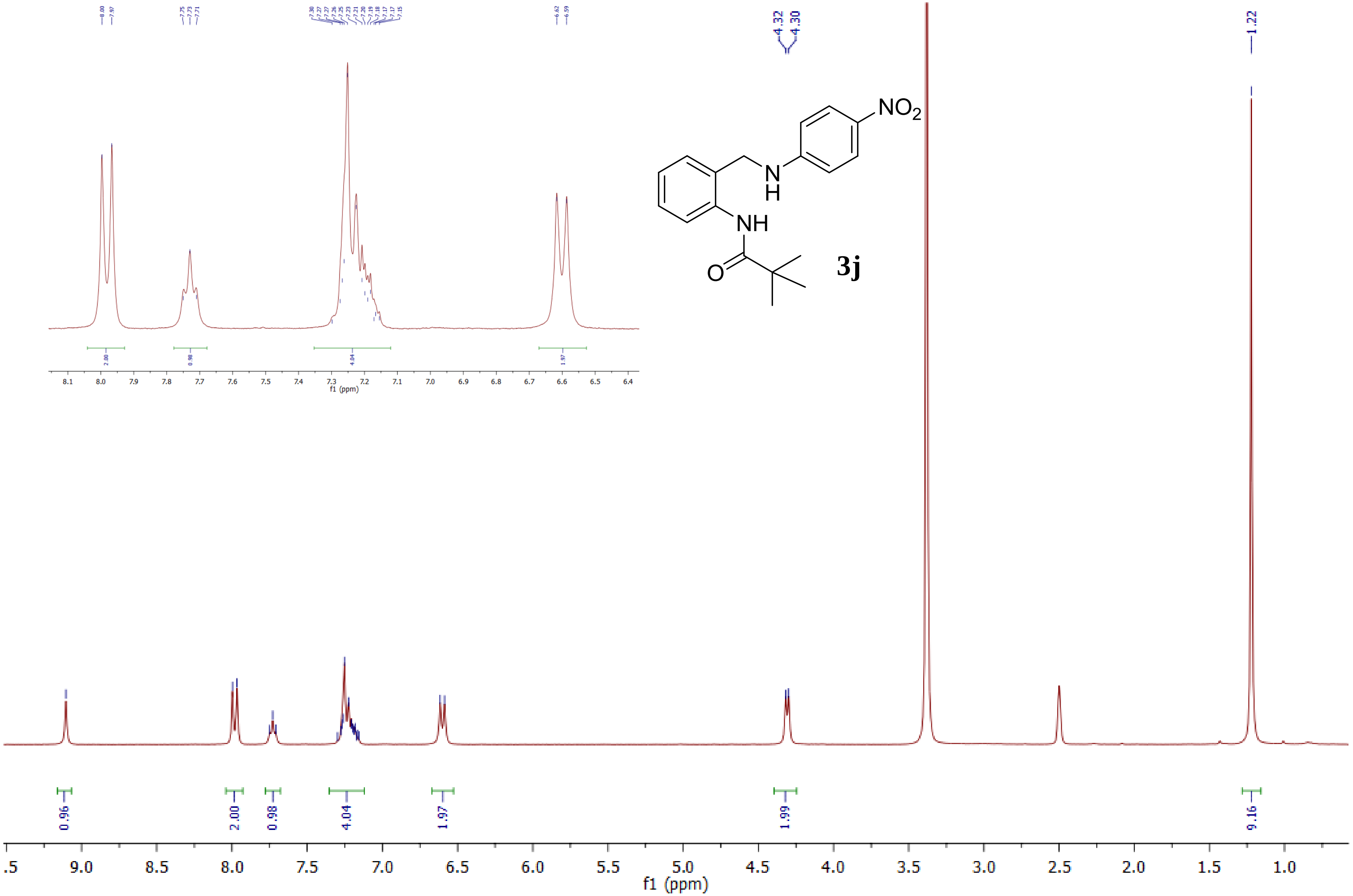

^{13}C NMR (75 MHz, DMSO-*d*6) spectrum of compound **3j**

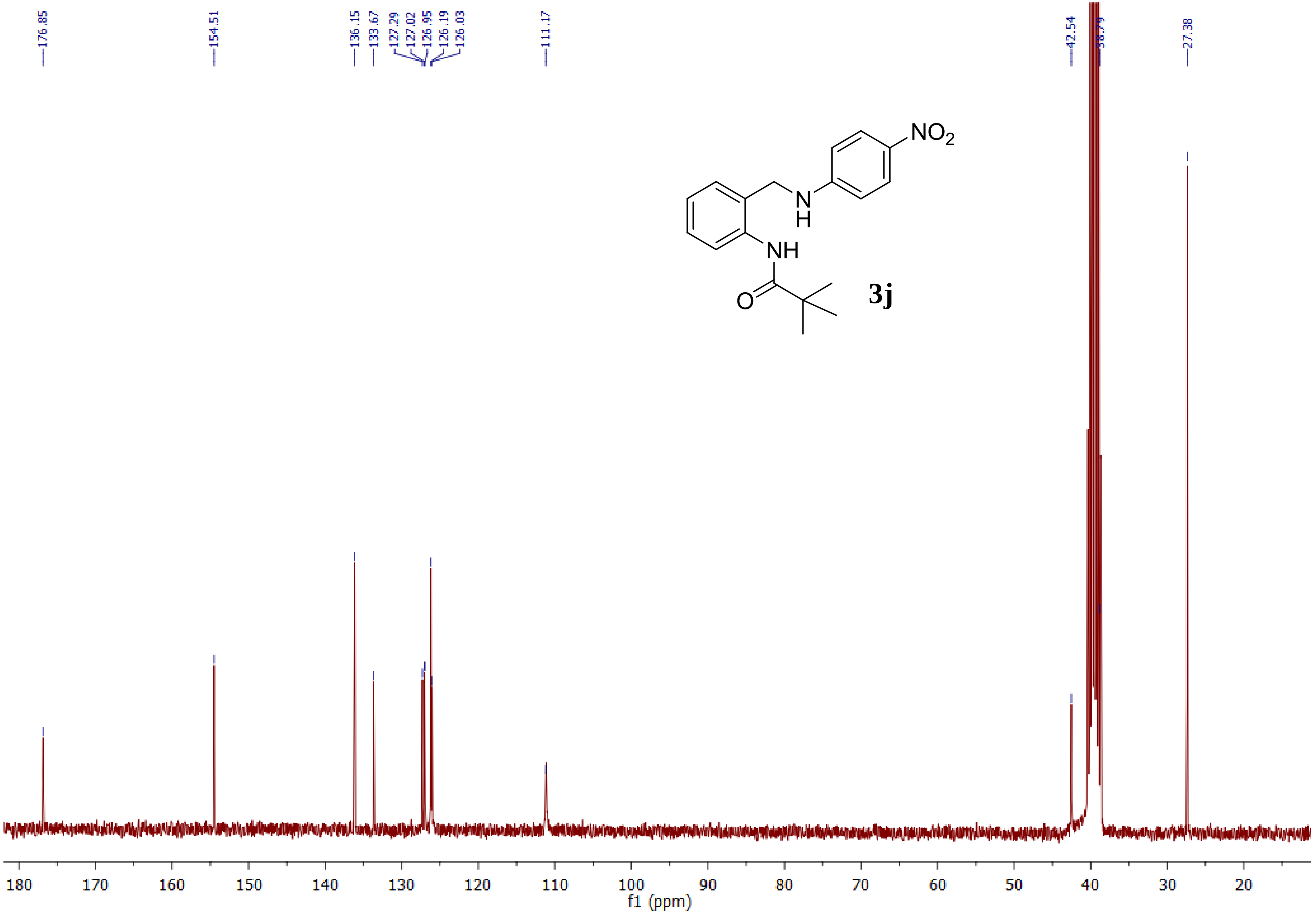

^{1}H NMR (500 MHz, DMSO-*d*6) spectrum of compound **3k**

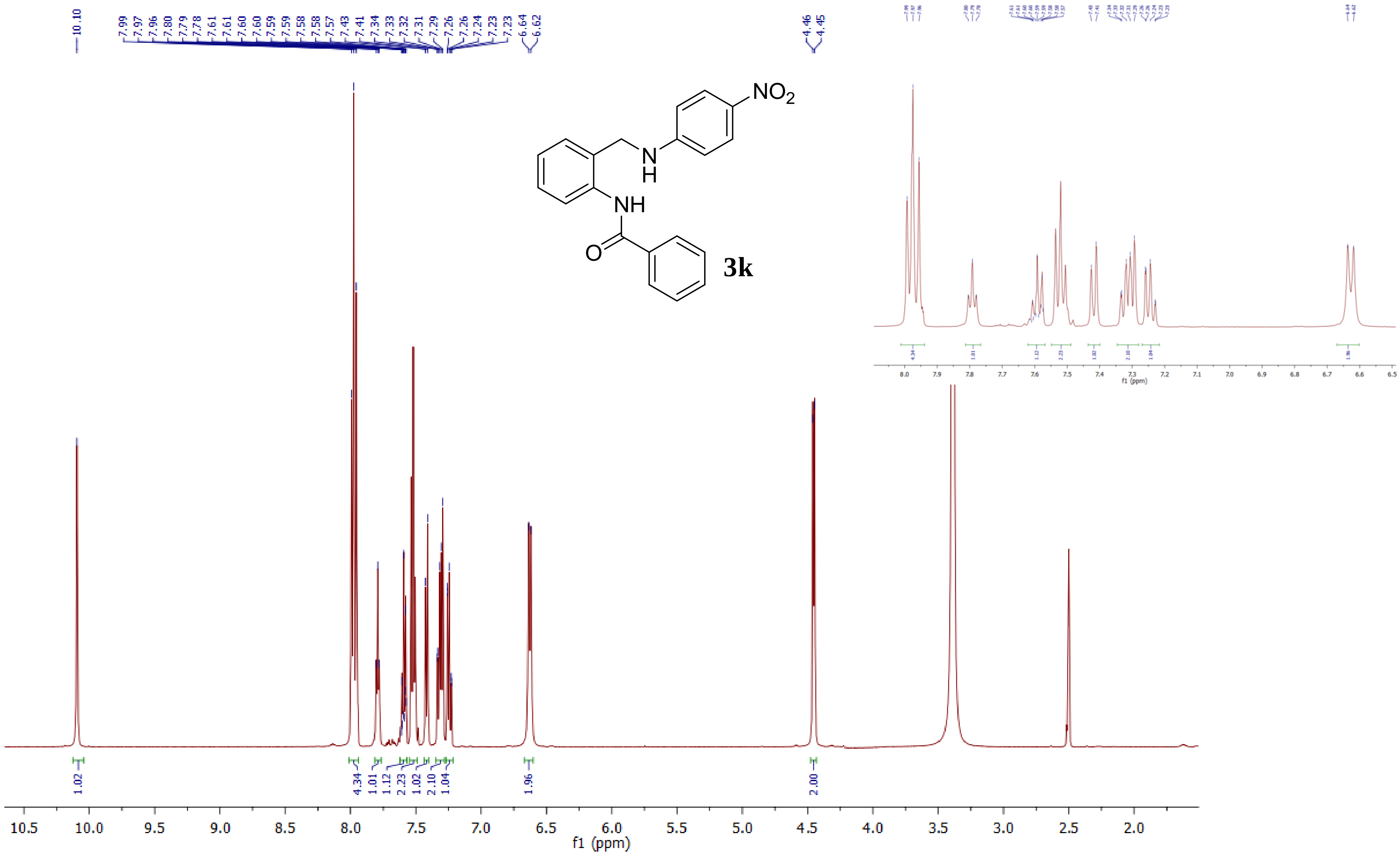

^{13}C NMR (126 MHz, DMSO-*d*6) spectrum of compound **3k**

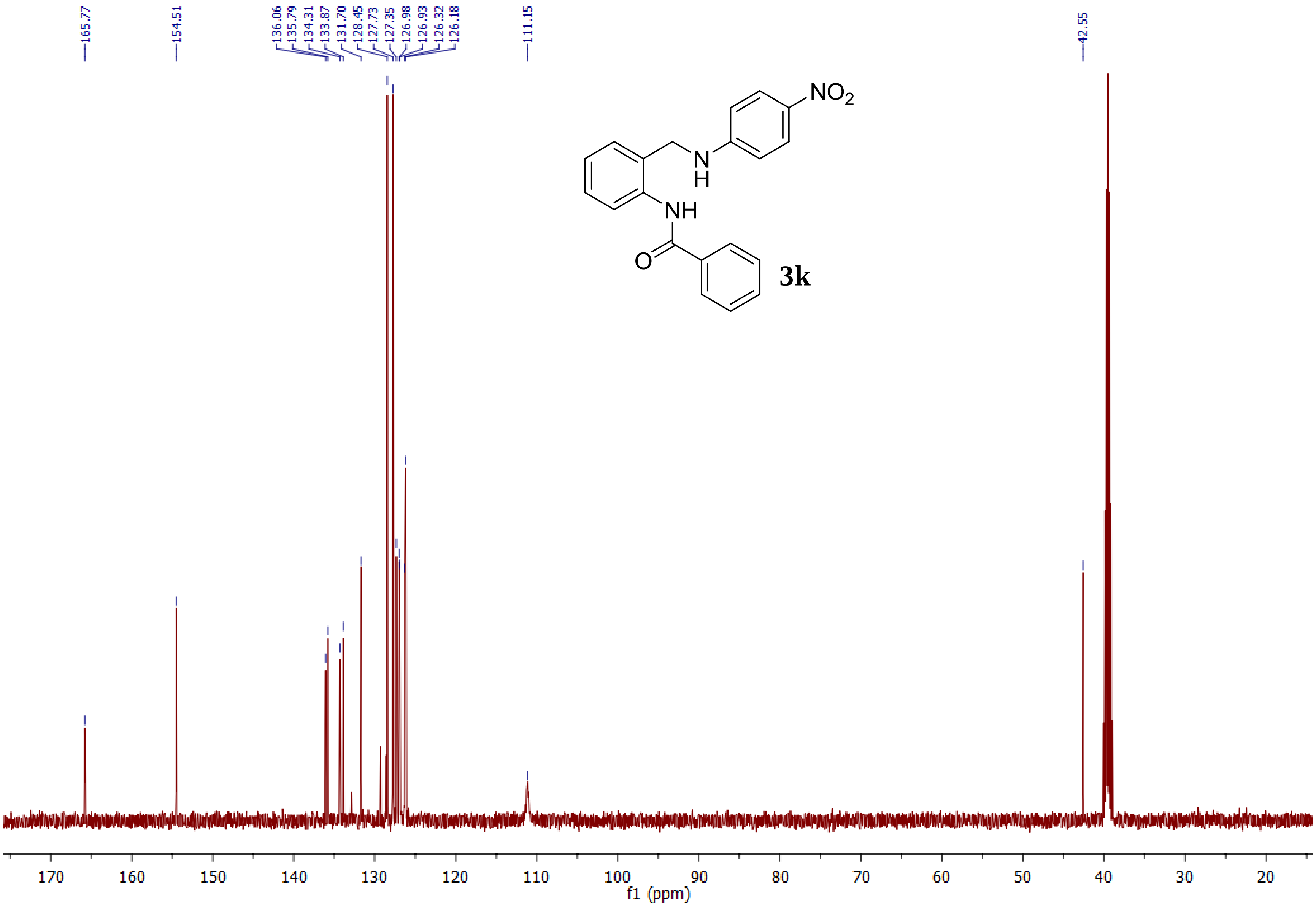

3k

^{1}H NMR (500 MHz, $CDCl_3$) spectrum of compound **3l**

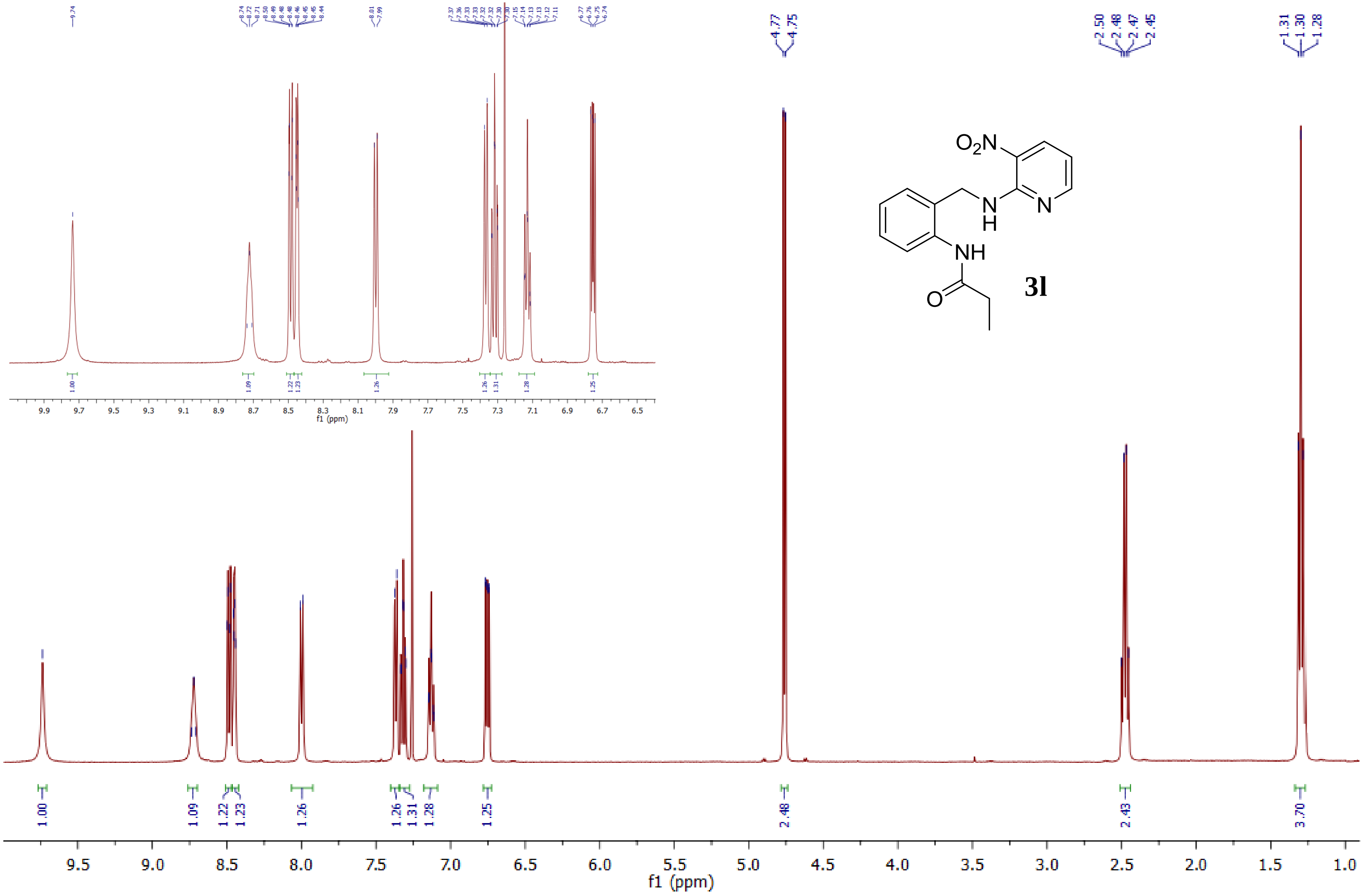

^{13}C NMR (126 MHz, $CDCl_3$) spectrum of compound **3l**

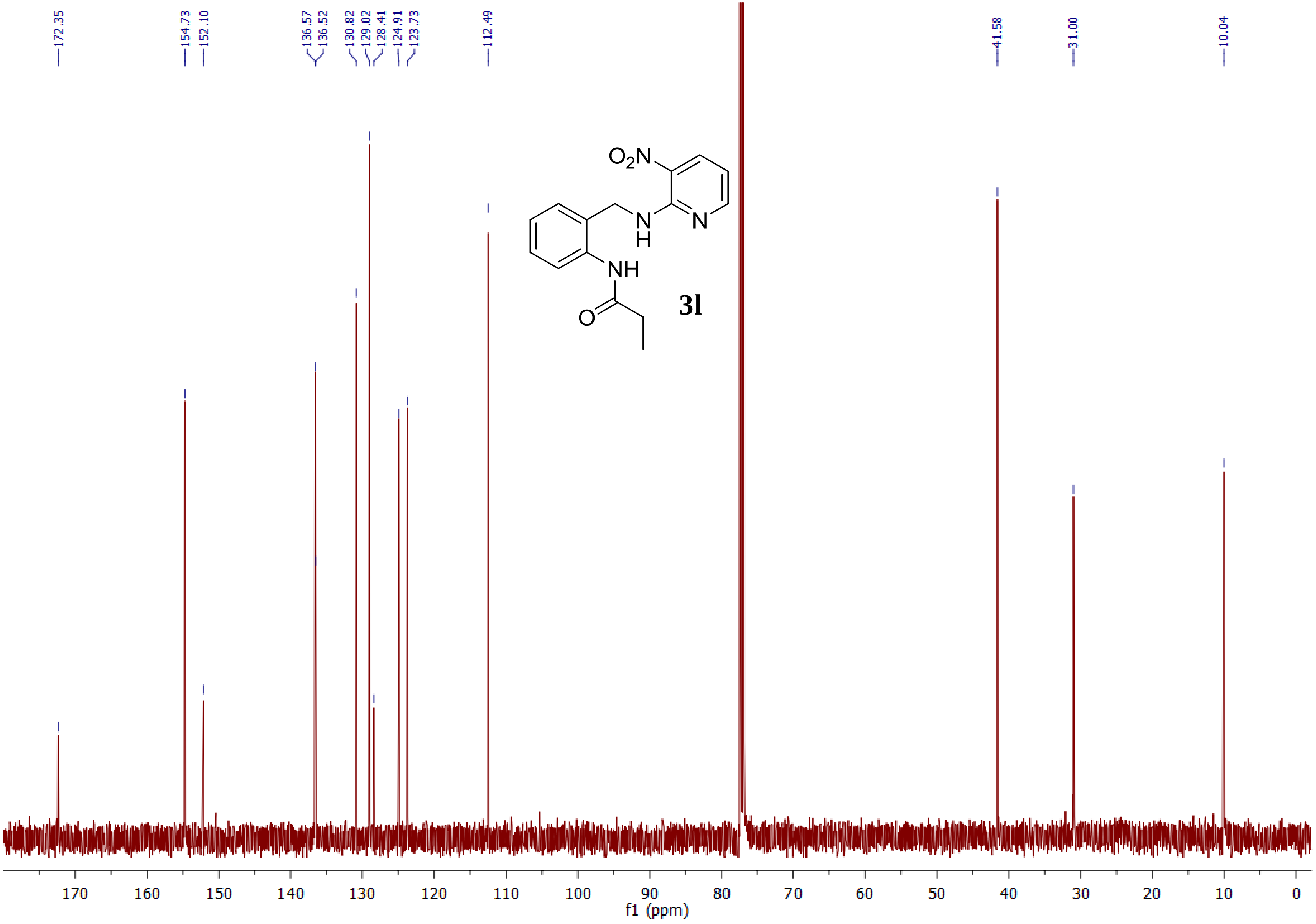

^{1}H NMR (600 MHz, $CDCl_3$) spectrum of compound **3m**

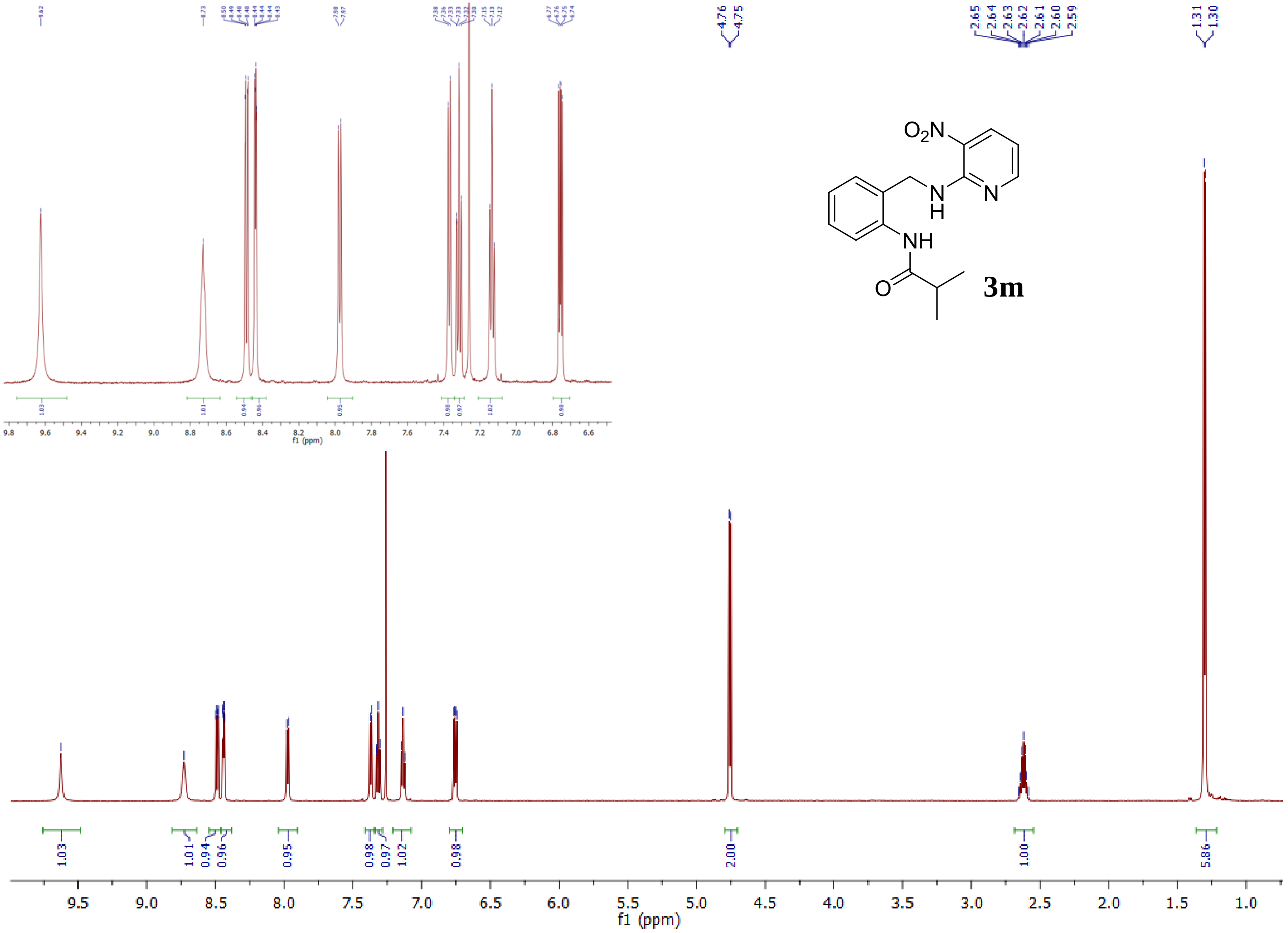

^{13}C NMR (151 MHz, $CDCl_3$) spectrum of compound **3m**

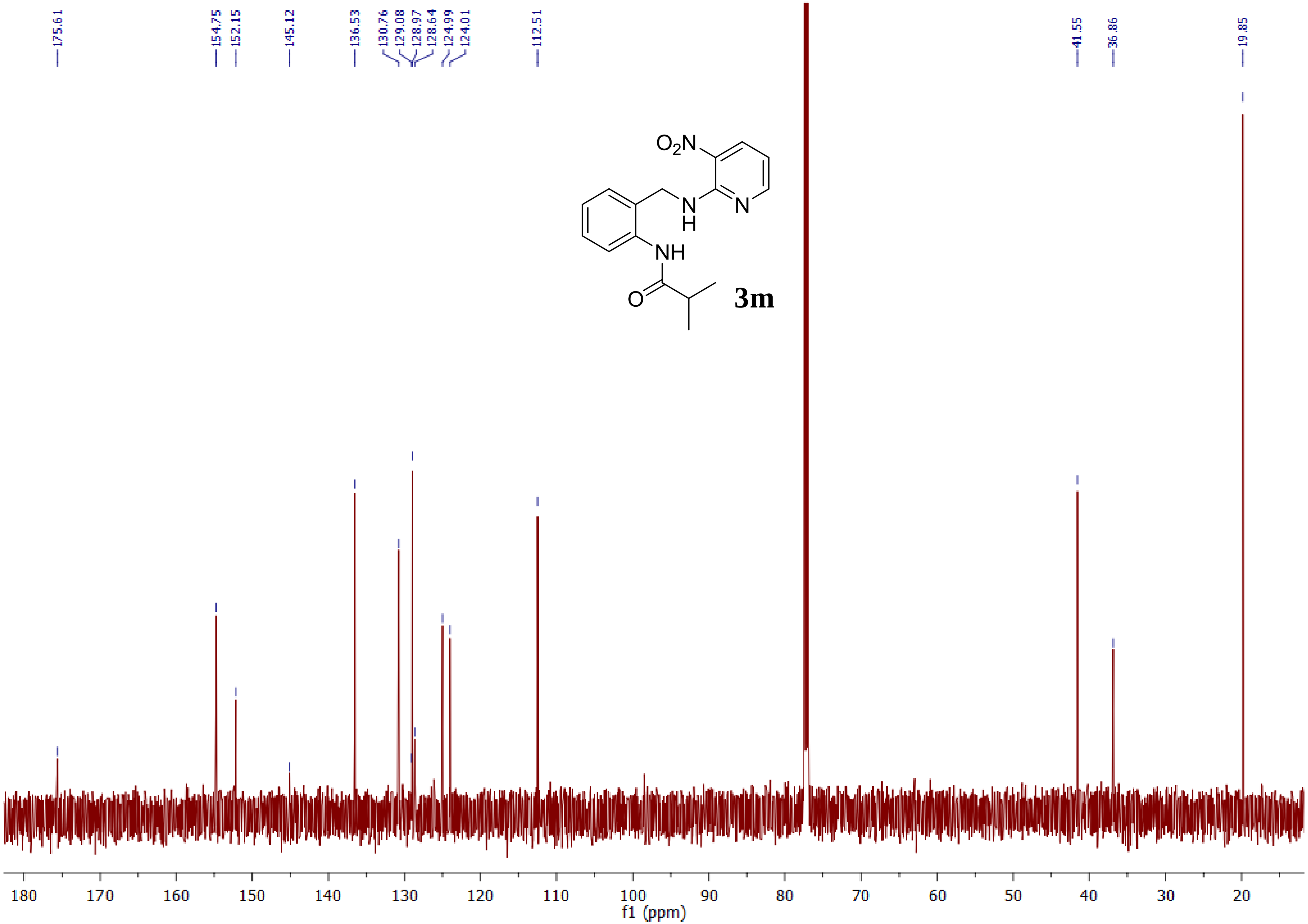

^{1}H NMR (500 MHz, $CDCl_3$) spectrum of compound **3n**

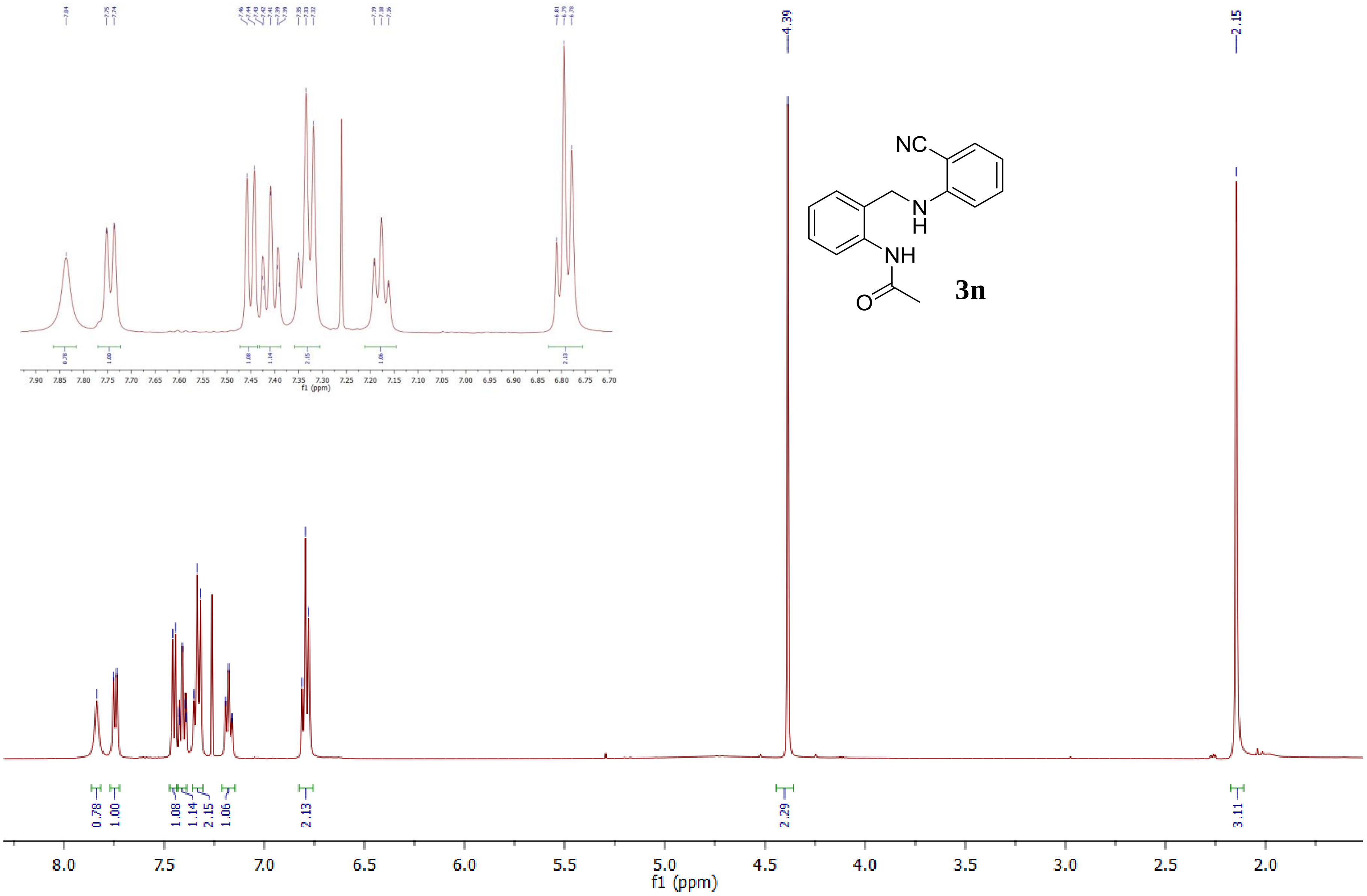

^{13}C NMR (126 MHz, $CDCl_3$) spectrum of compound **3n**

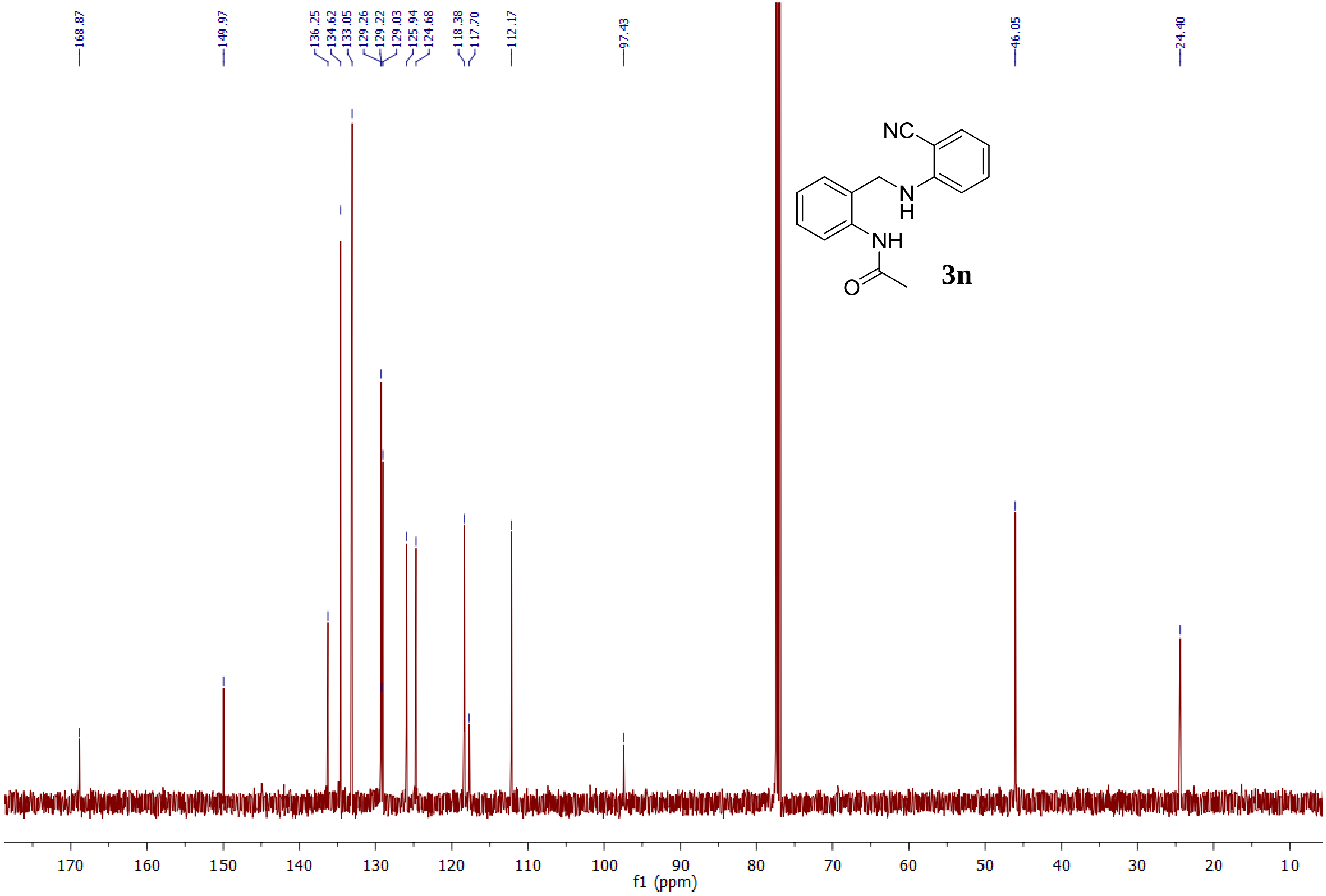

^{1}H NMR (600 MHz, $CDCl_3$) spectrum of compound **3o**

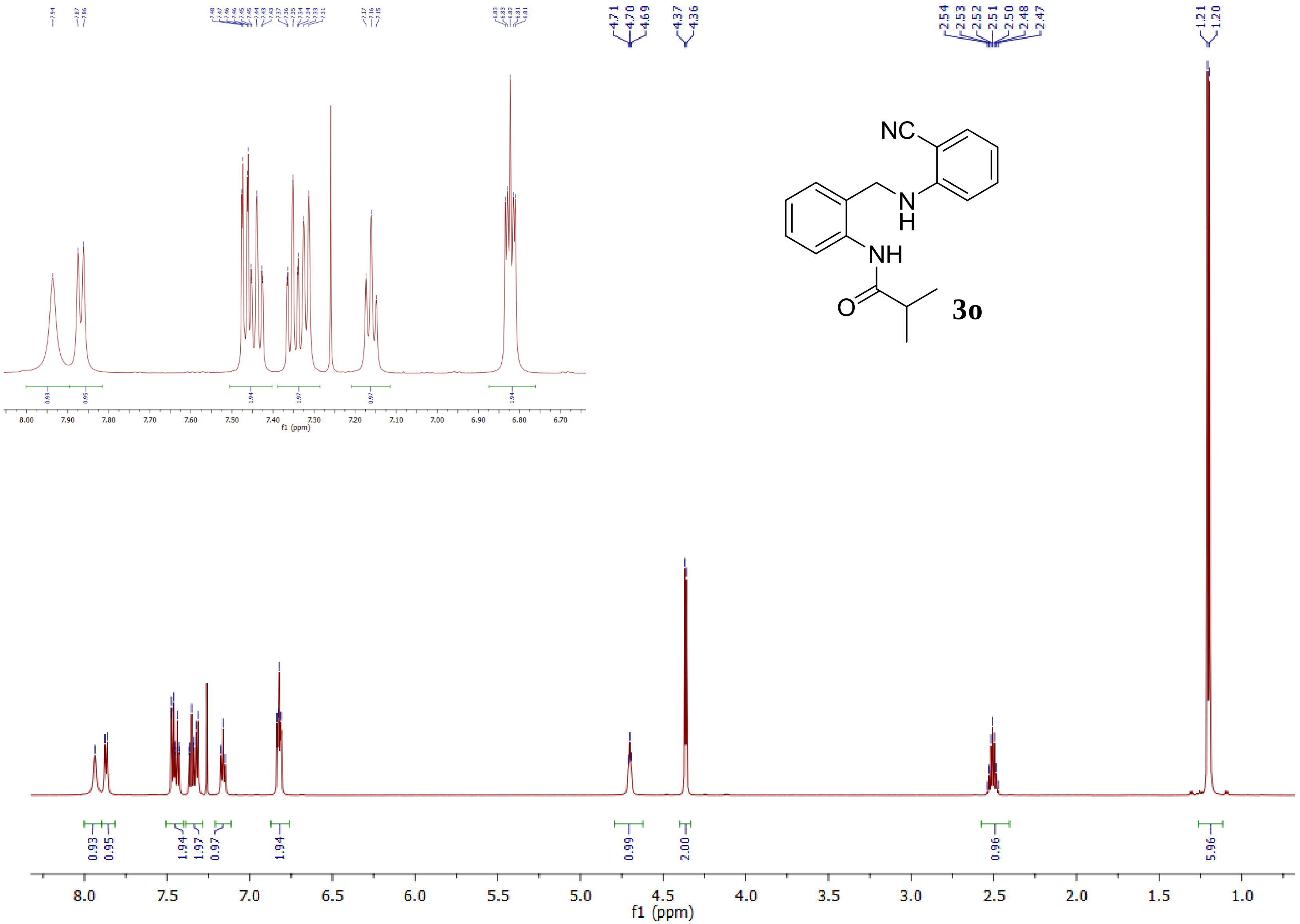

^{13}C NMR (151 MHz, $CDCl_3$) spectrum of compound **3o**

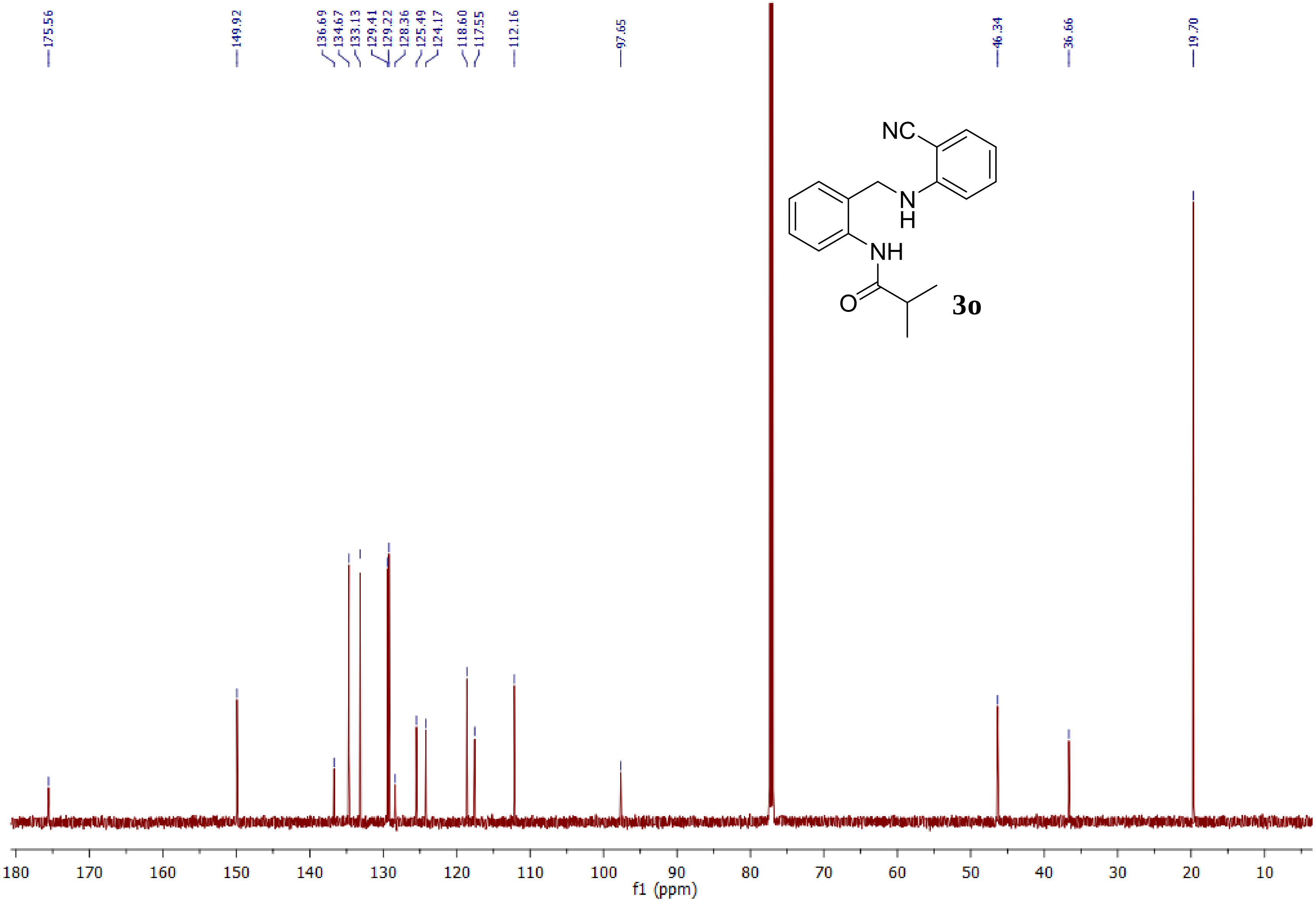

Supplement: File 1 — Experimental procedures and characterization of new compounds. [file Beilstein_J_Org_Chem-14-2510-s001.pdf]
